# Herbal Medicines for Weight Loss and Lipid Profile Improvement: A Scoping Review of Therapeutic Effects and Safety

**DOI:** 10.1002/ptr.70072

**Published:** 2025-09-14

**Authors:** Marcela Forgerini, Geovana Schiavo, Osvaldo Galo Neto, Gabriela Barbosa Nascimento, Johnny Wallef Leite Martins, Patrícia de Carvalho Mastroianni

**Affiliations:** ^1^ Department of Drugs and Medicines School of Pharmaceutical Sciences, São Paulo State University (UNESP) Araraquara Brazil

**Keywords:** anti‐obesity agents, herbal preparations, patient safety, phytotherapeutic drugs, weight reductions

## Abstract

The demand for alternative weight loss therapies has surged, with herbal medicinal products gaining popularity due to their natural origins, perceived safety, and accessibility. However, evidence regarding their therapeutic effects and safety for weight loss remains limited. Thefore, this study aimed to identify herbal medicines used for weight loss and their efficacy and safety. A scoping review was conducted using PubMed, Embase, Scopus, and LILACS databases (September 2023). Studies evaluating the efficacy and safety of herbal medicines for weight loss in individuals aged 18 years or older were included. Study selection and data extraction were performed independently by two reviewers. A total of 74 studies involving 5508 participants were included. Most studies were double‐blind randomized clinical trials (*n* = 50) and conducted in participants diagnosed with overweight and/or obesity (*n* = 70), predominantly in Asia (*n* = 40). The duration of interventions ranged from 10 to 613 days. The most frequently evaluated herbal medicines were *Camellia sinensis* (*n* = 16), *Ephedra* sp. (*n* = 13), *Garcinia cambogia* (*n* = 9), *Citrus* sp. (*n* = 5), and *Glycyrrhiza* sp. (*n* = 5). Five studies conducted post‐intervention follow‐ups. Only 10 studies did not report improvements in any parameter. Adverse events ranged from mild symptoms (e.g., headache) to severe reactions (e.g., increased blood pressure), with *Ephedra* sp. being linked to the most serious events. In conclusion, herbal medicinal products may offer benefits for weight loss. However, their use should be approached cautiously due to potential safety concerns.

## 1 Introduction

Obesity and overweight are highly prevalent health conditions, affecting approximately 813 million adults worldwide and imposing substantial economic burdens on health systems—amounting to approximately USD 1.96 trillion globally in 2020 (World Obesity Federation 2023) and BRL 1.39 billion for the Brazilian health system (Nilson et al. 2020). These conditions are recognized as major risk factors for various diseases, including cardiovascular and metabolic disorders (World Obesity Federation 2023) and contribute to an estimated 2.8 million deaths annually due to obesity‐related complications (World Health Organization 2021).

Weight loss is typically achieved through lifestyle modifications, such as physical exercise, dietary changes, and behavioral therapy, which are regarded as effective long‐term interventions (Bray et al. 2016). Depending on the patient's clinical condition, pharmacological and surgical approaches may also be necessary (Bray et al. 2016).

In pursuit of both health and aesthetic goals, there has been a rising demand for rapid and effortless weight loss strategies, often without adequate consideration of potential health risks (Melguizo‐Ibáñez et al. 2023). Consequently, the demand for alternative therapies has surged, with herbal medicinal products gaining prominence due to their natural origin, perceived safety, and accessibility (Farrington et al. 2019). Furthermore, the use of herbal medicines is an ancestral healthcare practice, often adopted in contexts where access to conventional health services is limited (Sandes et al. 2018).

Notably, 2 previous systematic reviews (2004 and 2015) highlighted that most herbal medicinal products commonly used by the Brazilian population lack robust scientific evidence supporting their efficacy for weight loss (Pittler and Ernst 2004; Cercato et al. 2015). Conversely, four other reviews identified herbal medicinal products associated with favorable outcomes in reducing anthropometric indices in humans and animals (Payab et al. 2020; Hasani‐Ranjbar et al. 2009; Maunder et al. 2020; Park et al. 2022). These systematic reviews focused on specific populations (e.g., individuals diagnosed with overweight, obesity, or metabolic syndrome) and study designs (e.g., randomized clinical trials), while insufficiently addressing the safety aspects associated with the use of herbal medicinal products for weight loss. Thus, a comprehensive analysis addressing both the therapeutic effects and safety issues of herbal medicinal products for weight loss remains lacking in the literature.

In parallel with growing evidence on the efficacy of herbal medicines for weight loss, reports of health incidents associated with their use, including adverse events, have also increased (Posadzki et al. 2013). For instance, the Brazilian Health Regulatory Agency (Anvisa) recently banned the sale of over 200 weight‐loss herbal products due to the lack of registration and scientific evidence of their efficacy and safety (Brasil. Agência Nacional de Vigilância Sanitária 2022a). This decision followed the death of a nurse from fulminant hepatitis induced by unregulated “weight‐loss capsules” (Brasil. Agência Nacional de Vigilância Sanitária 2023). Another example is green tea ( *Camellia sinensis* ), a widely used herbal product associated with adverse events such as gastrointestinal symptoms (e.g., nausea, abdominal pain, discomfort, and diarrhea), elevated liver enzyme levels, and potential hepatotoxicity (Hu et al. 2018).

Considering the traditional and cultural use of herbal medicines in healthcare and the increasing popular and scientific interest in their application for weight loss (Farrington et al. 2019), a scoping review was conducted to map the therapeutic effects of herbal medicinal products, identify their associated anthropometric and biochemical outcomes, and potential safety concerns.

## 2 Methods

### 2.1 Study Design

A scoping review was conducted following the guidelines provided by the Joanna Briggs Institute (Peters et al. 2015). The report of this study adhered to the Preferred Reporting Items for Systematic Reviews and Meta‐Analyzes extension for Scoping Reviews (PRISMA‐ScR) (Tricco et al. 2018), as detailed in Table S1.

### 2.2 Scoping Review Questions

This scoping review was guided by the following 2 primary questions:

1. *Which herbal medicine products are used for weight loss?*
2. *What are the therapeutic effects and adverse events associated with the use of herbal medicines for weight loss*?

### 2.3 Eligibility Criteria

Studies were included based on the PCC (population, concept, and context) framework:

*Population* (*P*): adults (≥ 18 years old) regardless of sex or health condition.

*Concept* (*C*): studies evaluating the use of herbal medicines products for weight loss, including their therapeutic effects and safety. Studies that did not assess weight loss as a primary outcome were excluded from the review.

*Context* (*C*): no restrictions on country, city, setting, level of healthcare, or service type (e.g., hospital). Studies funded by the pharmaceutical industry were also considered.

Editorials, commentaries, news, articles, conference abstracts, reviews, and studies published in non‐Roman alphabets (e.g., Russian, Japanese, and Chinese) were excluded. There were no restrictions on the publication date.

### 2.4 Information Sources and Search

Electronic searches were conducted in Embase, Medline (PubMed), LILACS (Latin American and Caribbean Health Sciences Literature), and Scopus (Elsevier), covering all articles published up to September 2023. The search strategy was adapted for each database and is described in Table S2.

### 2.5 Selection of Sources of Evidence

The studies retrieved from the electronic searches were imported into the Rayyan platform (https://rayyan.qcri.org), and duplicates were removed. Two researchers (O.G.N. and G.B.N.) independently performed the screening process (reading of titles and abstracts) and the eligibility assessment (full‐text article review). Disagreements between the reviewers at any stage of the selection process were resolved through consensus discussions, with the involvement of a third and fourth researcher (M.F. and G.S.).

In addition to the studies identified through electronic searches, the previously identified reviews on the topic of interest and the reference lists of the included articles were manually reviewed by the same two independent reviewers (O.G.N. and G.B.N.). The reasons for the exclusion of studies during the eligibility phase are provided in Table S3.

### 2.6 Definition of Herbal Medicines

The following definitions were used to consider herbal medicine products:

– Brazilian Health Regulatory Agency (Anvisa): “Medicinal plants, plant drugs, plant derivatives, and herbal medicines” (Brasil. Ministério da Saúde. Agência Nacional de Vigilância Sanitária 2014).
– European Medicines Agency (EMA): “Any medicinal product, exclusively containing as active ingredients one or more herbal substances or one or more herbal preparations, or one or more such herbal substances in combination with one or more such herbal preparations” (European Medicines Agency 2023).
– Food and Drug Administration (FDA): “Product taken by mouth that contains a dietary ingredient intended to supplement the diet (vitamins, minerals, herbs or other botanicals, amino acids, and substances such as enzymes, organ tissues, glandular, and metabolites)” (Food and Drug Administration 2022).
– National Medical Products Administration (NMPA): “Products that contain Chinese medicinal plant raw materials and are processed according to traditional Chinese methods” (The National Medical Products Administration 2023).

### 2.7 Outcomes

#### 2.7.1 Primary Outcomes

The primary outcome was the efficacy of herbal medicine products in improving anthropometric parameters, including body weight, body composition, body mass index (BMI), body fat mass, fat‐free mass, skinfold thickness, waist‐hip ratio, and circumferences of thigh, chest, arm, biceps, hip, waist, neck, and upper abdomen. Safety outcomes were identified and described based on the adverse events reported by the authors of the primary studies.

#### 2.7.2 Secondary Outcomes

The secondary outcome involved identifying and describing the effects of herbal medicines on biochemical parameters, including lipid and glycemic profiles, as well as other parameters related to weight loss.

### 2.8 Data Charting Process and Items

The data extraction was performed using Microsoft Office Professional Plus Excel (2019, Microsoft Corporation, Redmond, WA, USA) by 2 independent reviewers (O.G.N. and G.B.N.). The following data were extracted:

- *Study characteristics*: study design, country, setting, inclusion and exclusion criteria, study groups, intervention details, duration of follow‐up, assessed outcomes, number of participants lost to follow‐up, reasons for participant losses, adherence to the intervention protocol, ethical considerations, funding sources, and conflicts of interest.
- *Population characteristics*: number of participants, sex, age, health conditions, and concurrent pharmacotherapy.
- *Herbal medicines*: scientific and/or common (popular) name, preparation method (e.g., infusion) or pharmaceutical form (e.g., capsules), dosage regimen, duration of use, whether the product is industrialized, and its patent status.
- *Outcomes*: therapeutic effects, reported adverse events, precautions for use, and the methods used to assess these outcomes.

### 2.9 Critical Appraisal of Individual Sources of Evidence

In accordance with the guidelines of the *Joanna Briggs Institute* guidelines (Peters et al. 2015), we did not assess the methodological quality of studies included. Scoping reviews aim to map the breadth of evidence on a specific topic, without focusing on the quality of evidence.

### 2.10 Synthesis of Results

The results related to study characteristics, participants, interventions, and outcomes are presented narratively, with Supporting Information figures or tables where appropriate.

## 3 Results

### 3.1 Study Selection

After removing duplicates, 1797 studies were identified through electronic databases, with an additional 42 studies found via manual search (*n* = 1839). Of these, 1719 studies were deemed irrelevant during initial screening. Full text was not accesscible of one article. A total of 119 studies were assessed in full. Forty‐five were excluded during the full‐text review for the following reasons: concept (*n* = 31), study design (*n* = 10), language (*n* = 3), and population (*n* = 1) (Figure S1). Seventy‐four studies were included. Detailed screening and eligibility steps are provided in Figure S1 and Table S3.

### 3.2 Characteristics of Studies and Population

The studies were published between 1983 and 2023, with the majority being double‐blind randomized clinical trials (*n* = 50). The studies were conducted in various regions, including Asia (*n* = 40), North America (*n* = 22), Europe (*n* = 10), and Oceania (*n* = 2), with the United States (*n* = 20), South Korea (*n* = 11), and India (*n* = 9) being the most common locations.

The studies included a total of 5508 participants, with sample sizes ranging from 9 to 270. Most participants were female (*n* = 3598) and the studies predominantly focused on individuals diagnosed with overweight and/or obesity (*n* = 70). Industrialized herbal medicinal products were evaluated in 56 studies. Funding from pharmaceutical companies or authors affiliated with these companies was reported in 39 studies, 13 of which explicitly disclosed conflicts of interest (Bagchi et al. 2022; Bell et al. 2011; Cao et al. 2023; Cho et al. 2017; Chong et al. 2014; Dixit et al. 2018; Hancke et al. 2021; Kamohara and Noparatanawong 2013; Landor et al. 2015; Leverrier et al. 2019; Lopez et al. 2013; Sindler and Sindler 2001; Woodgate and Conquer 2003).

Detail descriptions of the study design, inclusion criteria, setting, and total of participants, as well as funding, ethical considerations, pharmacy industry involvement in the intervention, and conflicts of interest are provided in Tables S4 and S5, respectively.

### 3.3 Characteristics of Interventions

The most frequently evaluated herbal medicines were *Camellia sinensis* (*n* = 16), *Ephedra* sp. (*n* = 13), *Garcinia cambogia* (*n* = 9), *Citrus* sp. (*n* = 5), and *Glycyrrhiza* sp. (*n* = 5). Herbal medicines were administered orally in different forms, including capsules, sachets diluted in water, decoctions, and infusions. Fourteen studies combined herbal medicines with dietary supplements (Bagchi et al. 2022; Lopez et al. 2013; Sindler and Sindler 2001; Woodgate and Conquer 2003; Armstrong et al. 2001; Badmaev et al. 2001; Belcaro et al. 2009; Boozer et al. 2001; Cai et al. 2017; González et al. 2004; Greenway, Liu, et al. 2006; Hackman et al. 2006; Khazaal et al. 2015; Preuss et al. 2004). Twenty‐two studies incorporated dietary therapy and physical exercise into the intervention (Cho et al. 2017; Dixit et al. 2018; Hancke et al. 2021; Lopez et al. 2013; Belcaro et al. 2009; Khazaal et al. 2015; Greenway et al. 2004; Hioki et al. 2004; Kazemipoor et al. 2013; Lee et al. 2014; Sengupta et al. 2012; Stendell‐Hollis et al. 2010; Stern, Peerson, Mishra, Sadasiva Rao, and Rajeswari 2013; Stern, Peerson, Mishra, Mathukumalli, and Konda 2013; Sukohar et al. 2017; Tripathy et al. 2013; Astrup et al. 1992; Auvichayapat et al. 2008; Boozer et al. 2002; Kang et al. 2018; Kim et al. 2008). The most common supplements included L‐carnitine (Sindler and Sindler 2001; Armstrong et al. 2001; González et al. 2004; Greenway, Liu, et al. 2006; Khazaal et al. 2015), vitamins (e.g., B and C complexes) (Lopez et al. 2013; Sindler and Sindler 2001; Boozer et al. 2001; Cai et al. 2017; Greenway, Liu, et al. 2006; Hackman et al. 2006), chromium (Lopez et al. 2013; Badmaev et al. 2001; Boozer et al. 2001; González et al. 2004; Preuss et al. 2004), and magnesium (Sindler and Sindler 2001; Armstrong et al. 2001; Boozer et al. 2001).

Forty‐one studies evaluated the efficacy of herbal medicine products by comparing pre‐ and post‐intervention outcomes between study groups (herbal medicine vs. control). Twenty‐three studies did not compare results between study groups (Bell et al. 2011; Cao et al. 2023; Armstrong et al. 2001; Belcaro et al. 2009; Boozer et al. 2001; Khazaal et al. 2015; Preuss et al. 2004; Auvichayapat et al. 2008; Di Pierro et al. 2009; Andersen and Fogh 2001; Said et al. 2011; Ignjatovic et al. 2000; Blom et al. 2011; Chan et al. 2006; Dellalibera et al. 2006; Diepvens et al. 2006; Egert et al. 2009; Henderson et al. 2005; Hsu et al. 2008; Kim et al. 2015; Nagao et al. 2009; Tominaga et al. 2009; Kamiya et al. 2011) and 8 studies conducted before‐and‐after analyses within the herbal medicine group, using participants as their own controls (Kamohara and Noparatanawong 2013; Badmaev et al. 2001; Sukohar et al. 2017; Kang et al. 2018; Gupte et al. 2020; Hu et al. 2012; Kim et al. 2014; Frati‐Munari et al. 1983).

Ten studies lacked a control group. In 3 studies, participants in the control groups did not receive a placebo or other products (Khazaal et al. 2015; Di Pierro et al. 2009; Gupte et al. 2020). In 5 studies, control group participants received an active placebo, such as a dietary supplement (González et al. 2004), *Hoodia parviflora* (Landor et al. 2015), *Bofutsushosan* (Lee et al. 2014), or a low dose of the intervention—*Xin‐Ju‐Xiao‐Gao‐Fang* (Zhou et al. 2014) or catechin (Nagao et al. 2009). In 9 studies, participants maintained the use of pharmacotherapy during the intervention, including antihypertensives, antidiabetics, and lipid‐lowering agents (Badmaev et al. 2001; Stendell‐Hollis et al. 2010; Kang et al. 2018; Said et al. 2011; Egert et al. 2009; Nagao et al. 2009; Frati‐Munari et al. 1983; Liu et al. 2022).

The duration of the studies ranged from 10 days (Frati‐Munari et al. 1983) to 613 days (Greenway et al. 2004). Five studies conducted post‐intervention follow‐ups to assess the efficacy (maintenance of anthropometric parameters) and safety of the herbal medicines. Follow‐up periods ranged from 2 days (Blom et al. 2011) to 56 days (Nagao et al. 2009), including studies with durations of 26 days (Tominaga et al. 2009) and 30 days (Gupte et al. 2020; Salunke et al. 2019).

Participant withdrawals were reported in 58 studies, with the number of dropouts ranging from 1 (Preuss et al. 2004; Sukohar et al. 2017; Liu et al. 2022) to 101 (Landor et al. 2015), totaling 898 dropouts across all studies. Among these, 24 studies identified adverse events as the reason for participant withdrawal (Armstrong et al. 2001; Boozer et al. 2001, 2002; Cai et al. 2017; Greenway, Liu, et al. 2006; Hackman et al. 2006; Khazaal et al. 2015; Greenway et al. 2004; Hioki et al. 2004; Lee et al. 2014; Stendell‐Hollis et al. 2010; Kim et al. 2008, 2015; Blom et al. 2011; Chan et al. 2006; Egert et al. 2009; Hsu et al. 2008; Tominaga et al. 2009; Hu et al. 2012; Zhou et al. 2014; Coffey et al. 2004; Mirtaheri et al. 2015; Park et al. 2013; Cheon et al. 2020). Dropouts were not reported in 8 studies (Bagchi et al. 2022; Bell et al. 2011; Kamohara and Noparatanawong 2013; Belcaro et al. 2009; Tripathy et al. 2013; Auvichayapat et al. 2008; Ignjatovic et al. 2000; Henderson et al. 2005) and no information was provided in another 8 studies (Sindler and Sindler 2001; Di Pierro et al. 2009; Andersen and Fogh 2001; Dellalibera et al. 2006; Diepvens et al. 2006; Frati‐Munari et al. 1983; Greenway, de Jonge‐Levitan, et al. 2006; Kovacs et al. 2001).

Tables 1 and 2 present the study groups and interventions.

**TABLE 1:** Description of study groups, herbal medicines evaluated, and period of intervention in studies using herbal medicines alone (*n* = 51).

Groups of study (*N*)	Intervention group	Control group	Time period	References
Intervention (NR) and control (NR) groups: women diagnosed with obesity.	1 pill of 200 mg of caffeine and 20 mg of ephedrine, 1 h before breakfast, lunch, and dinner.	1 placebo pill, 1 h before breakfast, lunch, and dinner.	8 weeks	(Astrup et al. 1992)
Intervention (*n* = 66) and control (*n* = 69) groups: overweight participants.	2 pills of 500 mg of *Garcinia cambogia* extract (50% hydroxycitric acid), 30 min before 3 meals and a high‐fiber diet plan (5040 kJ/day) containing 50% of carbohydrates, 30% of protein, and 20% of fat.	2 placebo pills, 30 min before 3 meals and a high‐fiber diet plan (5040 kJ/day) containing 50% of carbohydrates, 30% of protein, and 20% of fat.	12 weeks	(Heymsfield et al. 1998)
Intervention (*n* = 70) and control (*n* = 70) groups: healthy participants.	10 drops of *Hordeum vulgare* L., *Polygonatum multiflorum* (L.) All., *Dimocarpus longan* Lour., *Ligusticum sinense* Oliv., *Lilium brownii* F.R. Br. ex Miellez, *Gynura pinnatifida*, *Coreopsis lanceolata* , *Juniperus communis* , and *Zingiber officinale* Roscoe (ratio 750:50:100:250:500:500:50:50:25 and extracted in 6.5 mM ethanol). The drops were diluted in a glass of water before breakfast, lunch, and dinner. The daily dosage was calculated to be 40 mg of dry body weight per person.	10 placebo drops (40 mg of dry body weight) diluted in a glass of water before breakfast, lunch, and dinner.	6 weeks	(Ignjatovic et al. 2000)
Intervention (*n* = 22) and control (*n* = 22) groups: overweight participants.	3 capsules of 112 mg of Yerbe Maté extract ( *Ilex paraguayensis* ), 95 mg of Guarana seeds ( *Paullinia cupana* ), and 36 mg of Damiana leaves ( *Turnera diffusa var* . *aphrodisiaca* ) with 20 mL apple juice. After 15 min, 400 mL of apple juice was also ingested.	3 placebo capsules with 20 mL apple juice. After 15 min, 400 mL of apple juice was also ingested.	12 months	(Andersen and Fogh 2001)
Intervention (*n* = 35) and control (*n* = 32) groups: overweight participants.	2 pills of 40 mg of caffeine from guarana and 12 mg of ephedrine alkaloids from *Ma Huang*, 30 min before breakfast, lunch, and dinner. Moderate physical activity and limiting fat intake (10% of calories) were suggested.	2 placebo pills, 30 min before breakfast, lunch, and dinner. Moderate physical activity and limiting fat intake (10% of calories) were suggested.	8 weeks	(Boozer et al. 2001)
Intervention group (*n* = 21): participants diagnosed with overweight or obesity.	*Time 1*: 1 snack and 500 mg of hydroxycitric acid from *Garcinia cambogia*. *Time 2*: 1 snack; 500 mg of hydroxycitric acid from *Garcinia cambogia*; and 3 g of medium chain triglycerides. *Time 3*: 1 snack. Participants received the isoenergetic snacks 1 h before lunch and dinner; and 2 h after lunch and dinner. All snacks contained 22 g of energy (420 kJ), 0.7 g of protein, 4 g of fat, 14 g of carbohydrate, and 0.5 g of dietary fiber.	NA.	2 weeks and 2 or 6 weeks of washout	(Kovacs et al. 2001)
Intervention (*n* = 83) and control (*n* = 84) groups: overweight participants.	2 pills of *Ma Huang* (90 mg of ephedrine alkaloids) and Kola nut (120 mg of caffeine) 30 min before breakfast, lunch, and dinner. Moderate physical activity and limiting fat intake (30% of calories) were suggested.	2 placebo pills 30 min before breakfast, lunch, and dinner. Moderate physical activity and limiting fat intake (30% of calories) were suggested.	6 months	(Boozer et al. 2002)
Intervention (*n* = 52) and control (*n* = 52) groups: participants diagnosed with overweight or obesity.	2 capsules of 125 mg of *Ma Huang* (10 mg of ephedrine at 8%), 250 mg of Kola nut (60 mg of caffeine at 25%), and 100 mg of White willow bark (15 mg of salicin at 15%), 3 times a day.	2 placebo capsules, 3 times a day.	12 weeks	(Coffey et al. 2004)
Intervention (*n* = 7) and control (*n* = 12) groups: overweight participants.	1 capsule of 250 mg of *Coleus forskohlii* extract (Forslean) in the morning and evening, 30 min before the meal.	1 placebo capsule in the morning and evening, 30 min before the meal.	12 weeks	(Henderson et al. 2005)
Intervention (*n* = 18) and control (*n* = 16) groups: participants diagnosed with obesity and polycystic ovary syndrome.	6 capsules of 90 mg of Chinese green tea leaves, 3 times a day. Diet and abstaining from drinking or eating foods containing caffeine were suggested.	6 placebo capsules, 3 times a day. Diet and abstaining from drinking or eating foods containing caffeine were suggested.	3 months	(Chan et al. 2006)
Intervention (*n* = 30) and control (*n* = 20) groups: overweight participants.	1 capsule of 200 mg of green coffee extract (Svetol) for each meal twice a day.	1 placebo capsule for each meal twice a day.	60 days	(Dellalibera et al. 2006)
Intervention (*n* = 23) and control (*n* = 23) groups: overweight participants.	3 pills of 100 mg of green tea extract before breakfast, lunch, and dinner. The participants consumed a low‐energy diet from days 4 to 87.	3 placebo pills before breakfast, lunch, and dinner. The participants consumed a low‐energy diet from days 4 to 87.	87 days	(Diepvens et al. 2006)
Intervention 1 (*n* = 6), intervention 2 (*n* = 6), intervention 3 (*n* = 6), and control (*n* = 6) groups: participants diagnosed with obesity.	*Intervention 1*: 1 pill of 250 mg of freeze‐dried. *Intervention 2*: 1 pill of 341 mg of bed dried. *Intervention 3*: 1 pill of 500 mg of freeze‐dried. All the pills contained Rhubarb root and stem (Radix et Rhizoma Rhei), astragulus root (*Radix astragali*), red sage root (Radix *Salviae miltiorrhizae*), turmeric ( *Curcuma longa* ), and ginger ( *Zingiber officinale* ). The pills were associated with a diet plan with a 700‐kcal deficit.	1 placebo pill and a diet plan with a 700‐kcal deficit.	12 weeks	(Greenway, Liu, et al. 2006)
Intervention 1 (*n* = 125) and intervention 2 (*n* = 125) groups: participants diagnosed with visceral fat‐type obesity.	*Intervention 1*: 340 mL of *Camellia sinensis* extract (583 mg catechins). *Intervention 2*: 340 mL of *Camellia sinensis* extract (96 mg of catechins).	NA.	12 weeks	(Nagao et al. 2007)
Intervention (*n* = 30) and control (*n* = 30) groups: participants diagnosed with obesity.	1 capsule of 250 mg of *Camellia sinensis* leaf extract (Herbal One) after breakfast, lunch, and dinner; and a diet plan containing of 65% of carbohydrates, 15% of protein, and 20% of fat.	1 placebo capsule after breakfast, lunch, and dinner; and a diet plan containing 65% of carbohydrates, 15% of protein, and 20% of fat.	12 weeks	(Auvichayapat et al. 2008)
Intervention (*n* = 50) and control (*n* = 50) groups: participants diagnosed with obesity.	1 capsule of 400 mg of green tea extract, 3 times a day.	1 placebo capsule, 3 times a day.	12 weeks	(Hsu et al. 2008)
Intervention 1 (*n* = 41), intervention 2 (*n* = 45), and control (*n* = 39) groups: overweight or obese premenopausal women.	*Intervention 1*: 4 capsules of 250 mg of *Ephedra sinica* powder extract. *Intervention 2*: 4 capsules of 250 mg of *Evodia rutaecarpa* powder extract. The interventions were taken 30 min after breakfast, lunch, and dinner and combined with a low‐calorie diet plan (1200 kcal/day) and moderate physical activity.	4 placebo capsules, 30 min after breakfast, lunch, and dinner; a low‐calorie diet plan (1200 kcal/day); and moderate physical activity.	8 weeks	(Kim et al. 2008)
Intervention (*n* = 50) and control (*n* = 50) groups: participants diagnosed with obesity.	1 pill of 150 mg of *Camellia sinensis* L. (Monoselect Camellia), twice a day, and a diet plan (1350 kcal/day for women or 1850 kcal/day for men).	1 placebo pill, twice a day, and a diet plan (1,350 kcal/day for women or 1850 kcal/day for men).	90 days	(Di Pierro et al. 2009)
Intervention 1 (*n* = 21), intervention 2 (*n* = 21), intervention 3 (*n* = 21), and control (*n* = 21) groups: overweight or obese postmenopausal women.	*Intervention 1*: 1 capsule of 300 mg of *Glycyrrhiza glabra* extract and 2 placebo capsules per day. *Intervention 2*: 2 capsules of 300 mg of *Glycyrrhiza glabra* extract and 1 placebo capsules per day. *Intervention 3*: 3 capsules of 300 mg of *Glycyrrhiza glabra* extract per day.	3 placebo capsules per day.	8 weeks	(Tominaga et al. 2009)
Intervention 1 (*n* = 18), intervention 2 (*n* = 15), intervention 3 (*n* = 18), and control (*n* = 18) groups: participants diagnosed with mild obesity.	*Intervention 1*: 1 pill of 100 mg of *Puerariae thomsonii* extract once a day. *Intervention 2*: 1 pill of 200 mg of *Puerariae thomsonii* extract 1 once a day. *Intervention 3*: 1 pill of 300 mg of *Puerariae thomsonii* extract 1 once a day. The interventions were combined with a diet plan (2300 kcal/day for women or 2650 kcal/day for men).	1 placebo pill, once a day and a diet plan (2300 kcal/day for women or 2650 kcal/day for men).	8 weeks	(Kamiya et al. 2011)
Intervention (*n* = 11) and control (*n* = 11) groups—study 1: participants diagnosed with overweight or obesity. Intervention (*n* = 11) and control (*n* = 11) groups—study 2: athletes.	*Study 1*: 3 capsules of 100 mg of *Glycyrrhiza glabra* L. (Glavonoid) with evening meal. *Study 2*: 3 capsules of 100 mg of *Glycyrrhiza glabra* L. (Glavonoid) with evening meal and supplementary meal (calorie load with 25% of daily intake).	Study 1: 3 placebo capsules with evening meal. Study 2: 3 placebo capsules with evening meal and supplementary meal (calorie load with 25% of daily intake).	8 weeks	(Bell et al. 2011)
Intervention (*n* = 25) and control (*n* = 24) groups: overweight participants.	Raspberry‐flavored yogurt drink with of 1110 mg of *Hoodia gordonii* purified extract, 1 h before breakfast and dinner.	Raspberry‐flavored yogurt drink, 1 h before breakfast and dinner.	15 days	(Blom et al. 2011)
Intervention group (*n* = 10): participants diagnosed with obesity.	1 capsule of 500 mg of *Coptis chinensis*, 3 times a day.	NA.	12 weeks	(Hu et al. 2012)
Intervention (*n* = 31) and control (*n* = 31) groups: participants diagnosed with obesity.	5 g of powder of *Phyllanthus emblica* L. *Terminalia chebula* Retz., and *Terminalia belerica* Retz. before breakfast and after dinner.	5 g of placebo powder before breakfast and after dinner.	3 months	(Kamali et al. 2012)
Intervention (*n* = 58) and control (*n* = 59) groups: participants diagnosed with obesity.	4 capsules of 500 mg of *Camellia sinensis* (40%) *Cassia obtusifolia* (40%), and *Sophora japonica* (20%), 3 times a day.	4 placebo capsules, 3 times a day.	12 weeks	(Lenon et al. 2012)
Intervention (*n* = 25) and control (*n* = 25) groups: participants diagnosed with obesity.	1 capsule of 500 mg of *Moringa oleifera* extract with dried leaves (60%), *Murrya koenigi* extract with dried leaves (30%), and *Curcuma longa* extract with dried rhizomes (10%), 30 min before breakfast, lunch, and dinner.	1 placebo capsule, 30 min before breakfast, lunch, and dinner.	8 weeks	(Sengupta et al. 2012)
Intervention (*n* = 30) and control (*n* = 30) groups: participants diagnosed with obesity.	2 capsules of 400 mg of *Sphaeranthus indicus* and *Garcinia mangostana* (both extracts of the flower heads), 30 min before breakfast and dinner; and a diet plan (2000 kcal/day) containing carbohydrates (61%), protein (14%), and fat (25%).	2 placebo capsules, 30 min before breakfast and dinner; and a diet plan (2000 kcal/day) containing carbohydrates (61%), protein (14%), and fat (25%).	8 weeks	(Stern, Peerson, Mishra, Sadasiva Rao, and Rajeswari 2013)
Intervention (*n* = 39) and control (*n* = 30) groups: overweight participants.	50 mL of 2.28 g of *Scutellariae Radix* extract (50%) and *Platycodi Radix* extract (50%) extract with water 3 times a day before meals.	50 mL of placebo extract with water, 3 times a day, before meals.	2 months	(Cho et al. 2013)
Intervention group (*n* = 15): healthy participants.	1 pill of 250 mg of *Coleus forskohlii* powder extract, twice a day, with 2 meals.	NA.	8 weeks	(Kamohara and Noparatanawong 2013)
Intervention (*n* = 35) and control (*n* = 35) groups: participants diagnosed with overweight or obesity	30 mL of *Carum carvi* L. (1 kg of seeds in 10 L of water), 20 min before lunch; a diet plan; and physical activity (180 min/week).	30 mL of placebo extract, 20 min before lunch.	3 months	(Kazemipoor et al. 2013)
Intervention (*n* = 50) and control (*n* = 50) groups: participants diagnosed with obesity.	2 capsules of *Sphaeranthus indicus* aqueous alcohol extracts of the flower heads and *Garcinia mangostana* fruit rinds (400 mg in 3:1 ratio, respectively) 30 min before breakfast and dinner; and a diet plan (2000 kcal/day) containing of carbohydrates (61%), protein (14%), and fat (25%).	2 placebo capsules 30 min before breakfast and dinner; and a diet plan (2000 kcal/day) containing carbohydrates (61%), protein (14%), and fat (25%).	8 weeks	(Stern, Peerson, Mishra, Mathukumalli, and Konda 2013)
Intervention (*n* = 55) and control (*n* = 55) groups: participants diagnosed with overweight or obesity	1 capsule of *Garcinia cambogia* (unreported doses) twice a day.	1 placebo capsule twice a day.	4 months	(Tripathy et al. 2013)
Intervention (*n* = 40) and control (*n* = 40) groups: participants diagnosed with obesity.	2 capsules of 225 mg of *Gynostemma pentaphyllum* a day.	2 placebo capsules a day.	12 weeks	(Park et al. 2014)
Intervention (*n* = 58) and control (*n* = 55) groups: participants diagnosed with obesity.	1 pill of 7 g of 3.75 g of *Coicis semen*, 3.75 g of *Semen castaneae*, 2.5 g of *Raphanus sativus* L., 1.25 g of *Schisandra chinensis* Baill, 1.25 g of Liriopis tuber, 1.25 g of *Ephedra*, 1.25 g of *Platycodon grandiflorus* , and 1.25 g of *Acori Tatarinowii Rhizoma* extract 3 times a day and a diet plan (1200 kcal/day for women and 1500 kcal/day for men).	7 g of placebo extract 3 times a day and a diet plan (1200 kcal/day for women and 1500 kcal/day for men).	12 weeks	(Park et al. 2013)
Intervention (*n* = 46) and control (*n* = 46) groups: overweight participants.	3 pills of 650 mg of *Garcinia cambogia* extract, 100 mg of *Camellia sinensis* extract, 75 mg of *Coffea arabica* extract, and 25 mg of *Lagerstroemia speciosa* extract 30 min before breakfast and lunch; and a diet plan with a 500‐kcal deficit.	3 placebo pills 30 min before breakfast and lunch; and a diet plan with a 500‐kcal deficit.	14 weeks	(Chong et al. 2014)
Intervention group (*n* = 9): participants diagnosed with obesity.	4 g *of Ephedra sinica* extract twice a day and caloric intake limit of 20 to 25 kcal/kg.	NA.	8 weeks	(Kim et al. 2014)
Intervention 1 (*n* = 38), intervention 2 (*n* = 40), and control (*n* = 25) groups: normal weight participants, overweight participants, and participants diagnosed with obesity.	Intervention 1: 2 lemon frozen cubes with 3 g of *Hoodia parviflora* (aerial parts) for 10 days and 2 lemon frozen cubes with 3 g of *Hoodia parviflora* (aerial parts) for 30 days. Intervention 2: 1 lemon frozen cube with 3 g of *Hoodia parviflora* (aerial parts) for 40 days.	1 lemon frozen placebo cube for 40 days.	40 days	(Landor et al. 2015)
Intervention (*n* = 70) and control (*n* = 70) groups: participants diagnosed with obesity.	170 mL of *Citrus aurantium* , rhubarb, coptis, and *Semen cassiae* decoction, twice a day.	17 mL of *Citrus aurantium* , rhubarb, coptis, and *Semen cassiae* decoction, twice a day.	24 weeks	(Zhou et al. 2014)
Intervention 1 (*n* = 20), intervention 2 (*n* = 20), and control (*n* = 20) groups: participants diagnosed with obesity.	*Intervention 1*: 1 capsule of 500 mg of Raspberry once a day. *Intervention 2*: 1 capsule of 1000 mg of L‐carnitine. Diet and physical activity were suggested.	1 placebo capsule once a day. Diet and physical activity were suggested.	12 weeks	(Khazaal et al. 2015)
Intervention (*n* = 15) and control (*n* = 15) groups: participants diagnosed with obesity.	3 capsules of 35 mg of *Ilex paraguariensis* leaves before breakfast, lunch, and dinner.	3 placebo capsules before breakfast, lunch, and dinner.	12 weeks	(Kim et al. 2015)
Intervention (*n* = 32) and control (*n* = 32) groups: participants diagnosed with overweight or obesity.	500 mg of *Glycyrrhiza glabra* extract 30 min before breakfast, lunch, and dinner; and a diet plan with a 500‐kcal deficit.	Placebo extract 30 min before breakfast, lunch, and dinner; and a diet plan with a 500‐kcal deficit.	8 weeks	(Mirtaheri et al. 2015)
Intervention (*n* = 23) and control (*n* = 21) groups: participants diagnosed with obesity.	1 pill of 700 mg of *Aster spathulifolius* Maxim powder from dried leaves 30 min after breakfast.	1 placebo pill 30 min after breakfast.	12 weeks	(Cho et al. 2016)
Intervention (*n* = 30) and control (*n* = 30) groups: overweight participants.	3 pills of 400 mg of *Imperata cylindrica* Beauvois powder extract (50%), *Citrus unshiu* Markovich powder extract (20%), and *Evodia officinalis* powder extract (30%) after breakfast and dinner; and a diet plan with a 500‐kcal deficit.	3 placebo pills after breakfast and dinner; and a diet plan with a 500‐kcal deficit.	12 weeks	(Cho et al. 2017)
Intervention group (*n* = 17): participants diagnosed with overweight or obesity.	240 mL of infusion with 15 g of *Murraya paniculata* L. leaves, twice a day, after meals.	NA.	15 days	(Sukohar et al. 2017)
Intervention (*n* = 70) and control (*n* = 70) groups: overweight participants.	1 capsule of 450 mg of *Moringa oleifera* leaf aqueous ethanol extract (60%), *Murraya koenigii* L. leaf aqueous ethanol extract (30%), and *Curcuma longa* L. root extract (10%) before breakfast and dinner; a diet plan (approximately 1800 kcal/day); and walk for 30 min (5 days/week).	1 placebo capsule before breakfast and dinner; a diet plan (approximately 1800 kcal/day); and walk for 30 min (5 days/week).	16 weeks	(Dixit et al. 2018)
Intervention (*n* = 30) and control (*n* = 20) groups: participants diagnosed with obesity.	1 capsule of 250 mg of *Helianthus annuus* seed extract (SUNCA) before breakfast and lunch; and a diet plan with a 500‐kcal deficit.	1 placebo capsule before breakfast and lunch; and a diet plan with a 500‐kcal deficit.	12 weeks	(Leverrier et al. 2019)
Intervention 1 (*n* = 40), intervention 2 (*n* = 40), and control (*n* = 40) groups: participants diagnosed with overweight or obesity.	*Intervention 1* (*Triphala*): 500 mg of *Cyperus rotundus* , *Embelia ribes*, and *Plumbago zeylanica* extracts. *Intervention 2* (*Trimad*): 500 mg of *Terminalia chebula* . *Termenalia bellerica*, and *Phyllanthus emblica* extracts. For both interventions, 2 capsules twice a day for overweight participants and 3 times a day for obese participants.	2 placebo capsules twice a day for overweight participants and 2 placebo capsules 3 times a day for obese participants.	4 months	(Salunke et al. 2019)
Intervention (*n* = 76) and control (*n* = 73) groups: participants diagnosed with obesity.	3 g extract of 1.33 g of *Ephedra sinica* Stapf, 1.33 g of *Angelica gigantis Radix*, 1.33 g of *Atractylodis rhizoma Alba*, 3.33 g of *Coicis Semen*, 1 g of *Cinnamomi cortex*, 1 g of *Paeonia lactiflora* , and 0.67 g of *Glycyrrhiza uralensis* after breakfast, lunch, and dinner.	3 g of placebo extract after breakfast, lunch, and dinner.	12 weeks	(Cheon et al. 2020)
Intervention group (*n* = 18): overweight participants.	2 tablets of Herbal Formulation for Obesity (HFO)‐02, twice a day, before meals. The composition of the tablets was Triphala powder (150 mg‐combination of dried fruits of *Terminalia chebula* , *Emblica officinalis* and *Terminalia belerica*), Trimad powder (60 mg‐combination of tubers of *Cyperus rotundus* , fruits of *Embelia ribes* and roots of *Plumbago zeylanica* ), latex of Guggul (50 mg of *Commiphora mukul*), and Vrikshamla fruit powder (250 mg of *Garcinia cambogia*).	NA.	3 months	(Gupte et al. 2020)
Intervention 1 (*n* = 31), intervention 2 (*n* = 29), and control groups (*n* = 28): participants diagnosed with overweight or obesity.	*Intervention 1*: 1 capsule of 400 mg of *Citrus bergamia* Risso (83.33%) and *Eurycoma longifolia* (16.67%) (CitruSlim‐HD) before breakfast, lunch, and dinner. *Intervention 2*: 1 capsule of 400 mg of *Citrus bergamia* Risso (83.33%) and *Eurycoma longifolia* (16.67%) (CitruSlim‐LD), and 200 mg of methyl crystalline cellulose before breakfast, lunch, and dinner. All participants received a diet plan (20 kcal/kg) and physical activity recommendation.	1 placebo capsule before breakfast, lunch, and dinner; a diet plan (20 kcal/kg), and physical activity recommendation.	112 days	(Hancke et al. 2021)
Intervention (*n* = 20) and control (*n* = 20) groups: participants diagnosed with obesity.	3 to 5 capsules of *Ganoderma lucidum*; *Coptis chinensis*; *Astragalus membranaceus*; *Nelumbo nucifera* Gaertn; and *Fructus aurantii* according to body weight before breakfast, lunch, and dinner. Recommendation of diet and intake of at least 1.5 L of water.	3 to 5 placebo capsules according to body weight before breakfast, lunch, and dinner. Recommendation of diet and intake of at least 1.5 L of water.	2 months	(Cao et al. 2023)

*Note*: The studies conducted by Ignjatovic et al. (2000), Greenway, deJonge‐Levitan, Martin, et al. (2006), Kamali et al. (2012), Zhou et al. (2014), Salunke et al. (2019), Gupte et al. (2020), Liu et al. (2022), and Cao et al. (2023) did not report the doses of herbal medicines used in the intervention.

Abbreviations: g: gram; mcg: microgram; mg: milligram; NA: not applied; NR: not reported.

**TABLE 2:** Description of study groups, herbal medicines, and intervention periods in studies combining herbal medicines with dietary supplements or pharmacotherapy (*n* = 23).

Groups of study (*N*)	Intervention group	Control group	Time period	References
Intervention groups: 1 (*n* = 8): normal weight or overweight participants. 2 (*n* = 14): participants diagnosed with obesity. 3 (*n* = 7): participants diagnosed with type 2 diabetes *mellitus*. The participants maintained the use of tolbutamide (antidiabetic drug).	Decoction of 100 g of broiled nopal leaves (*Opuntia* sp.) before breakfast, lunch, and dinner.	NA.	10 days.	(Frati‐Munari et al. 1983)
Intervention (*n* = 13) and control (*n* = 13) groups: participants diagnosed with obesity.	Days 1 and 2: 1 pill of *Ephedra sinica* Days 3 to 44: 2 pills of the same intervention before breakfast and afternoon meal.	Days 1 and 2: 1 placebo pill before breakfast and afternoon meal. Days 3 to 44: 2 placebo pills before breakfast, and afternoon meal.	44 days	(Armstrong et al. 2001)
Intervention 1 (*n* = 90) and intervention 2 (*n* = 38) groups: participants diagnosed with obesity.	*Intervention 1*: 1 dose of BioLean (amino acids [400 mg of L‐phenylalanine, L‐tyrosine, and L‐carnitine]) and 650 mg of herbal powder (37.5 mg of *Ma Huang*, green tea, *Schisandra chinensis*, *Rehmannia glutinosa* root, *Crataegus* (hawthorn berry), *Ziziphus jujuba* (jujube seed), *Alisma orientale* root, *Angelica dahuricae* root, *Epemidium*, *Poria cocos*, Rhizoma Rhei, *Stephania tetrandra* root, *Angelica sinensis* root, *Codonopsis* root, *Eucommia ulmoides* bark, and *Panax notoginseng* root), 3 times a day before meals. *Intervention 2*: 1 dose of Satiete powder (150 mg of *Griffonia* seed extract, 99 mg of *Gymnema sylvestre* , 1980 mcg of vanadyl sulfate, 19.5 mg of vitamin B2, 19.5 mg of niacinamide, 165 mg of magnesium oxide, 19.5 mg of vitamin B1, 19.5 mg of vitamin B6, 300 mg of malic acid, 150 mg of St. John's wort extract, 60 mg of *Gingko biloba* extract, 300 mcg of vitamin B12 (300 mcg), and 99 mcg of folic acid), 3 times a day before meals.	NA.	Intervention 1: 18.7 weeks (mean) Intervention 2: 12.4 weeks (mean)	(Sindler and Sindler 2001)
Intervention (*n* = 12) and control (*n* = 12) groups: participants diagnosed with obesity.	2 capsules of 697.5 mg of *Gymnema sylvestre* , *Trigonella foenum‐graecum* , glucomannan, chitosan, and vitamin C, 1 h before breakfast, lunch, and dinner.	2 placebo capsules, 1 h before breakfast, lunch, and dinner.	6 weeks	(Woodgate and Conquer 2003)
Intervention (*n* = 12) and control groups—Phase 1 (*n* = 12): participants diagnosed with overweight or obesity. Intervention (*n* = 20) and control (*n* = 20) groups—Phase 2 and 3: participants diagnosed with overweight or obesity.	*Phase 1*: 2 pills of 150 mg of *Ma Huang*, 150 mg of kola nut seed, 100 mg of chromium, 50 mg of green tea (with 5 mg of caffeine), 50 mg of adrenal gland, 50 mg of L‐phenylalanine, 50 mg of ginger root, 50 mg of Fo‐ti root, 50 mg of Lycii berry fruit, 50 mg of *Siberian ginseng* root, 50 mg of cinnamon bark, 5 mg of zinc, 50 mg of vanadium (aspartate), 30 mg of *Astragalus*, 30 mg of caffeine, 20 mg of magnesium, 20 mg of *Ginkgo biloba* , and 10 mg of pyridoxal α‐ketoglutarate after resting, metabolic rate was measured for 30 min of each hour for 2 h. *Phase 2*: 2 pills of 70 mg of caffeine and 24 mg of ephedrine with 3 meals associated with a diet plan (1200 kcal/day for women or 1500 kcal/day for men); and walk for 40 min per day. *Phase 3*: 2 intervention pills with 3 meals.	*Phase 1*: 2 placebo pills. *Phase 2*: 2 placebo pills with 3 meals; a diet plan (1200 kcal/day for women or 1500 kcal/day for men); and walk for 40 min per day. *Phase 3*: 2 intervention pills with 3 meals.	Phase 1: 2 days. Phase 2: 3 months. Phase 3: 3 months (intervention) and 6 months (control).	(Greenway et al. 2004)
Intervention (*n* = 20) and control (*n* = 10) groups: participants diagnosed with overweight or obesity.	All participants received these 3 types of intervention: *Intervention 1*: 3 capsules after breakfast (vitamin C, vitamin B1, vitamin B2, vitamin B3, vitamin B6, vitamin B9, vitamin B12, pantothenic acid, choline, inositol, 4‐aminobenzoic acid, calcium, phosphorus, chromium, vanadium, *Garcinia cambogia*, *Gymnema sylvestre* , white kidney bean extract, and green tea). *Intervention 2*: 3 capsules 15 min before lunch (psyllium (51%), chitosan (40%), glucomannan (4%), and pectin (4%)). *Intervention 3*: 3 capsules in the morning and 1 h at night or 30 min before a light workout (complex B vitamins, conjugated linoleic acid, taurine, lecithin, green tea extract, L‐carnitine, inositol phosphate, vitamin C, ginger extract, capsaicin).	NR.	6 weeks	(González et al. 2004)
Intervention (*n* = 44) and control (*n* = 41) groups: participants diagnosed with obesity with impaired glucose tolerance.	1 pill of 66.7 g of talcum, 44.4 g of *Scutellariae radix*, 44.4 g of *Glycyrrhizae radix*, 44.4 g of *Platycodi radix*, 44.4 g of *Gypsum fibrosum*, 44.4 g of *Atractylodis Rhizoma*, 26.7 g of *Schizonepetae spica*, 26.7 g of *Gardeniae fructus*, 26.7 g of *Paeoniae radix*, 26.7 g of *Cnidium rhizoma*, 26.7 g of *Angelicae radix*, 26.7 g of *Menthae herba*, 26.7 g of *Ledebouriellae radix*, 26.7 g of *Ephedrae herba*, 26.7 g of *Forsythiae fructus*, 33.3 g of *Rhei rhizoma*, 15.6 g of *Natrium sulphuricum* and 6.7 g of *Zingiberis rhizom* before breakfast, lunch, and dinner; a diet plan (1200 kcal/day); and physical activity (5000 steps/day).	1 placebo pill before breakfast, lunch, and dinner; a diet plan (1200 kcal/day); and physical activity (5000 steps/day).	24 weeks	(Hioki et al. 2004)
Intervention 1 (*n* = 10), intervention 2 (*n* = 10), and control (*n* = 10) groups: participants diagnosed with obesity.	*Intervention 1*: 1 pill of 4667 mg of *Garcinia cambogia* 1 h before breakfast, lunch, and dinner. *Intervention 2*: 1 pill of 4667 mg of *Garcinia cambogia*, niacin‐bound chromium (4 mg corresponding to 400 g elemental chromium), and 400 mg of *Gymnema sylvestre* 1 h before breakfast, lunch, and dinner.	1 placebo pill 30 to 60 min before breakfast, lunch, and dinner.	8 weeks	(Preuss et al. 2004)
Intervention (*n* = 29) and control (*n* = 31): participants diagnosed with overweight or obesity.	1 pill of 2000 mg of *Garcinia camboja* extract, 550 mg of guarana extract, 500 mg of *Ephedra* extract, 200 mg of oolong tea extract, 50 mg of garlic extract, 50 mg of tulsi extract, 50 mg of *Eleutherococcus senticosis* extract, 20 mg of green tea extract, 10 mg of *Ginkgo biloba* extract, 10 mg of *Gymnema sylvestre* extract, 5 mg of silymarin extract, and 5 mg of red wine polyphenols 30 to 45 min before breakfast and lunch; 1 pill of vitamins (A, C, D, E, K, B1, B2, B3, B6, B9, and B12), biotin, pantothenic acid, calcium, iron, phosphorus, iodine, magnesium, zinc, selenium, copper, manganese, chromium, molybdenum, chloride, potassium, boron, nickel, silicon, tin, vanadium, lutein, choline, coenzyme Q‐10, L‐glutathione, L‐methionine, bioflavonoids, L‐carnitine, taurine, beta‐sitosterol; and 1 pill of eicosapentaenoic acid and docosahexaenoic acid with lunch or dinner.	1 pill 30 to 45 min before breakfast and lunch; and 2 pills (interventions 2 and 3) with lunch or dinner.	9 months	(Hackman et al. 2006)
Intervention 1 (*n* = 8) and control 1 (*n* = 8) groups: participants diagnosed with overweight or obesity. Intervention 2 (*n* = 20) and control 2 (*n* = 20) groups: participants diagnosed with overweight or obesity.	*Intervention 1*: 2 capsules of 40 mg of pantothenic acid, 200 mg of green tea leaf extract, 550 mg of guarana extract, 150 mg of bitter orange, white 50 mg of willow bark extract, 10 mg of ginger root, and 375 mg of proprietary charge thermoblend (L‐tyrosine, L‐carnitine, and naringin) 2 times a day. *Intervention 2*: 1 capsule of 20 mg of phenylephrine 3 times a day. Diet plan (1200 kcal/day diet for women and a 1500 kcal/day diet for men) and walking recommendations 30 min a day were received.	*Intervention 1*: 2 placebo capsules 2 times a day. *Intervention 2*: 1 placebo capsule 3 times a day. Diet plan (1200 kcal/day diet for women and a 1500 kcal/day diet for men) and walking recommendations 30 min a day were received.	8 weeks	(Greenway, de Jonge‐Levitan, et al. 2006)
Intervention (*n* = 27) and control (*n* = 18) groups: participants diagnosed with overweight or obesity.	2 pills of Prograde Metabolism (1.5 mg of thiamin, 1.7 mg of riboflavin, 20 mg of niacin, 10 mg of vitamin B6 and 200 mcg of B12, 1000 mcg biotin, 10 mg of pantothenic acid, 200 mcg of chromium and a blend of 1000 mg with raspberry ketone, caffeine anhydrous, bitter orange, ginger root extract, garlic root extract, cayenne extract, L‐theanine and *Piper nigrum* ) with breakfast and lunch; a diet plan with a 500‐kcal deficit; and physical activity program.	2 placebo pills with breakfast and lunch; a diet plan with a 500‐kcal deficit; and a physical activity program.	8 weeks	(Lopez et al. 2013)
Intervention (*n* = 24) and control (*n* = 26) groups: overweight participants with pre‐diabetes.	Glucaffect powder with 15 g of French maritime pine bark extract, 8.78 g of low‐fat soy flour, 500 mg of *Syzygium cumini* , 400 mg of inulin, 250 mg of *Pterocarpus marsupium* , 150 mg of soy lecithin, 120 mg of *Salacia oblonga*, 120 mg of guar and xanthan gum each one, 90 mg of acesulfame potassium, 44 mg of ground cinnamon, 4 mg of *Lagerstroemia speciosa* , 750 mg of Omega‐3 fish oils, 100 mg of alpha lipoic acid, 20 mg of coenzyme Q‐10, and 12.5 mg of L‐glutathione into 200 mL of water, 4 times a day.	Placebo powder into 200 mL of water 4 times a day.	8 weeks	(Belcaro et al. 2009)
Intervention 1 (*n* = 20), intervention 2 (*n* = 20), intervention 3 (*n* = 20), and control (*n* = 20) groups: participants diagnosed with obesity and polycystic ovary syndrome. All groups received 2 pills of 500 mg of metformin pills a day.	*Intervention 1*: granulated of *Foeniculum vulgare* , *Urtica dioica* , *Daucus carota* , *Trifolium pratense* , and *Curcuma longa* (5 g/day) in a sachet. *Intervention 2*: 20 electroacupuncture sessions. *Intervention 3*: granulated of *Foeniculum vulgare* , *Urtica dioica* , *Daucus carota* , *Trifolium pratense* , and *Curcuma longa* (5 g/day) in a sachet and 20 electroacupuncture sessions.	2 pills of 500 mg of metformin pills a day.	12 weeks	(Rouhani et al. 2019)
Intervention group (*n* = 55): overweight participants. 13 participants were taking estrogen replacement therapy, 12 non‐steroidal anti‐inflammatory drugs (musculoskeletal pain and headaches), and 7 oral antibiotics (upper respiratory, urinary, and skin infections).	1 pill of 500 mg of hydroxycitric acid (*Garcinia cambogia*) and 100 mcg of chromium picolinate, 30 min before breakfast, lunch, and dinner.	NA.	8 weeks	(Badmaev et al. 2001)
Intervention 1 (*n* = 25) and intervention 2 (*n* = 25) groups: participants diagnosed with type 2 diabetes *mellitus*. Participants maintained the use of antidiabetic drugs.	340 mL of *Camellia sinensis* (583 mg of catechins) and 72.3 mg of caffeine, 1 h before dinner.	340 mL of *Camellia sinensis* (96 mg of catechins) and 75 mg of caffeine, 1 h before dinner.	12 weeks	(Nagao et al. 2009)
Intervention (*n* = 93) and control (*n* = 93) groups: participants diagnosed with overweight or obesity. Participants maintained the use of oral contraceptives, antihypertensive and lipid‐lowering drugs.	2 capsules of 25 mg of quercetin dehydrate at breakfast, lunch, and dinner.	2 placebo capsules at breakfast, lunch, and dinner.	6 weeks and 5 weeks of washout	(Egert et al. 2009)
Intervention (*n* = 29) and control (*n* = 25) groups: obese or overweight participants diagnosed with cancer. All received chemotherapy and 56.4% received it in combination with radiotherapy.	240 mL of *Camellia sinensis* tea (550 to 700 mg of extract and 58.91 mg of catechin per bag), 4 times a day.	240 mL of placebo tea, 4 times a day.	6 months	(Stendell‐Hollis et al. 2010)
Intervention (*n* = 80) and control (NR) groups: participants diagnosed with overweight or obesity. About half of the participants were in use of antihypertensive and antidiabetic drugs and pharmacotherapy for the management of ischemic heart disease.	1 pill of 60 mg of *Alchemilla vulgaris* L., 50 mg of *Olea europaea* L., 20 mg of *Mentha longiforia* L., 25 mg of *Cuminum cyminum* L., 7 mg of vitamin C, 148 mg of and tricalcium phosphate 30 min before each meal. Participants restricted to 3 meals per day.	Participants restricted to 3 meals per day.	3 months	(Said et al. 2011)
Intervention (*n* = 25) and control (*n* = 25) groups: obese or overweight participants with waist circumference > 85 cm.	1 capsule of *Bofutsushosan*, *Streptococcus thermophiles*, *Lactobacillus plantarum* , *Lactobacillus acidophilus* , *Lactobacillus rhamnosus* , *Bifidobacterium lactis* , *Bifidobacterium longum* , and *Bifidobacterium breve* , 2 times a day; a diet with a caloric intake limited to 20–25 kcal/kg; and physical activity program.	1 capsule of *Bofutsushosan* and 1 capsule of placebo, 2 times a day; a diet with a caloric intake limited to 20–25 kcal/kg; and physical activity program.	8 weeks	(Lee et al. 2014)
Intervention (*n* = 48) and control (*n* = 50) groups: participants diagnosed with hyperlipidemia.	2 capsules of 500 mg of *Citrus bergamia* Risso extract, 820 mg of plant sterol esters and orange oil, 50 mg of vitamin C, 20 mg of vitamin B6, 2000 mcg of vitamin B12 and 800 mcg of folic acid, with meals, 2 times a day.	2 placebo capsules with meals, 2 times a day.	12 weeks	(Cai et al. 2017)
Intervention group (*n* = 157): participants diagnosed with overweight or obesity. 38 participants were in use of antihypertensive, lipid‐lowering, and antidiabetic drugs.	1 pill of 6 mg of *Citrus unshiu* dried peels 3 times a day.	NA.	4 weeks	(Kang et al. 2018)
Intervention (*n* = 31) and control (*n* = 31) groups: participants diagnosed with metabolic syndrome and obesity. Participants maintained the use of metformin and statins.	1 pill of *Folium nelumbinis*, *Radix Salviae miltiorrhizae*, *Fructus* c*rataegi*, *Folium sennae*, and *Fructus* p*soraleae*, 3 times a day.	1 placebo pill, 3 times a day.	24 weeks	(Liu et al. 2022)
Intervention 1 (*n* = 8) and intervention 2 (*n* = 8) group: NR.	*Intervention 1*: 1 dose of TrimRox powder (6.75 g of *Gynostemma pentaphyllum*; *Ilex guayusa*; *Camellia sinensis* , *Myrciaria dúbi*, D‐ribose nicotinamide complex, and L‐α‐Glycerophosphorylcholine) in the morning, on an empty stomach *Intervention 2*: 2 doses of TrimRox powder in the morning, on an empty stomach.	NA.	21 days	(Bagchi et al. 2022)

*Note*: The studies conducted by Sindler and Sindler (2001), González et al. (2004), Hackman et al. (2006), Rouhani et al. (2019), and Bagchi et al. (2022) did not report the doses of herbal medicines evaluated.

Abbreviations: g: gram; mcg: microgram; mg: milligram; NA: not applied; NR: not reported.

### 3.4 Use of Herbal Medicine Products and Their Effects on Anthropometric Parameters

Out of the 74 included studies, 10 reported no benefits from the use of herbal medicine products on anthropometric parameters (Bell et al. 2011; Greenway, Liu, et al. 2006; Lee et al. 2014; Stendell‐Hollis et al. 2010; Astrup et al. 1992; Chan et al. 2006; Henderson et al. 2005; Hu et al. 2012; Kovacs et al. 2001; Park et al. 2013), with the majority conducted in the United States (*n* = 5). Among these, 2 did not combine herbal medicinal products with a diet plan (Henderson et al. 2005; Kovacs et al. 2001) and adherence to the intervention was not reported in 4 studies (Lee et al. 2014; Astrup et al. 1992; Chan et al. 2006; Henderson et al. 2005).

#### 3.4.1 Weight Loss

Improvements in body fat mass (kg), body weight (kg), BMI (kg/m^2^), and hip and waist circumference (cm) were observed across different herbal medicine interventions. Twenty‐six studies demonstrated statistically significant improvements across all anthropometric parameters assessed (Dixit et al. 2018; Kamohara and Noparatanawong 2013; Landor et al. 2015; Armstrong et al. 2001; Badmaev et al. 2001; Belcaro et al. 2009; Boozer et al. 2001, 2002; González et al. 2004; Preuss et al. 2004; Greenway et al. 2004; Hioki et al. 2004; Kazemipoor et al. 2013; Sengupta et al. 2012; Stern, Peerson, Mishra, Sadasiva Rao, and Rajeswari 2013; Stern, Peerson, Mishra, Mathukumalli, and Konda 2013; Tripathy et al. 2013; Di Pierro et al. 2009; Said et al. 2011; Dellalibera et al. 2006; Frati‐Munari et al. 1983; Zhou et al. 2014; Liu et al. 2022; Kamali et al. 2012; Rouhani et al. 2019; Heymsfield et al. 1998). Three studies reported improvements, but did not provide statistical data (Bagchi et al. 2022; Sindler and Sindler 2001; Andersen and Fogh 2001).

Significant reductions in body weight were observed in 50 out of 67 studies. The most significant mean reductions were: 13.8 kg after using *Camellia sinensis* tablets for 3 months (Di Pierro et al. 2009); 12.0 kg after using tablets containing *Cuminum cyminum* L., *Mentha longifolia* L., *Olea europaea* L., and *Alchemilla vulgaris* L. for 3 months (Said et al. 2011); and 10.8 kg (SD ± 10.3) after using a herbal medicine product containing *Scutellariae radix*, *Glycyrrhizae radix*, *Platycodi radix*, *Gypsum fibrosum*, *Atractylodis rhizoma*, *Schizonepetae spica*, *Gardeniae fructus*, *Paeoniae radix*, *Cnidium rhizoma*, *Angelicae radix*, *Menthae herba*, *Ledebouriellae radix*, *Ephedrae herba*, *Forsythiae fructus*, *Rhizoma Rhei*, *Natrium sulphuricum*, and *Zingiberis rhizoma*, 1200 kcal/day diet plan and physical activity (5000 steps/day) (Hioki et al. 2004).

Of the 53 studies that evaluated BMI, reductions were observed in 41. Among the most pronounced BMI reductions were 5.37 kg/m^2^ after using *Garcinia cambogia* caplets for 4 months (Tripathy et al. 2013); 4.0 kg/m^2^ after using tablets containing *Cuminum cyminum* L., *Mentha longifolia* L., *Olea europaea* L., and *Alchemilla vulgaris* L. for 3 months (Said et al. 2011); and 4.0 kg/m^2^ after using the herbal medicine product previously described, combined with a diet plan and physical activity for 3 months (Hioki et al. 2004).

A decrease in waist circumference was reported in 29 of the 45 studies, with mean reductions reaching 12.5 cm (SD ± 1.83) after using *Moringa oleifera* , *Murrya koenigi*, and *Curcuma longa* after 8 weeks (Sengupta et al. 2012); 8.0 cm after using drops of *Hordeum vulgare* L., *Polygonatum multiflorum* (L.) All., *Dimocarpus longan* Lour., *Ligusticum sinense* Oliv., *Lilium brownii* F.R. Br. ex Miellez, *Gynura pinnatifida*, *Coreopsis lanceolata* , *Juniperus communis* , and *Zingiber officinale* Roscoe after 6 weeks (Ignjatovic et al. 2000); and 6.2 cm after using *Carum carvi* L. combined with a diet plan and physical activity (180 min/week) for 3 months (Kazemipoor et al. 2013).

Body fat mass was evaluated in 36 studies, with improvements observed in 20. The most significant mean loss of body fat mass was 3.9 kg (SD ± 5.1) in participants diagnosed with overweight after using green tea extract (Diepvens et al. 2006), followed by 2.9 kg in participants with overweight or obesity after using Prograde Metabolism^TM^, combined with a diet plan providing a 500‐kcal deficit and physical activity program for 8 weeks (Kamohara and Noparatanawong 2013).

Reductions in hip circumference were noted in 20 out of the 32 studies that assessed this parameter. Studies reported reductions of up to 9.9 cm (SD ± 1.34) in hip circumference after using *Moringa oleifera* , *Murraya koenigii*, and *Curcuma longa* for 8 weeks (Sengupta et al. 2012); 8.0 cm after using drops of *Hordeum vulgare* L., *Polygonatum multiflorum* (L.) All., *Dimocarpus longan* Lour., *Ligusticum sinense* Oliv., *Lilium brownii* F.R. Br. ex Miellez, *Gynura pinnatifida*, *Coreopsis lanceolata* , *Juniperus communis* , and *Zingiber officinale* Roscoe for 6 weeks (Ignjatovic et al. 2000); and 6.0 cm after using *Ma Huang* and Kola for 6 months (Boozer et al. 2002).

#### 3.4.2 Weight Gain

One study reported weight gain, which was accompanied by increases in body fat mass and waist circumference (Greenway, de Jonge‐Levitan, et al. 2006). Participants in this study received capsules containing *Camellia sinensis* , guarana extract, bitter orange, white willow bark extract, ginger root, pantothenic acid, L‐tyrosine, L‐carnitine, and naringin, combined with moderate‐intensity physical activity and a calorie‐restricted diet for 8 weeks (Greenway, de Jonge‐Levitan, et al. 2006). The authors attributed the observed weight gain to randomness, citing the low statistical power of the sample, which included only 20 participants.

Tables 3 and 4 detail the anthropometric outcomes associated with the use of herbal medicines included in this review.

**TABLE 3:** Weight loss and improvement of anthropometric parameters following interventions with herbal medicines, herbal medicines combined with dietary supplements, or herbal medicines in combination with pharmacotherapy, as reported in the studies included in this scoping review (*n* = 64).

Intervention	Improved anthropometric parameter	References
**Herbal medicine**		
*Aster spathulifolius* Maxim	BFM, BMI, body composition, and WEI.	(Cho et al. 2016)
*Camellia sinensis* L. (extract)	BFM, BMI, WC, WEI, and WHR.	(Diepvens et al. 2006)
	HC and WC.	(Hsu et al. 2008)
*Camellia sinensis* L.	BFM, BMI, HC, subcutaneous fat area, total fat area, visceral fat area, WC, and WEI	(Nagao et al. 2007)
	WEI.	(Auvichayapat et al. 2008)
	BMI, WC, and WEI.	(Di Pierro et al. 2009)
*Camellia sinensis* and caffeine	WC and WHR.	(Nagao et al. 2009)
*Camellia sinensis* , *Cassia obtusifolia* , and *Sophora japonica*	BFM, BMI., HC, and WEI.	(Lenon et al. 2012)
*Carum carvi* L.	BFM, BMI, body composition, HC, WC, WEI, and WHR.	(Kazemipoor et al. 2013)
*Citrus unshiu*	BMI, WC, and WEI.	(Kang et al. 2018)
*Citrus aurantium* , rhubarb, coptis, and *Semen cassia*	BMI, HC, WC, and WEI.	(Zhou et al. 2014)
*Citrus bergamia* Risso and *Eurycoma longifolia*	BMI.	(Hancke et al. 2021)
*Coleus forskohlii*	BFM, BMI, lean body mass, and WEI.	(Kamohara and Noparatanawong 2013)
*Cyperus rotundus* , *Embelia ribes*, and *Plumbago zeylanica* (intervention 1) (Triphala) *Terminalia chebula* , *Termenalia bellerica*, and *Phyllanthus emblica* , *Commiphora mukul*, and *Garcinia cambogia* (intervention 2) (Trimad)	Arm circumference, HC, thigh circumference, and WC	(Salunke et al. 2019)
*Ephedra sinica* and *Evodia rutaecarpa*	BMI.	(Kim et al. 2008)
*Ephedra sinica* and *Evodia rutaecarpa*	BFM, BMI, and WEI.	(Kim et al. 2014)
*Ephedra sinica* Stapf, *Angelica gigantis* Radix, *Atractylodis* rhizoma *Alba*, *Coicis semen*, *Cinnamomi cortex*, *Paeonia lactiflora* , and *Glycyrrhiza uralensis*	BMI, HC, WC, and WEI.	(Cheon et al. 2020)
*Folium nelumbinis*, *Folium sennae*, *Radix salviae Miltiorrhizae*, *Fructus crataegi*, and *Fructus* p*soraleae*	BMI and WEI.	(Liu et al. 2022)
*Ganoderma lucidum*, *Coptis chinensis*, *Astragalus membranaceus*, *Nelumbo nucifera* Gaertn., and *Fructus aurantii*	WEI.	(Cao et al. 2023)
*Garcinia cambogia*	WEI.	(Badmaev et al. 2001)
	BMI and mid axillary, subscapular, and thigh circumference.	(Tripathy et al. 2013)
	BFM and WEI.	(Heymsfield et al. 1998)
*Garcinia cambogia*, *Camellia sinensis* , *Coffea arábica*, and *Lagerstroemia speciosa*	BFM, BMI, HC, WC, and WEI.	(Chong et al. 2014)
*Glycyrrhiza glabra*	BFM (300 mg, 600 mg, and 900 mg), BMI (900 mg), lean body mass (300 mg and 600 mg), visceral fat area (900 mg), and WEI (900 mg).	(Tominaga et al. 2009)
	WEI.	(Mirtaheri et al. 2015)
*Gynostemma pentaphyllum*	BFM, BMI, and WEI.	(Park et al. 2014)
Guarana and *Ma Huang*	BFM, HC, WC, and WEI.	(Boozer et al. 2001)
Green coffee	BMI and WEI.	(Dellalibera et al. 2006)
*Helianthus annuus*	BMI and WC.	(Leverrier et al. 2019)
*Hordeum vulgare* L., *Polygonatum multiflorum* (L.) All., *Dimocarpus longan* Lour., *Ligusticum sinense* Oliv., *Lilium brownii* F.R. Br. ex Miellez, *Gynura pinnatifida*, *Coreopsis lanceolata* , *Juniperus communis* , and *Zingiber officinale* Roscoe	BMI, HC, WC, and WEI.	(Ignjatovic et al. 2000)
*Hoodia gordonii*	WEI.	(Blom et al. 2011)
*Hoodia parviflora*	BMI, WHR, and WEI.	(Landor et al. 2015)
*Ilex paraguariensis*	WHR.	(Kim et al. 2015)
*Ilex paraguayensis* , *Paullinia cupana* , and *Turnera diffusa var* . *aphrodisiaca*	WEI.	(Andersen and Fogh 2001)
*Imperata cylindrica* Beauvois, *Citrus unshiu* Markovich, and *Evodia officinalis*	BFM, BMI, WC, and WEI.	(Cho et al. 2017)
*Ma Huang* and Kola nut	BFM, HC, WC, and WEI.	(Boozer et al. 2002)
*Ma Huang*, Kola nut, and White willow bark	BMI, WC, and WEI.	(Coffey et al. 2004)
*Moringa olefera*, *Murraya koenigii* , and *Curcuma longa*	BMI, HC, WC, WEI, and WHR.	(Sengupta et al. 2012)
*Moringa oleifera* , *Murraya koenigii* (L.), and *Curcuma longa* L.	BMI, HC, WC, WEI, and WHR.	(Dixit et al. 2018)
*Murraya paniculata* L.	BMI.	(Sukohar et al. 2017)
Nopal (*Opuntia* sp.)	WEI—only in participants diagnosed with obesity and type 2 diabetes *mellitus*.	(Frati‐Munari et al. 1983)
*Puerariae thomsonii*	BMI (300 mg), subcutaneous fat area (200 mg and 300 mg), total fat area (200 mg and 300 mg), WC (300 mg), and WEI (300 mg).	(Kamiya et al. 2011)
*Phyllanthus emblica* L. *Terminalia chebula* Retz., and *Terminalia belerica* Retz	BMI, HC, WC, and WEI.	(Kamali et al. 2012)
Quercetin	BFM.	(Egert et al. 2009)
*Sphaeranthus indicus* and *Garcinia mangostana*	BMI, HC, WC, WEI, and WHR.	(Stern, Peerson, Mishra, Sadasiva Rao, and Rajeswari 2013)
*Scutellariae radix* and *Platycodi radix*	WEI.	(Cho et al. 2013)
*Sphaeranthus indicus* and *Garcinia mangostana*	BMI, HC, and WEI.	(Stern, Peerson, Mishra, Mathukumalli, and Konda 2013)
Triphala powder (dried fruits of *Terminalia chebula* , *Emblica officinalis* and *Terminalia belerica*), Trimad powder (tubers of *Cyperus rotundus* , fruits of *Embelia ribes* and roots of *Plumbago zeylanica* ), latex of Guggul (*Commiphora mukul*), and Vrikshamla fruit powder (*Garcinia cambogia*).	HC, neck circumference, and WC.	(Gupte et al. 2020)
Herbal medicine associated with dietary supplement		
*Alchemilla vulgaris* L., *Olea europaea* L., *Mentha longiforia* L., *Cuminum cyminum* L., vitamin C, and tricalcium phosphate	BMI and WEI.	(Said et al. 2011)
*Angelica sinensis* root, green tea, *Schizandrae* berry, *Rehmannia* root, hawthorn berry, jujube seed, alisma root, *Angelica dahuricae* root, *Epemidium*, *Poria cocos*, Rhizoma Rhei, *Stephania tetranda* root, *Codonopsis pilosula* root, *Eucommia ulmoides* (eucommium bark), *Panax notoginseng* root, L‐phenylalanine, L‐tyrosine, and L‐carnitine (BioLean) (intervention 1) *Griffonia* seed extract, *Gymnema sylvestre* , *Gingko biloba* extract, St. John's wort extract, vanadyl sulfate, vitamin B2, niacinamide, magnesium oxide, vitamin B1, vitamin B6, malic acid, vitamin B12, and folic acid (Satiete) (intervention 2)	WEI.	(Sindler and Sindler 2001)
*Citrus bergamia* Risso extract, plant sterol esters and orange oil, folic acid, vitamins C, B6, and B12	BMI, WC, WEI, and WHR.	(Cai et al. 2017)
*Garcinia cambogia*, *Gymnema sylvestre* , white kidney bean extract, green tea, vitamins (C, B1, B2, B3, B6, B9, and B12), panthotenic acid, choline, inositol, 4‐aminobenzoic acid, calcium, phosphorus, chromium, and vanadium (intervention 1) Psyllium, chitosan, glucomannan, and pectin (intervention 2) Ginger extract, green tea extract, complex B vitamins, conjugated linoleic acid, taurine, lecithin, L‐carnitine, inositol phosphate, vitamin C, and capsaicin (intervention 3)	BFM, HC, WC, and WEI.	(González et al. 2004)
*Garcinia cambogia* (intervention 1) *Garcinia cambogia*, *Gymnema sylvestre* , and niacin‐bound chromium (intervention 2)	BMI and WEI.	(Preuss et al. 2004)
*Garcinia camboja* extract, *Ginkgo biloba* extract, *Gymnema sylvestre* extract, guarana extract and ephedra extract, oolong tea extract, garlic extract, tulsi extract, *Eleutherococcus senticosis* extract, green tea extract, silymarin extract, and red wine polyphenols (pill 1) Vitamins (A, C, D, E, K, B1, B2, B3, B6, B9, and B12), biotin, pantothenic acid, calcium, iron, phosphorus, iodine, magnesium, zinc, selenium, copper, manganese, chromium, molybdenum, chloride, potassium, boron, nickel, silicon, tin, vanadium, lutein, choline, coenzyme Q‐10, L‐glutathione, L‐methionine, bioflavonoids, L‐carnitine, taurine, and beta‐sitosterol (pill 2) Eicosapentaenoic acid and docosahexaenoic acid (pill 3) All the participants received 3 intervention pills	BMI and WEI.	(Hackman et al. 2006)
Green tea leaf extract, guarana extract, bitter orange, White willow bark extract ginger root, proprietary charge thermoblend (L‐tyrosine, L‐carnitine, and naringin), and pantothenic acid (intervention 1) Phenylephrine (intervention 2)	WEI.	(Greenway, de Jonge‐Levitan, et al. 2006)
Guarana extract, *Ma Huang*, L‐tyrosine, acetyl L‐carnitine, fisetin, magnesium phosphate, White willow bark extract, bitter orange, ginger root, and vitamin A	BFM and fat‐free mass	(Armstrong et al. 2001)
*Gymnema sylvestre* , *Trigonella foenum‐graecum* , glucomannan, chitosan, and vitamin C	Abdominal circumference, BFM, BMI, HC, WC, and WEI.	(Woodgate and Conquer 2003)
*Gynostemma pentaphyllum*, *Ilex guayusa*, *Camellia sinensis* , *Myrciaria dubia*, D‐ribose nicotinamide complex, and L‐α‐Glycerophosphorylcholine (TrimRox)	HC, upper arms/biceps and chest circumference, WC, and WEI.	(Bagchi et al. 2022)
*Ma Huang*, kola nut seed, chromium, green tea, adrenal gland, L‐phenylalanine, ginger root, fo‐ti root, lycii berry fruit, *Siberian ginseng* root, cinnamon bark, zinc, vanadium (aspartate), *Astragalus*, caffeine, magnesium, *Ginkgo biloba* , and pyridoxal α‐ketoglutarate (phase 1) Caffeine and ephedrine (phase 2)	Lean body mass and WEI in both phases.	(Greenway et al. 2004)
*Piper nigrum* , bitter orange, ginger root extract, garlic root extract, blend with raspberry ketone, caffeine anhydrous, cayenne extract and L‐theanine thiamin, riboflavin, niacin, vitamin B6 and B12, biotin, pantothenic acid, and chromium (Prograde Metabolism)	BFM, HC, WC, and WEI.	(Lopez et al. 2013)
Raspberry (intervention 1) L‐carnitine (intervention 2)	BMI and WC.	(Khazaal et al. 2015)
*Scutellariae radix*, *Glycyrrhizae radix*, *Platycodi radix*, *Atractylodis rhizoma*, *Rhei rhizoma*, *Schizonepetae spica*, *Gardeniae fructus*, *Paeoniae radix*, *Cnidium rhizoma*, *Angelicae radix*, *Menthae herba*, *Ledebouriellae radix*, *Ephedrae herba*, *Forsythiae fructus*, *Zingiberis rhizoma*, Gypsum fibrosum, Talcum, and Natrium sulphuricum	Abdominal subcutaneous fat, abdominal visceral fat, BFM, BMI, and WEI.	(Hioki et al. 2004)
*Syzygium cumini* , inulin, *Pterocarpus marsupium* , *Lagerstroemia speciosa* , soy lecithin, *Salacia oblonga*, guar and xanthan gum, acesulfame potassium, ground cinnamon, omega‐3 fish oils, alpha lipoic acid, coenzyme Q‐10, L‐glutathione, French maritime pine bark extract and low‐fat soy flour (Glucaffect)	BMI and WE.	(Belcaro et al. 2009)
Herbal medicine associated with metformin and electroacupuncture sessions		
*Foeniculum vulgare* , *Urtica dioica* , *Daucus carota* , *Trifolium pratense* , and *Curcuma longa*	BFM and BMI.	(Rouhani et al. 2019)

Abbreviations: BFM: body fat mass; BMI: body mass index; HC: hip circumference; WC: waist circumference; WEI: weight; WHR: waist‐hip ratio.

**TABLE 4:** Anthropometric parameters showing no significant improvement or an increase following interventions in the studies included in this scoping review (*n* = 45)^a^.

Intervention	Anthropometric parameter	References
Anthropometric parameter without change		
*Aster spathulifolius* Maxim	WC.	(Cho et al. 2016)
*Bofutsushosan*, *Streptococcus thermophiles*, *Lactobacillus plantarum* , *Lactobacillus acidophilus* , *Lactobacillus rhamnosus* , *Bifidobacterium lactis* , *Bifidobacterium longum* , and *Bifidobacterium breve*	BMI, fat percentage, fat mass, WEI, and WC.	(Lee et al. 2014)
Caffeine and ephedrine	BFM and WEI.	(Astrup et al. 1992)
*Camellia sinensis* L.	BFM, BMI, HC, WC, and WHR.	(Auvichayapat et al. 2008)
	BFM, BMI, triceps skin fold, WEI, and WHR.	(Chan et al. 2006)
	BFM, BMI, HC, lean body mass, WC, WEI, and WHR.	(Stendell‐Hollis et al. 2010)
*Camellia sinensis* L. (extract)	BMI and WEI.	(Hsu et al. 2008)
	BFM.	(Diepvens et al. 2006)
*Camellia sinensis* and caffeine	BFM, BMI, HC, and WEI.	(Nagao et al. 2009)
*Camellia sinensis* , *Cassia obtusifolia* , and *Sophora japonica*	HC.	(Lenon et al. 2012)
*Citrus bergamia* Risso extract, plant sterol esters and orange oil, folic acid, and vitamins C, B6, and B12	HC.	(Cai et al. 2017)
*Citrus bergamia* Risso and *Eurycoma longifolia*	BFM, fat free mass, and lean body mass.	(Hancke et al. 2021)
*Citrus unshiu*	BFM and fat free mass	(Kang et al. 2018)
*Coicis semen*, *Castaneae semen*, *Raphanus sativus* L., *Schisandra chinensis* Baill, Liriopis tuber, Ephedra, *Platycodon grandiflorus* , and *Acori tatarinowii rhizoma*	BMI, HC, WC, WEI, and WHR.	(Park et al. 2013)
*Coleus forskohlii*	BFM, lean body mass, and WEI.	(Henderson et al. 2005)
*Coptis chinensis*	BFM, BMI, WEI, and WHR.	(Hu et al. 2012)
*Cyperus rotundus* , *Embelia ribes*, and *Plumbago zeylanica* (intervention 1) (Triphala) *Terminalia chebula* , *Termenalia bellerica*, and *Phyllanthus emblica* , *Commiphora mukul*, and *Garcinia cambogia* (intervention 2) (Trimad)	Neck circumference and WHR.	(Salunke et al. 2019)
*Ephedra sinica* and *Evodia rutaecarpa*	BFM and WHR.	(Kim et al. 2008)
	WC.	(Kim et al. 2014)
*Ephedra sinica* Stapf, *Angelica gigantis* Radix, *Atractylodis rhizoma Alba*, *Coicis Semen*, *Cinnamomi cortex*, *Paeonia lactiflora* , and *Glycyrrhiza uralensis*	WHR.	(Cheon et al. 2020)
*Ganoderma lucidum*; *Coptis chinensis*; *Astragalus Membranaceus*; *Nelumbo nucifera* Gaertn.; and *Fructus aurantii*	HC and WC.	(Cao et al. 2023)
*Garcinia cambogia*	WEI.	(Kovacs et al. 2001)
*Garcinia cambogia*, *Camellia sinensis* , *Coffea arábica*, and *Lagerstroemia speciosa*	Fat free mass.	(Chong et al. 2014)
Pill 1: *Garcinia camboja* extract, *Ginkgo biloba* extract, *Gymnema sylvestre* extract, guarana extract and ephedra extract, oolong tea extract, garlic extract, Tulsi extract, *Eleutherococcus senticosis* extract, green tea extract, silymarin extract, and red wine polyphenol. Pill 2: vitamins (A, C, D, E, K, B1, B2, B3, B6, B9, and B12), biotin, pantothenic acid, calcium, iron, phosphorus, iodine, magnesium, zinc, selenium, copper, manganese, chromium, molybdenum, chloride, potassium, boron, nickel, silicon, tin, vanadium, lutein, choline, coenzyme Q‐10, L‐glutathione, L‐methionine, bioflavonoids, L‐carnitine, taurine, and beta‐sitosterol. Pill 3: Eicosapentaenoic acid and docosahexaenoic acid (pill 3). All the participants received 3 intervention pills.	BFM and fat free mass.	(Hackman et al. 2006)
*Glycyrrhiza glabra* L.	BMI, fat free mass, skinfold thickness, WEI, and WHR.	(Bell et al. 2011)
	BMI (300 mg and 600 mg), lean body mass (900 mg), subcutaneous fat area (300 mg, 600 mg, and 900 mg), visceral fat area (300 mg and 600 mg), and WEI (300 mg and 600 mg).	(Tominaga et al. 2009)
	BMI.	(Mirtaheri et al. 2015)
*Gymnema sylvestre* , *Trigonella foenum‐graecum* , glucomannan, chitosan, and vitamin C	Lean body fat.	(Woodgate and Conquer 2003)
*Gynostemma pentaphyllum*	HC, WC, and WHR.	(Park et al. 2014)
*Helianthus annuus*	WEI.	(Leverrier et al. 2019)
*Hordeum vulgare* L., *Polygonatum multiflorum* (L.) All., *Dimocarpus longan* Lour., *Ligusticum sinense* Oliv., *Lilium brownii* F.R. Br. ex Miellez, *Gynura pinnatifida*, *Coreopsis lanceolata* , *Juniperus communis* , and *Zingiber officinale* Roscoe	BFM and HC.	(Ignjatovic et al. 2000)
*Hoodia gordonii*	BFM.	(Blom et al. 2011)
*Ilex paraguariensis*	Arm circumference, HC, thigh circumference, WC, and WEI.	(Kim et al. 2015)
*Imperata cylindrica* Beauvois, *Citrus unshiu* Markovich, and *Evodia officinalis*	Lean body mass.	(Cho et al. 2017)
*Ma Huang*, kola nut, and white willow bark	BFM.	(Coffey et al. 2004)
*Murraya paniculata* L.	HC and WC.	(Sukohar et al. 2017)
*Puerariae thomsonii*	BMI (100 mg and 200 mg), HC (100 mg, 200 mg, and 300 mg), subcutaneous and visceral fat area (100 mg), total fat area, WC (100 mg and 200 mg), and WEI (100 mg and 22 mg).	(Kamiya et al. 2011)
Quercetin	WC and WEI.	(Egert et al. 2009)
Raspberry (intervention 1) L‐carnitine (intervention 2)	WEI.	(Khazaal et al. 2015)
Rhubarb root and stem (Radix et Rhizoma Rhei), astragulus root (*Radix astragali*), red sage root (*Radix salviae miltiorrhizae*), turmeric (rhizoma *Curcumae longae*), and ginger rhizome (*Zingiberis officinalis*)	WEI.	(Greenway, Liu, et al. 2006)
*Scutellariae Radix* and *Platycodi Radix*	BMI, body composition, HC, WC, and WHC.	(Cho et al. 2013)
Triphala powder (dried fruits of *Terminalia chebula* , *Emblica officinalis* and *Terminalia belerica*), Trimad powder (tubers of *Cyperus rotundus* , fruits of *Embelia ribes* and roots of *Plumbago zeylanica* ), latex of Guggul (*Commiphora mukul*), and Vrikshamla fruit powder (*Garcinia cambogia*).	BMI, mid‐thigh circumference, mid upper arm circumference, and WEI.	(Gupte et al. 2020)
Increase in anthropometric parameter		
*Camellia sinensis* L.	Lean body mass.	(Nagao et al. 2007)
Quercetin	Fat free mass.	(Egert et al. 2009)
*Glycyrrhiza glabra* L.	BFM, HC, and WC.	(Bell et al. 2011)
Green tea leaf extract, guarana extract, bitter orange, white willow bark extract ginger root, proprietary charge thermoblend (L‐tyrosine, L‐carnitine, and naringin) and pantothenic acid (intervention 1) Phenylephrine (intervention 2)	BFM, WC, and WEI.	(Greenway, de Jonge‐Levitan, et al. 2006)
*Piper nigrum* , bitter orange, ginger root extract, garlic root extract, blend with raspberry ketone, caffeine anhydrous, cayenne extract and L‐theanine, riboflavin, niacin, vitamin B6 and B12, biotin, pantothenic acid, and chromium (Prograde Metabolism)	Lean body fat.	(Lopez et al. 2013)

Abbreviations: BFM: body fat mass; BMI: body mass index; HC: hip circumference; NR: not reported; WC: waist circumference; WEI: weight; WHR: waist‐hip ratio.

^a^ The parameters presented showed no significant improvement or an increase following the intervention. However, these findings do not imply the absence of improvement in other parameters that were assessed in these studies.

### 3.5 Use of Herbal Medicines Products and Their Effects on Biochemical Parameters

The primary biochemical outcomes assessed included total cholesterol, triglycerides, low‐density lipoprotein (LDL), and high‐density lipoprotein (HDL) levels. Of the 74 studies included, 29 evaluated at least one biochemical parameter, and all these reported significant improvements following interventions with herbal medicine products (Cao et al. 2023; Dixit et al. 2018; Leverrier et al. 2019; Badmaev et al. 2001; Boozer et al. 2001, 2002; González et al. 2004; Hackman et al. 2006; Preuss et al. 2004; Hioki et al. 2004; Sengupta et al. 2012; Stendell‐Hollis et al. 2010; Stern, Peerson, Mishra, Sadasiva Rao, and Rajeswari 2013; Stern, Peerson, Mishra, Mathukumalli, and Konda 2013; Astrup et al. 1992; Kang et al. 2018; Kim et al. 2008, 2015; Di Pierro et al. 2009; Egert et al. 2009; Hsu et al. 2008; Nagao et al. 2009; Tominaga et al. 2009; Hu et al. 2012; Frati‐Munari et al. 1983; Liu et al. 2022; Coffey et al. 2004; Mirtaheri et al. 2015; Rouhani et al. 2019).

Herbal medicines containing *Ephedra sinica* , *Camellia sinensis* , *Garcinia cambogia*, *Gymnema sylvestre* , and *Glycyrrhiza glabra* were associated with reductions in at least one of the following biochemical parameters: total cholesterol (Dixit et al. 2018; González et al. 2004; Boozer et al. 2002; Di Pierro et al. 2009; Tominaga et al. 2009; Coffey et al. 2004; Mirtaheri et al. 2015), triglyceride (Dixit et al. 2018; Badmaev et al. 2001; Boozer et al. 2001, 2002; González et al. 2004; Hackman et al. 2006; Preuss et al. 2004; Di Pierro et al. 2009; Coffey et al. 2004), and LDL (Dixit et al. 2018; Badmaev et al. 2001; Preuss et al. 2004; Stendell‐Hollis et al. 2010; Boozer et al. 2002; Hsu et al. 2008; Tominaga et al. 2009; Mirtaheri et al. 2015), as well as increases in HDL (Dixit et al. 2018; Badmaev et al. 2001; Hackman et al. 2006; Preuss et al. 2004; Stendell‐Hollis et al. 2010; Boozer et al. 2002; Hsu et al. 2008).

Improvements in lipid parameters were heterogeneous across studies, with more pronounced reductions observed in participants with overweight or obesity. Reductions in total cholesterol ranged from 0.3 (Dixit et al. 2018; Cai et al. 2017) to 59.16 mg/dL (Cho et al. 2016), triglycerides from 2.4 (Dixit et al. 2018) to 135.8 mg/dL, and LDL from 2.9 (Hioki et al. 2004) to 48.6 mg/dL (Dixit et al. 2018), while increases in HDL ranged from 2.2 (Boozer et al. 2002) to 20.3 mg/dL (Tominaga et al. 2009).

The most effective intervention combined capsules containing *Moringa oleifera* , *Murraya koenigii* (L.) Spreng., and *Curcuma longa* with moderate‐intensity physical activity and a diet of 1800 kcal/day for 16 weeks. This protocol results in a mean decrease of 34.1 mg/dL (SD ± 31.68) in triglyceride levels, 28.0 mg/dL (SD ± 21.8) in total cholesterol, and 30.2 mg/dL (SD ± 18.4) in LDL, alongside an increase of 8.7 mg/dL (SD ± 8.9) in HDL (Dixit et al. 2018).

Other significant results were observed. One study reported a mean reduction of 56.0 mg/dL (SD ± 6.8) in triglyceride levels and 26.2 mg/dL in total cholesterol after 8 weeks of using *Sphaeranthus indicus* and *Garcinia mangostana* capsules (Stern, Peerson, Mishra, Sadasiva Rao, and Rajeswari 2013). Another study found a reduction of 24.2 mg/dL in total cholesterol following 12 weeks of intervention with berberine hydrochloride hydrate (*Rhizoma coptidis*) (Hu et al. 2012). A decrease of 39.3 mg/dL in triglycerides and 22.9 mg/dL in total cholesterol was observed after 6 weeks of using capsules containing *Camellia sinensis* , *Garcinia cambogia*, *Gymnema sylvestre* , and white kidney bean, combined with a dietary supplement (González et al. 2004).

One randomized, double‐blind, placebo‐controlled clinical trial reported a slight increase in triglyceride levels following the intervention with *Camellia sinensis* capsules for 3 months in obese women with polycystic ovary syndrome (Chan et al. 2006).

Table S6 provides details on the herbal medicines associated with improvements, worsening, or no changes in biochemical parameters.

### 3.6 Safety of Herbal Medicines in Weight Loss

The safety of herbal medicine use was not assessed in nine studies (González et al. 2004; Stendell‐Hollis et al. 2010; Sukohar et al. 2017; Di Pierro et al. 2009; Andersen and Fogh 2001; Ignjatovic et al. 2000; Dellalibera et al. 2006; Diepvens et al. 2006; Kovacs et al. 2001). The safety outcomes were primarily evaluated through participant‐reported adverse events (*n* = 64) and/or clinical parameter assessments (*n* = 41).

The most common adverse events included headache, gastrointestinal disorders (e.g., diarrhea, constipation, nausea, and vomiting), insomnia, anxiety, tachycardia, and skin rash. These adverse events were classified as mild by the authors of the included studies and were mainly associated with the use of *Ephedra sinica* (Armstrong et al. 2001; Boozer et al. 2001, 2002; Hackman et al. 2006; Greenway et al. 2004; Hioki et al. 2004; Astrup et al. 1992; Kim et al. 2008, 2014; Coffey et al. 2004; Cheon et al. 2020), *Camellia sinensis* (Chong et al. 2014; Greenway, Liu, et al. 2006; Greenway et al. 2004; Chan et al. 2006; Hsu et al. 2008; Lenon et al. 2012), *Citrus* sp. (Hancke et al. 2021; Lopez et al. 2013; Cai et al. 2017; Kang et al. 2018; Zhou et al. 2014), *Curcuma longa* (Dixit et al. 2018; Greenway, Liu, et al. 2006; Sengupta et al. 2012), *Garcinia cambogia* (Chong et al. 2014; Heymsfield et al. 1998), *Garcinia mangostana* (Stern, Peerson, Mishra, Sadasiva Rao, and Rajeswari 2013; Stern, Peerson, Mishra, Mathukumalli, and Konda 2013), *Moringa oleífera* (Dixit et al. 2018; Sengupta et al. 2012), *Sphaeranthus indicus* (Stern, Peerson, Mishra, Sadasiva Rao, and Rajeswari 2013; Stern, Peerson, Mishra, Mathukumalli, and Konda 2013), and *Coleus forskohlii* (Kamohara and Noparatanawong 2013). Serious adverse events, including increased blood pressure, irregular heart rate, exacerbation of asthma, and chest pain, were frequently associated with the use of *Ephedra sinica* (Armstrong et al. 2001; Boozer et al. 2001, 2002; Astrup et al. 1992; Coffey et al. 2004).

Forty‐one studies analyzed the potential toxicity of herbal medicines. Parameters related to thyroid function (e.g., thyroid‐stimulating hormone and free thyroxine), kidney function (e.g., creatinine and urea), liver function (e.g., alanine transaminase and aspartate aminotransferase) and heart function (e.g., heart rate and blood pressure), hematological parameters (e.g., hemoglobin and hematocrit), hormones (e.g., adrenocorticotrophic hormone), and electrolyte levels (e.g., sodium and potassium) were assessed. Most studies reported no significant changes in these parameters (Cao et al. 2023; Cho et al. 2017; Chong et al. 2014; Hancke et al. 2021; Leverrier et al. 2019; Badmaev et al. 2001; Greenway, Liu, et al. 2006; Stern, Peerson, Mishra, Sadasiva Rao, and Rajeswari 2013; Stern, Peerson, Mishra, Mathukumalli, and Konda 2013; Kang et al. 2018; Kim et al. 2008, 2015; Di Pierro et al. 2009; Egert et al. 2009; Henderson et al. 2005; Kamiya et al. 2011; Kamali et al. 2012; Nagao et al. 2007; Park et al. 2013; Cheon et al. 2020).

Table 5 provides a summary of adverse events associated with herbal products as reported in the included studies. The descriptions of these adverse events are consistent with those reported in the original primary studies.

**TABLE 5:** Safety parameters related to the interventions evaluated in the studies included in this scoping review (*n* = 45)^a^.

Intervention	Adverse event	Toxicity
Use of herbal medicines alone		
*Camellia sinensis* L. (Auvichayapat et al. 2008; Di Pierro et al. 2009; Hsu et al. 2008; Nagao et al. 2007)	Three participants developed mild constipation and 2 experienced abdominal discomfort following the green tea intervention. Similarly, 2 participants reported mild constipation and one experienced abdominal discomfort after the cellulose treatment. All symptoms were observed during the first week post‐treatment. No major adverse events were noted (Hsu et al. 2008).	Decrease in SBP [2.7 (SD ± 12.5), *p value* < 0.05], DBP [0.7 (SD ± 2.1), *p* value < 0.05], pulse rate [0.6 (SD ± 6.9), *p* value < 0.05]. There was no significant difference between the 2 groups in glutamic oxaloacetic transaminase [25.0 (SD ± 9.0) to 25.6 (SD ± 12.0)], glutamic pyruvic transaminase [34.3 (SD ± 23.9) to 33.6 (SD ± 24.2)], γ‐glutamyl transferase [51.9 (SD ± 80.4) to 54.1 (SD ± 82.6)], lactate dehydrogenase [308.0 (SD ± 93.7) to 303.9 (SD ± 94.5)], and alkaline phosphatase [202.6 (SD ± 82.3) to 199.1 (SD ± 81.9)] (Nagao et al. 2007). Decrease in blood pressure [SBP 127.0 (SD ± 14.8) to 124.3 (SD ± 14.2)] and DBP [76.9 (SD ± 10.4) to 75.8 (9.1)] and alkaline phosphatase [202.6 (SD ± 82.3) to 199.1 (SD ± 81.9)]. No changes in γ‐glutamyl transferase [51.9 (SD ± 80.4) to 54.1 (SD ± 82.6)]. Increase in urine vanillylmandelic acid [5.48 (SD ± 1.32) to 9.78 (SD ± 1.85)], *p* value < 0.05 (58). No changes in SBP [134.9 (SD ± 16.2) to 131.3 (SD ± 13.5)], DBP [82.9 (SD ± 9.3) to 81.7 (SD ± 9.1)], creatinine [0.8 (SD ± 0.2) to 0.8 (SD ± 0.1)], aspartate aminotransferase [25.5 (SD ± 9.4) to 23.2 (SD ± 8.2)], alanine aminotransferase [32.7 (SD ± 18.8) to 29.8 (SD ± 16.9)], and uric acid [5.7 (SD ± 1.4) to 5.8 (SD ± 1.5)] (Hsu et al. 2008).
*Camellia sinensis* L. (Chinese green tea) (Chan et al. 2006)	Amenorrhea or oligomenorrhea.	Not evaluated.
*Citrus unshiu* (Kang et al. 2018)	Gastrointestinal discomfort, mild diarrhea, headache, and dizziness.	Decrease in alanine aminotransferase [23.3 (SD ± 12.3) to 21.2 (SD ± 11.6), *p* value 0.016] and aspartate aminotransferase [24.1 (SD ± 8.1) to 22.4 (SD ± 8.1), *p* value 0.009]. Increase in gamma‐glutamyl transpeptidase [29.9 (SD ± 38.5) to 32.0 (SD ± 35.9), *p* value 0.153].
*Carum carvi* L. (Kazemipoor et al. 2013)	No adverse events were reported during the physical examinations.	No significant changes in urine‐specific gravity [1.021 (SD ± 0.006) to 1.022 (SD ± 0.006)], heart rate [78.06 (SD ± 9.11) to 77.51 (SD ± 8.11)], and blood pressure [DBP 75.48 (SD ± 7.89) to 75.9 (SD ± 6.80) and SBP 112.74 (SD ± 10.40) to 113.39 (SD ± 11.21)].
*Coleus forskohlii* (Kamohara and Noparatanawong 2013)	Abdominal discomfort increased bowel movements, soft stools, and/or diarrhea.	No significant changes in blood pressure and heart rate between values at baseline and at 8 weeks (*p* value > 0.005)
*Coptis chinensis* (Hu et al. 2012)	Not evaluated.	Decrease in aspartate aminotransferase (18%, *p* value 0.025). No changes in inflammatory factors [high‐sensitivity C‐reactive protein level (*p* value 0.711), erythrocyte sedimentation rate (*p* value 0.711), blood hormones (calcitriol, cortisol (*p* value 0.325), adrenocorticotrophic hormone (*p* value 0.190), thyroid‐stimulating hormone (*p* value 0.117), free T4 (*p* value 0.929)), hormone binding globulin (*p* value 0.240), albumin (1.5%, *p* value 0.31), alkaline phosphatase (−5.4%, *p* value 0.125), and total bilirubin levels (9.5%, *p* value 0.642)].
*Ephedra sinica* (Kim et al. 2014)	Headache, nausea and dyspepsia were classified as minor adverse events.	No significant changes in heart rate (*p* value 0.375) and blood pressure (SBP: *p* value 0.646; and DBP: *p* value 0.562).
*Garcinia cambogia* (Heymsfield et al. 1998)	There were no significant differences between the placebo and intervention groups	Not evaluated.
*Glycyrrhiza glabra* L. (Bell et al. 2011)	NR.	Study 1: increase aspartate aminotransferase [23.7 (SD ± 2.5) to 26.7 (SD ± 2.3), *p* value 0.020]. No significant changes in bilirubin [0.5 (SD ± 0.1) to 0.4 (SD ± 0.1)], creatinine [1.0 (SD ± 0.1)], adiponectin [17.8 (SD ± 1.7) to 19.0 (SD ± 2.7)], protein [6.9 (SD ± 0.1)], albumin [4.3 (SD ± 0.1)], alkaline phosphatase [67.5 (SD ± 6.8) to 65.3 (SD ± 6.5)], alanine transaminase [23.5 (SD ± 4.0) to 28.9 (SD ± 5.2)], insulin resistance [1.6 (SD ± 0.3) to 2.0 (SD ± 0.4)], and blood glucose [84.4 (SD ± 1.9) to 85.7 (SD ± 2.9)]. Study 2: increase alkaline phosphatase [70.4 (SD ± 5.0) to 71.2 (SD ± 4.2) *p* value 0.003] and alanine transaminase [20.3 (SD ± 1.9) to 21.0 (SD ± 2.2), *p* value 0.04]. Not significant change in bilirubin [0.8 (SD ± 0.2) to 0.6 (SD ± 0.1)], creatinine [1.2 (SD ± 0.1) to 1.1 (SD ± 0.0)], protein [7.1 (SD ± 0.1)], albumin [4.5 (SD ± 0.1) to 4.6 (SD ± 0.1)], aspartate aminotransferase [25.8 (SD ± 3.4) to 27.8 (SD ± 3.1)], insulin resistance [0.7 (SD ± 0.0) to 0.8 (SD ± 0.0)], and blood glucose [88.5 (SD ± 2.7) to 93.8 (SD ± 1.3)].
*Hoodia gordonii* (Blom et al. 2011)	Disturbance of skin sensation, headache, dizziness, nausea, flushing, vomiting, malaise, fatigue, flatulence, eructation, gas pain, and edema were reported and not classified as serious.	Increase in blood pressure (SBP 5.9 to 15.9 mmHg, *p* value < 0.05; DBP 4.6 to 11.5 mmHg, *p* value < 0.05), pulse (5.2 to 9.9 beats/min), bilirubin (0.20 to 0.58, *p* value < 0.05), alkaline phosphatase (4.7 to 8.8, *p* value < 0.05). Decrease in blood urea nitrogen (1.3 to 2.87, *p* value < 0.05). There were no significant differences noted in creatinine, γ‐glutamyl transferase, aspartate aminotransferase, and alanine aminotransferase between the intervention and control group.
Quercetin (Egert et al. 2009)	NR.	Decrease in blood pressure (SBP −7.7, *p* value < 0.001; DBP −4.1, *p* value 0.001). Decrease in SBP (2.6, *p* value < 0.01 in all participants; 2.9, *p* value < 0.01 in the subgroup of participants diagnosed with hypertension; and 3.7 mmHg, *p* value < 0.001 in the subgroup of younger participants aged 25 to 50 years).
Raspberry (Khazaal et al. 2015)	NR.	Decrease in oxidative stress markers glutathione (0.55 SD ± 0.04), malondialdehyde (1.65 SD ± 0.34), and 8‐isoprostane (170.33 SD ± 7.62).
Use of herbal medicines in association		
Acetyl L‐carnitine, guarana extract, *Ma Huang*, L‐tyrosine, fisetin, magnesium phosphate, white willow bark extract, bitter orange, ginger root, and vitamin A (Armstrong et al. 2001)	Exacerbation of anxiety, increased heart rate (in the first days of use) and a sensation of “warmed blood”.	Not evaluated.
*Bofutsushosan Streptococcus thermophiles*, *Lactobacillus plantarum* , *Lactobacillus acidophilus* , *Lactobacillus rhamnosus* , *Bifidobacterium lactis* , *Bifidobacterium longum* , and *Bifidobacterium breve* (Lee et al. 2014)	Not evaluated.	No significant changes in blood urea nitrogen (*p* value 0.731), creatinine (*p* value 0.063), aspartate aminotransferase (*p* value 0.429), and alanine aminotransferase (*p* value 0.720)
*Camellia sinensis* , *Cassia obtusifolia* , and *Sophora japonica* (Lenon et al. 2012)	Mild adverse events, such as headache and nausea, occurred during the first treatment period.	No significant changes in SBP [122.8 (SD *±* 14.1) to 117.8 (SD *±* 14.0), *p* value 0.783], DBP [80.8 (SD *±* 9.9) to 81.1 (SD *±* 9.8), *p* value 0.853], heart rate [72.6 (SD *±* 8.5) to 74.6 (SD *±* 9.2), *p* value 0.084], and renal and liver tests before and after the intervention period.
*Citrus aurantium* , rhubarb, coptis, and *Semen cassiae* decoction (Zhou et al. 2014)	No serious adverse events were reported. There were seven minor adverse events reported, with 4 in the treatment group and 3 in the control group (*p* value 0.578). In one case, a transient skin rash was reported in the treatment group, which disappeared without any treatment.	NR.
*Citrus bergamia* Risso and *Eurycoma longifolia* (Hancke et al. 2021)	Mild allergic rhinitis, mild throat irritation, moderate vomiting, diarrhea, stomach pain, moderate abdominal pain with loose stools, moderate fever, weakness, headache, moderate ankle sprain, and mild constipation.	No significant changes in cortisol levels [low dose group 16.26 (SD ± 10.40) to 10.89 (SD ± 5.29) and high dose group 15.67 (SD ± 8.11) to 10.42 (SD ± 4.94), blood pressure, and heart rate].
*Citrus bergamia* Risso extract, plant sterol esters, orange oil, vitamin C, vitamin B6, vitamin B12, and folic acid (Cai et al. 2017)	Dizziness.	NR.
*Coicis semen*, *Semen castaneae*, *Raphanus sativus* L., *Schisandra chinensis* Baill, Liriopis tuber, *Ephedrae herba*, *Platycodon grandiflorum* , and *Acori tatarinowii Rhizoma* (Park et al. 2013)	NR.	No significant changes in blood pressure [SBP 122.7 (SD ± 14.6) to 122.6 (SD ± 1.6), *p* value 0.361 and DBP 76.6 (SD ± 8.10) to 77.5 (SD ± 7.60), *p* value 0.746], aspartate aminotransferase [27.9 (SD ± 16.2) to 21.9 (SD ± 12.3), *p* value 0.054], alanine aminotransferase [28.9 (SD ± 18.9) to 14.5 (SD ± 10.4), *p* value 0.357], blood urea nitrogen [14.5 (SD ± 10.4) to 12.7 (SD ± 4.10), *p* value 0.700], creatinine [0.78 (SD ± 0.18) to 0.79 (SD ± 0.190), *p* value 0.3731], C‐reactive protein [0.78 (SD ± 0.18) to 0.79 (SD ± 0.19), *p* value 0.373].
*Ephedra sinica Stapf*, *Angelica gigantis Radix*, *Atractylodis rhizoma Alba*, *Coicis Semen*, *Cinnamomi cortex*, *Paeonia lactiflora* , and *Glycyrrhiza uralensis* (Cheon et al. 2020)	Participants reported moderate headaches, as well as mild diarrhea, herpes zoster, cholelithiasis, allergic dermatitis, concussion, peripheral swelling, hypertonic bladder, uterine leiomyoma, and uterine polyp.	No significant changes in blood pressure [SBP 126.9 mmHg (124.4–129.4), to 124.9 mmHg (122.4–127.3), *p* value 0.705, and DBP 79.7 mmHg (78.1–81.4) to 79.6 mmHg (77.9–81.4), *p* value 0.381], creatinine [0.68 (0.65–0.71) to 0.70 (0.67–0.72)], blood urea nitrogen [12.4 (11.6–13.1) to 12.5 (11.7–13.2)] alanine transaminase [28.8 (23.6–34.0) to 25.7 (21.7–29.7)], and aspartate aminotransferase [25.4 (22.5–28.3) to 22.8 (20.7–24.9)].
*Ephedra sinica* and *Evodia rutaecarpa* (Kim et al. 2008)	In the *Ephedra* group, adverse events included palpitations, insomnia, dry mouth, and gastrointestinal symptoms such as anorexia, nausea, vomiting, and constipation. The *Evodia* group also experienced increases in symptoms including palpitations, trembling, dizziness, nervousness, and gastrointestinal issues. The placebo group reported increased insomnia and skin eruptions.	*Ephedra sinica* group: decrease in aspartate transaminase [17.7 (SD ± 4.1) to 15.5 (SD ± 3.0)] and blood urea nitrogen [13.2 (SD ± 3.4) to 11.8 (SD ± 2.8)]. *Evodia rutaecarpa* group: decrease in blood urea nitrogen [11.7 (SD ± 3.5) to 10.6 (SD ± 2.8)].
Ephedrine and caffeine (Astrup et al. 1992)	Adverse events such as insomnia, palpitations, and tremors were transient and disappeared after 6 to 14 days of treatment.	NR.
*Foeniculum vulgare* , *Urtica dioica* , *Daucus carota* , *Trifolium pratense* , and *Curcuma longa* (Rouhani et al. 2019)	NR.	Decrease in alanine aminotransferase (*p* value 0.010) and aspartate aminotransferase (*p* value 0.006).
*Ganoderma lucidum*, *Coptis chinensis*, *Astragalus membranaceu*, *Nelumbo nucifera* Gaertn., and *Fructus aurantii* (Cao et al. 2023)	Participants experienced slight gastrointestinal symptoms, including decreased hyper appetite, mild nausea, and increased frequency of stools. These side effects were quickly alleviated by taking the intervention after meals or reducing the dose.	Decrease in blood pressure [blood pressure −7.99 mmHg (−13.29, −2.598), *p* < 0.05 and DBP −8.67 mmHg [−12.59, −4.64], *p* < 0.001] and glutamyl transferase (42.22 ± 23.57 to 38.65 ± 15.27). No significant change in alanine aminotransferase (30.44 ± 10.92 to 30.17 ± 8.77), aspartate aminotransferase (21.67 ± 4.23 to 21.06 ± 3.69), total bilirubin (15.31 ± 4.72 to 14.36 ± 3.15), total protein (73.49 ± 4.54 to 73.44 ± 3.94), and albumin (41.68 ± 2.4 to 42.64 ± 2.39). The electrolytes, routine urine, blood coagulation, and routine blood of all subjects were within the normal range, and there was no significant change.
*Garcinia cambogia*, *Camellia sinensis* , *Coffea arabica* , and *Lagerstroemia speciosa* (Chong et al. 2014)	Moderate cystitis, moderate toothache, mild diarrhea, mild upper respiratory tract infection, moderate bilateral heel spurs, and mild bronchitis.	No changes in alanine transaminase [0.44 (SD ± 0.23) to 0.47 (SD ± 0.21)], aspartate aminotransferase [0.40 (SD ± 0.12) to 0.42 (SD ± 0.10)], alkaline phosphatase [1.23 (SD ± 0.33) to 1.26 (SD ± 0.29)], gamma‐glutamyl transferase [0.48 (SD ± 0.30) to 0.46 (SD ± 0.24)], bilirubin [10.8 (SD ± 5.0) to 10.7 (SD ± 4.7)], creatinine [70.3 (SD ± 13.4) to 72.4 (SD ± 13.5)], and urea [4.81 (SD ± 1.16) to 4.58 (SD ± 1.30)].
*Garcinia cambogia*, *Gymnema sylvestre* , white kidney bean extract, green tea, vitamins (C, B1, B2, B3, B6, B9, B12), panthotenic acid, choline, inositol, 4‐aminobenzoic acid, calcium, phosphorus, chromium, and vanadium (intervention 1). Psyllium, chitosan, glucomannan, and pectin (intervention 2). Ginger extract, green tea extract, complex B vitamins, conjugated linoleic acid, taurine, lecithin, L‐carnitine, inositol phosphate, vitamin C, and capsaicin (intervention 3) (González et al. 2004)	Not evaluated.	No changes in nervous or cardiovascular systems (data not reported).
Pill 1: *Garcinia camboja* extract, guarana extract and ephedra extract, oolong tea extract, garlic extract, tulsi extract, *Eleutherococcus senticosis* extract, green tea extract, *Ginkgo biloba* extract, *Gymnema sylvestre* extract, silymarin extract, and red wine polyphenols. Pill 2: vitamins (A, C, D, E, K, B1, B2, B3, B6, B9, and B12), biotin, pantothenic acid, calcium, iron, phosphorus, iodine, magnesium, zinc, selenium, copper, manganese, chromium, molybdenum, chloride, potassium, boron, nickel, silicon, tin, vanadium, lutein, choline, coenzyme Q‐10, L‐glutathione, L‐methionine, bioflavonoids, L‐carnitine, taurine, and beta‐sitosterol. Pill 3: Eicosapentaenoic acid and docosahexaenoic acid. All the participants received the 3 intervention pills (Hackman et al. 2006)	Reported adverse events included dry mouth, nervousness, palpitations, decreased appetite, dizziness, fatigue, headache, insomnia, and nausea.	Decrease in heart rate (*p* value < 0.0003). No changes in blood pressure (SBP *p* value 0.609 and DBP *p* value 0.518), electrocardiogram, urinalysis, and blood histology.
Green tea leaf extract, guarana extract, bitter orange, white willow bark extract ginger root, proprietary charge thermoblend (L‐tyrosine, L‐carnitine, and naringin) and pantothenic acid (intervention 1) Phenylephrine (intervention 2) (Greenway, de Jonge‐Levitan, et al. 2006)	High blood pressure, unspecified musculoskeletal and neurological events, headache/migraine, and anxiety were reported in intervention 1. Rash, menstrual cramps, unspecified neurological events, headache/migraine, insomnia, oral complains, upper respiratory were reported in intervention 2. These were classified as neither serious nor significant.	Decrease in SBP [8.8 (SD ± 3.1)], DBP [8.7 (SD ± 2)], and pulse rate [0.9 bpm (SD ± 3.2)].
*Imperata cylindrica* Beauvoi, *Citrus unshiu* Markovich, and *Evodia officinalis* (Cho et al. 2017)	Dyspepsia, nausea, epigastric soreness, diarrhea, constipation, upper respiratory tract infections, headache, dizziness, rash, musculoskeletal pain, and fatigue were reported as minor events and were classified as non‐serious adverse events.	No significant changes in blood pressure [(SBP 122.7 (SD ± 12.4) to 119.2 (SD ± 12.1), *p* value 0.454) and DBP (77.4 (SD ± 9.5) to 73.1 (SD ± 10.4), *p* value 0.583)], blood urea nitrogen [11.8 (SD ± 2.7) to 11.3 (SD ± 2.5), *p* value 0.127], creatinine [0.7 (SD ± 0.2) to 0.8 (SD ± 0.2), *p* value 0.430], alanine transaminase [19.4 (SD ± 6.9) to 19.8 (SD ± 10.2), *p* value 0.360], and aspartate aminotransferase [21.4 (SD ± 5.5) to 20.3 (SD ± 4.8), *p* value 0.154].
*Ma Huang* and guarana (Boozer et al. 2001)	Irritability, dizziness, insomnia, anxiety, headache, blurred vision, palpitations, constipation, diarrhea, stomach pain, heartburn, nausea, dry mouth, and chest pain.	No changes in standard electrolytes (data not reported).
*Ma Huang* and kola nut (Boozer et al. 2002)	Chest pain, loud heartbeat, palpitations, elevated blood pressure, irregular heartbeat, multifocal ventricular events, anxiety, disorientation, dizziness, insomnia, irritability, gastroesophageal reflux disorder, dry mouth, nausea, and gallbladder removal were identified and classified as not significant.	No significant changes in alanine aminotransferase, aspartate aminotransferase, electrolytes, and creatinine (data not reported). Slight decrease in blood pressure (*p* value < 0.05) and increase in heart rate. Increase in heart rate [1 (SD ± 14 bpm), *p* value 0.026].
*Ma Huang*, kola nut and white willow bark (Coffey et al. 2004)	Exacerbated depression, atrial fibrillation, exacerbation of asthma, low back pain and compression fracture of L1 were identified and classified as serious adverse events.	No temporal relationship was identified between the intervention and SBP (*p* value 0.760) and DBP (*p* value 0.490). There was a significant difference in pulse rates observed in the 2 groups at the end of the study (*p* value 0.09).
*Moringa oleifera* , *Murraya koenigii* L. and *Curcuma longa* L. (Dixit et al. 2018; Sengupta et al. 2012)	No serious adverse events were observed in this study. Minor events reported by study participants included fever, gastritis, headache, itching, loose stools, acidity, excessive appetite, abdominal and back pain, and dehydration.	NR.
*Ma Huang*, kola nut seed, chromium, green tea, adrenal gland, L‐phenylalanine, ginger root, Fo‐ti root, Lycii berry fruit, *Siberian ginseng* root, cinnamon bark, zinc, vanadium (aspartate), *Astragalus*, caffeine, magnesium, *Ginkgo biloba* , and pyridoxal α‐ketoglutarate (phase 1) Caffeine and ephedrine (phase 2) (Greenway et al. 2004)	Respiratory, gastrointestinal, and oral events (not specified), unspecified pain, headache, hair loss, and neuropsychiatric and skin problems (not specified) were reported in phase 2. These events were classified as neither serious nor significant.	No changes in physical examination, electrocardiogram, laboratory tests, or urinalysis (glucose, ketones, specific gravity, blood, and protein) (data not reported). Decrease in pulse rate (0.3 bpm, *p* value < 0.03). Not significant change in blood pressure (decrease 4/1 mmHg, *p* value > 0.1).
*Phyllanthus emblica* L., *Terminalia chebula* Retz., and *Terminalia belerica* Retz (Kamali et al. 2012)	NR.	Decrease in alanine aminotransferase [24.10 (SD ± 9.18) to 18.90 (SD ± 8.07), *p* value 0.003], uric acid [6.09 (SD ± 1.07) to 5.78 (SD ± 1.17), *p* value 0.05]. No changes in blood pressure (initial measurement/after a 5‐min interval) [SBP 119.66 (SD ± 17.80)/117.50 (SD ± 18.03) to 120.22 (SD ± 20.08)/119.77 (SD ± 18.61)]; [DBP 78.83 (SD ± 10.05/78.83) (SD ± 10.56) to 80.00 (SD ± 12.14)/79.77 (SD ± 10.74)].
Prograde Metabolism (thiamin, riboflavin, niacin, vitamin B6 and B12, biotin, pantothenic acid, chromium and a blend with raspberry ketone, caffeine anhydrous, bitter orange, ginger root extract, garlic root extract, cayenne extract, L‐theanine, and *Piper nigrum* ) (Lopez et al. 2013)	NR.	No change in blood pressure [SBP 119.8 mmHg (SD ± 10.0) to 118.1 mmHg (SD ± 10.3), DBP 77.8 mmHg (SD ± 8.7) to 76.9 mmHg (SD ± 9.1)], and heart rate [70.1 (SD ± 8.2) to 70.1 (SD ± 8.4) bpm], adiponectin [10.20 (SD ± 0.81) to 9.93 (SD ± 0.76), *p* value < 0.150], interleucin‐6 [0.45 (SD ± 0.83) to 0.34 (SD ± 0.94), *p* value < 0.890] and tumor necrosis factor—*α* [1.71 (SD ± 1.16) to 1.58 (SD ± 1.08), *p* value < 0.490].
Rhubarb root and stem (*Radix et Rhizoma Rhei*), *Astragulus* root (*Radix astragali*), red sage root (*Radix salviae miltiorrhizae*), turmeric (rhizoma *Curcumae longae*) and ginger (rhizoma *Zingiberis officinalis*) (Greenway, Liu, et al. 2006)	Dermatological issues, abdominal pain, diarrhea, nausea, vomiting, vaginal irritation, musculoskeletal pain, headache, migraine, gum irritation, toothache, other oral symptoms, flu, and upper respiratory infection symptoms.	No changes in blood pressure and heart rate, physical examinations, and electrocardiogram (data not reported).
*Scutellariae radix* and *Platycodi radix* (Cho et al. 2013)	Anorexia, nausea, indigestion, hunger, headache, abdominal pain, loose stools, diarrhea, constipation, facial edema, insomnia, itching, fatigue, palpitations, feelings of satiety, improvement in constipation, changes in appetite (either increase or suppression), increased gastric acid secretion, and thirst were reported. These events were classified as not significant.	No significant changes in blood pressure [SBP −11.30 mmHg (SD ± 13.92) and DBP −8.20 mmHg (SD ± 10.60), *p* value 0.368], high‐sensitivity C‐reactive protein [1.59 (SD ± 6.25), *p* value 0.251], bilirubin [0.04 (SD ± 0.14), *p* value 0.112], gamma‐glutamyl transpeptidase [−1.26 (SD ± 7.65), *p* value 0.487], blood urea nitrogen [−1.43 (SD ± 2.80), *p* value 0.474], creatinine [0.03 (SD ± 0.10), *p* value 0.223], and uric acid [−0.09 (SD ± 0.59), *p* value 0.621].
*Scutellariae radix*, *Glycyrrhizae radix*, *Platycodi radix*, *Gypsum fibrosum*, *Atractylodis rhizoma*, *Rhei rhizoma*, *Schizonepetae spica*, *Gardeniae fructus*, *Paeoniae radix*, *Cnidium rhizoma*, *Angelicae radix*, *Menthae herba*, *Ledebouriellae radix*, *Ephedrae herba*, *Forsythiae fructus*, *Zingiberis fhizoma*, *Talcum*, and *Natrium sulphuricum* (Hioki et al. 2004)	Not evaluated.	Decrease in uric acid [6.5 (SD ± 1.2) to 5.1 (SD ± 0.4)].
*Sphaeranthus indicus* and *Garcinia mangostana* (Stern, Peerson, Mishra, Sadasiva Rao, and Rajeswari 2013; Stern, Peerson, Mishra, Mathukumalli, and Konda 2013)	No major adverse events were reported during this study. Minor adverse events, including headache, nausea, gastrointestinal irritation, and pain in the back, leg, ankle, and joints, were evenly distributed between the 2 groups.	No significant changes in total protein [7.1 (SD ± 0.1) to 7.3 (SD ± 0.1), *p* value 0.8], alkaline phosphatase [138.5 (SD ± 3.8) to 125.1 (SD ± 2.1), *p* value 0.5], serum glutamic‐oxaloacetic transaminase [26.7 (SD ± 0.7) to 23.5 (SD ± 0.6), *p* value 0.8], serum glutamic pyruvic transaminase [33.3 (SD ± 1.0) to 30.7 (SD ± 0.9), *p* value 0.5], bilirubin [0.7 (SD ± 0.03) to 0.7 (SD ± 0.0), *p* value 0.06], SBP [124.5 (SD ± 2.0) to 121.8 (SD ± 1.6), *p* value 0.2], DBP [83.7 (SD ± 1.4) to 80.1 (SD ± 1.1), *p* value 0.08], pulse rate [73.8 (SD ± 1.1) to 72.8 (SD ± 1.0), bpm, *p* value 0.1], creatinine [0.8 (SD ± 0.01) to 0.9 (SD ± 0.01), *p* value 0.5], SBP [−1.03 mmHg (SD ± 2.12), *p* value 0.495], DBP [−0.76 mmHg (SD ± 1.73), *p* value 0.437], pulse rate [0.83 (SD ± 1.63), *p* value 0.155], serum glutamic‐oxaloacetic transaminase [−3.83 (SD ± 1.12), *p* value 0.690], and serum glutamic pyruvic transaminase [−3.38 (SD ± 1.57), *p* value 0.558] (Stern, Peerson, Mishra, Sadasiva Rao, and Rajeswari 2013).

Abbreviations: Bpm: beats per minute; DBP: diastolic blood pressure (mmHg); NR: not reported; SBP: systolic blood pressure (mmHg); SD: standard deviation.

^a^ Although 65 studies reported the evaluation of the safety of herbal medicine products, only 45 studies provided safety data.

## 4 Discussion

This scoping review provides evidence on the therapeutic effects and safety of herbal medicine products for weight loss. Unlike previous reviews, this study does not impose restrictions on participant health conditions (Payab et al. 2020; Hasani‐Ranjbar et al. 2009; Maunder et al. 2020; Park et al. 2022), the herbal medicines evaluated (Cercato et al. 2015; Payab et al. 2020; Maunder et al. 2020; Park et al. 2022), or methodological aspects (e.g., study design and setting) (Pittler and Ernst 2004; Cercato et al. 2015), thereby providing a broader perspective.

### 4.1 Efficacy and Safety of Herbal Medicines for Weight Loss

The observed efficacy of herbal medicines was mainly seen in participants diagnosed with overweight and/or obesity, conditions often associated with metabolic disorders and imbalances in intestinal microbiota. These alterations may serve as potential therapeutic targets for herbal medicines, which have been shown to influence metabolic processes as well as microbiota balance (An et al. 2019; Zhang et al. 2023). This dual mechanism also helps explain the observed improvements in lipid markers in participants following the intervention. Supporting this, a previous meta‐analysis demonstrated that weight loss, regardless of the intervention, results in significant reductions in total cholesterol and triglycerides, increases in HDL, and modest changes in LDL levels (Hasan et al. 2020).

*Camellia sinensis* , popularly known as green tea, was the most frequently evaluated herbal medicine in the reviewed studies (Bagchi et al. 2022; Chong et al. 2014; Sindler and Sindler 2001; González et al. 2004; Hackman et al. 2006; Greenway et al. 2004; Stendell‐Hollis et al. 2010; Auvichayapat et al. 2008; Di Pierro et al. 2009; Chan et al. 2006; Diepvens et al. 2006; Hsu et al. 2008; Nagao et al. 2009, 2007; Greenway, de Jonge‐Levitan, et al. 2006; Lenon et al. 2012). Its potential benefits, including weight loss, improved insulin resistance, enhanced glucose metabolism, and better lipid distribution, are attributed to its bioactive components such as catechins (e.g., epigallocatechin gallate), caffeine, and polyphenols (Zhao et al. 2022). Eight out of 16 studies identified improvements in all evaluated parameters after the use of green tea (Bagchi et al. 2022; Chong et al. 2014; Sindler and Sindler 2001; González et al. 2004; Hackman et al. 2006; Greenway et al. 2004; Auvichayapat et al. 2008; Nagao et al. 2007). Two studies did not observe any improvements (Stendell‐Hollis et al. 2010; Chan et al. 2006) and one study found that combining green tea with other herbal medicines, such as *Paullinia cupana* , *Citrus aurantium* , *Salix alba* , and *Zingiber officinale* , led to weight gain and adverse events, including increased blood pressure, insomnia, and anxiety (Greenway, de Jonge‐Levitan, et al. 2006).

Studies that reported reductions in all anthropometric parameters often combined green tea with other herbal medicines (e.g., *Garcinia cambogia* and *Ephedra sinica* ) (Sindler and Sindler 2001; González et al. 2004; Greenway et al. 2004), dietary supplements (e.g., L‐tyrosine and L‐carnitine) (Bagchi et al. 2022; Sindler and Sindler 2001; González et al. 2004; Hackman et al. 2006; Greenway et al. 2004), as well as diet or moderate‐intensity physical activity (Chong et al. 2014; Greenway et al. 2004; Auvichayapat et al. 2008; Di Pierro et al. 2009). The synergistic effects of phytonutrients, herbal medicines, and lifestyle changes are known to enhance weight loss (Jurgens et al. 2010), possible explaining why green tea alone was not effective in improving all evaluated parameters in some studies. Given the positive outcomes observed in most studies, together with uncertainties regarding its efficacy as a stand‐alone therapy and the occurence of adverse events (e.g., abdominal discomfort, amenorrhea, and anxiety) (Chong et al. 2014; Greenway et al. 2004; Chan et al. 2006; Hsu et al. 2008; Greenway, de Jonge‐Levitan, et al. 2006; Lenon et al. 2012), the risk–benefit profile of green tea for weight loss requires careful evaluation.

The second most investigated herbal medicine was *Ephedra sinica* , commonly known as *Ma Huang*. Among the 13 studies reviewed, 10 reported positive outcomes across all parameters evaluated regarding weight loss (Armstrong et al. 2001; Boozer et al. 2001, 2002; Hackman et al. 2006; Greenway et al. 2004; Hioki et al. 2004; Kim et al. 2008, 2014; Coffey et al. 2004; Cheon et al. 2020). The weight loss efficacy of *Ephedra sinica* is primarily attributed to ephedrine alkaloids, such as ephedrine, which exhibit thermogenic properties (Schaneberg et al. 2003). These compounds increase energy expenditure, stimulate the central nervous system, enhance metabolic rate, and reduce appetite (Schaneberg et al. 2003).

Despite its potential benefits, *Ephedra sinica* raises significant safety concerns. Its use has been associated with toxicity, including increased levels of aspartate aminotransferase and alanine aminotransferase (Park et al. 2013); cardiovascular events, such as elevated heart rate, irregular heartbeats, atrial fibrillation, and increased blood pressure (Armstrong et al. 2001; Boozer et al. 2001, 2002; Coffey et al. 2004); and psychiatric symptoms, such as heightened anxiety, irritability, and insomnia (Armstrong et al. 2001; Boozer et al. 2001, 2002; Hackman et al. 2006; Astrup et al. 1992). Other reported adverse events include blurred vision, chest pain, tremors, headache, gastrointestinal discomfort, and asthma exacerbation (Armstrong et al. 2001; Boozer et al. 2001, 2002; Greenway et al. 2004; Astrup et al. 1992; Kim et al. 2008, 2014; Coffey et al. 2004).

Due to these serious adverse events, *Ephedra*‐based products were banned in the United States (2004) and Europe (2015) (National Institutes of Health 2004; EFSA Panel on Food Additives and Nutrient Sources added to Food (ANS) 2013). In Brazil, *Ephedra sinica* is not freely marketed and is included in the regulatory agency's list of substances under special control (Ordinance 344/1998) (Brasil. Agência Nacional de Vigilância Sanitária 2022b). However, *Ephedra*‐based herbal medicines remain readily accessible online. Notably, apart from four studies conducted in South Korea between 2008 and 2020 (Kim et al. 2008, 2014; Park et al. 2013; Cheon et al. 2020), where the use and marketing of *Ephedra* is authorized, the remaining studies were published between 2004 and 2005.

*Garcinia cambogia*, also referred to as Malabar tamarind or brindle berry, was evaluated in 9 studies (Chong et al. 2014; Badmaev et al. 2001; González et al. 2004; Hackman et al. 2006; Preuss et al. 2004; Tripathy et al. 2013; Heymsfield et al. 1998; Kovacs et al. 2001), and 7 of which reported improvements in all anthropometric parameters (Chong et al. 2014; Badmaev et al. 2001; González et al. 2004; Preuss et al. 2004; Tripathy et al. 2013; Gupte et al. 2020; Heymsfield et al. 1998). In one study, improvements were observed only in hip circumference, neck circumference, and waist circumference (Gupte et al. 2020). The main active compound in *Garcinia cambogia*, hydroxycitric acid, is believed to be associated with weight loss through its ability to inhibit ATP‐citrate lyase, promote hepatic glycogen accumulation, increase serotonin release, and modulate lipid transport and oxidation (Astell et al. 2013). These mechanisms collectively contribute to appetite suppression and weight loss promotion (Astell et al. 2013).

Despite its potential benefits, it was reported that *Garcinia cambogia* was associated with adverse events, including headache, upper respiratory tract symptoms, abdominal discomfort, constipation, diarrhea, and anorexia (Chong et al. 2014; Heymsfield et al. 1998). Besides, it was linked to increased transaminase enzyme levels, which may indicate liver damage (García‐Cortés et al. 2016).

*Citrus* sp. was associated with improvements in at least one anthropometric parameter in participants diagnosed with obesity or being overweight in all 5 studies reviewed (Cho et al. 2017; Hancke et al. 2021; Cai et al. 2017; Kang et al. 2018; Zhou et al. 2014). Following the regulatory ban on *Ephedra*, many manufacturers reformulated their products to be “ephedra‐free,” often replacing it with *Citrus* sp. (e.g., *Citrus aurantium* ) (Bent et al. 2004). Citrus flavonoids, especially naringenin and nobiletin, have demonstrated potential significant lipid‐lowering effects by reducing lipid accumulation in the liver, preventing excessive lipoprotein production, and improving insulin sensitivity (Assini et al. 2013).

These flavonoids also exhibit anti‐inflammatory properties and slow the progression of atherosclerosis, with their beneficial effects mediated by the normalization of hepatic fatty acid metabolism and improved insulin signaling. This highlights their potential in weight management (Assini et al. 2013). While *Citrus* sp. was generally considered safe in the included studies, reported adverse events included skin rash, gastrointestinal complaints, headache, dizziness, musculoskeletal pain, and weakness (Cho et al. 2017; Hancke et al. 2021; Kang et al. 2018; Zhou et al. 2014). No toxicity or liver function abnormalities were reported.

Last, *Glycyrrhiza* sp., popularly known as licorice or sweet wood, demonstrated improvements in anthropometric parameters in 4 of the 5 studies identified (Bell et al. 2011; Tominaga et al. 2009; Mirtaheri et al. 2015; Cheon et al. 2020). After the intervention, one study reported an increase in body fat mass, hip circumference, and waist circumference (Bell et al. 2011). The observed weight loss in the other studies is likely due to licochalcone A, which enhances thermogenesis, lipolysis, and fat oxidation while reducing lipogenesis (Lee et al. 2018). Adverse events associated with *Glycyrrhiza* sp. were evaluated in the 5 studies, with 2 reporting mild events such as headache, diarrhea, and dermatitis.

### 4.2 Long‐Term Safety of Herbal Medicines for Weight Loss

Most studies reported adverse events in vague terms (e.g., gastrointestinal complaints) without providing details on their severity, management strategies, or associated risk factors. Only 5 assessed safety outcomes during a follow‐up period after the intervention (ranging from 2 to 56 days) (Blom et al. 2011; Nagao et al. 2009; Tominaga et al. 2009; Gupte et al. 2020; Salunke et al. 2019). However, none of these studies clearly reported the outcomes observed during this period, highlighting a significant gap in the evaluation of the long‐term safety of herbal medicines.

This lack of detailed reporting may compromise user safety by fostering a potentially misleading perception that herbal medicines are risk‐free or that most adverse events are mild. Furthermore, the limited follow‐up periods prevent drawing conclusions about the long‐term sustainability of weight loss and its associated risks.

Therefore, while several studies have demonstrated the efficacy of herbal medicine products in promoting weight loss and improving anthropometric and biochemical outcomes, significant concerns remain regarding long‐term safety.

### 4.3 Limitations and Strengths of the Review

This review has both limitations and strengths. One primary limitation is the inclusion of 22 studies identified through manual search. These studies did not employ commonly recognized terms in scientific literature, such as “natural product”, “herbal medicine”, and “medicinal plant”, in their titles, abstracts, or keywords. Instead, most studies referenced only the scientific or popular name of herbal medicines.

Among the strengths of this review is its comprehensive synthesis of evidence on herbal medicines used for weight loss, including their effects on anthropometric and biochemical parameters, as well as associated risks. These findings contribute to the rational and safe use of herbal medicine products, ensuring therapeutic efficacy, user safety, and the promotion of responsible self‐medication (Galato et al. 2009). Moreover, the results support health decision‐making processes, including prescribing practices and the potential integration of these products into public health protocols and guidelines, contingent upon future studies confirming their safety.

The findings may not be fully generalizable to broader populations, particularly those outside the regions where most studies were conducted (Asia, North America, and Europe). Representation from Latin America, Oceania, and Africa was limited, and the demographic characteristics of participants varied across studies. This diversity, coupled with variations in cultural and dietary practices, genetic predispositions, and socioeconomic factors, restricts the broader applicability of the findings.

Methodological variability among the included studies also poses a challenge. While some lacked control groups, others used placebos, active comparators, or required participants to maintain existing pharmacotherapy. Moreover, conflicts of interest and inconsistencies in the dosage, duration, and formulation of the interventions further contributed to the observed heterogeneity in effect sizes.

### 4.4 Future Directions for Practice and Research

Despite highlighting safety concerns related to herbal medicine use, there is a significant lack of properly documented adverse event data. Further research is necessary to fill the gaps identified in this review, particularly in terms of comprehensive reporting of adverse events and long‐term participant follow‐up. In addition, when weighing the risks and benefits of phytotherapy, prescribers and users should not only consult the adverse events reported in this scoping review but also pharmacovigilance databases, government reports, online resources, conference proceedings, and case reports.

It is important to note that pharmacological treatments for weight loss are also associated with a range of adverse drug events. A meta‐analysis of 28 randomized controlled trials, involving 29,018 overweight and obese patients, reported that the use of these medications is associated with a 1.3 to 2.9 times higher odds of discontinuation due to adverse drug events (Khera et al. 2016). Future studies should comprehensively compare the safety profiles of herbal and pharmacological treatments, considering both short‐ and long‐term outcomes, to provide evidence‐based guidance for users and healthcare providers.

From a real‐world perspective, our findings challenge the assumption that herbal medicine products are entirely harmless, highlighting the critical role of health literacy in promoting the reporting of adverse events associated with their use. Detecting signs linked to the use of these products is essential for ensuring user safety (Ekor 2014) and addressing shortcomings in pharmacovigilance systems, particularly the underreporting of mild and moderate adverse events (Varallo et al. 2019), especially those associated with herbal medicines.

Thus, our findings provide a basis for evaluating the risk–benefit profile of incorporating herbal medicines into treatment protocols, especially in regions where traditional use is widespread.

## 5 Conclusion

Several herbal medicines were identified in this review, including *Camellia sinensis* , *Ephedra sinica* , *Garcinia cambogia*, *Citrus* sp., and *Glycyrrhiza* sp., which were frequently studied in the context of weight loss. While some studies reported significant reductions in anthropometric indices and improvements in lipid profiles, others did not show consistent effects on weight loss. Among the 74 studies, 10 did not report improvements in any parameter. Adverse events associated with these herbal medicines ranged from mild symptoms (e.g., headache) to more severe reactions (e.g., exacerbation of asthma and increased blood pressure).

These findings underscore the importance of carefully assessing the risk–benefit ratio of herbal medicines for weight loss. Although benefits were observed, the variability in interventions, results, and the occurrence of adverse events highlight the necessity of ensuring both safety and efficacy before integrating these products into health protocols.

Future research should address the controversies regarding the efficacy of these interventions and document adverse events more comprehensively. Besides, long‐term studies are necessary to better understand their safety.

## Author Contributions

**Marcela Forgerini:** conceptualization, data curation, formal analysis, investigation, methodology, project administration, supervision, validation, visualization, writing – original draft, writing – review and editing. **Geovana Schiavo:** conceptualization, data curation, formal analysis, investigation, methodology, visualization, writing – original draft, writing – review and editing. **Osvaldo Galo Neto:** data curation, formal analysis, investigation, writing – original draft, writing – review and editing. **Gabriela Barbosa Nascimento:** data curation, formal analysis, investigation, writing – original draft, writing – review and editing. **Johnny Wallef Leite Martins:** data curation, methodology, writing – review and editing. **Patrícia de Carvalho Mastroianni:** conceptualization, funding acquisition, project administration, resources, supervision, validation, writing – review and editing.

## Ethics Statement

The authors have nothing to report.

## Consent

The authors have nothing to report.

## Conflicts of Interest

The authors declare no conflicts of interest.

## Supporting information


**Figure S1:** PRISMA flowchart of the study selection process included in the scoping review.
**Table S1:** Preferred Reporting Items for Systematic reviews and Meta‐Analyzes extension for Scoping Reviews (PRISMA‐ScR) checklist, 2018.
**Table S2:** Search strategies in databases Embase, PubMed, Lilacs, and Scopus.
**Table S3:** Excluded records during the eligibility process (*n* = 45).
**Table S4:** Characteristics of the included studies in this scoping review (*n* = 74).
**Table S5:** Characteristics of funding, pharmacy industry responsible for intervention, ethical and equity considerations, and conflict of interest of the studies included in this scoping review (*n* = 74).
**Table S6:** Use of herbal medicines products associated with improvements, worsening, or without changes in other outcomes (*n* = 53).

## Data Availability

Data were included in the article and in Supporting Information.
