# Supplementary material for: Herbal Medicines for Weight Loss and Lipid Profile Improvement: A Scoping Review of Therapeutic Effects and Safety
Source: Phytother Res. 2025 Sep 14;39(10):4870–912. doi: 10.1002/ptr.70072 (PMC12504802; doi:10.1002/ptr.70072)
Supplement: Supplementary file 1 — Figure S1: PRISMA flowchart of the study selection process included in the scoping review. Table S1: Preferred Reporting Items for Systematic reviews and Meta‐Analyzes extension for Scoping Reviews (PRISMA‐ScR) checklist, 2018. Table S2: Search strategies in databases Embase, PubMed, Lilacs, and Scopus. Table S3: Excluded records during the eligibility process (n = 45). Table S4: Characteristics of the included studies in this scoping review (n = 74). Table S5: Characteristics of funding, pharmacy industry responsible for intervention, ethical and equity considerations, and conflict of interest of the studies included in this scoping review (n = 74). Table S6: Use of herbal medicines products associated with improvements, worsening, or without changes in other outcomes (n = 53). [file PTR-39-4870-s001.docx]

**Herbal medicines for weight loss and lipid profile improvement: a scoping review of therapeutic effects and safety**

***Herbal medicines for weight loss***

Marcela Forgerini ^1^, Geovana Schiavo ^2^, Osvaldo Galo Neto ^3^, Gabriela Barbosa Nascimento ^4^, Johnny Wallef Leite Martins ^5^; Patrícia de Carvalho Mastroianni ^6^.

^1^ Assistant Professor. Ph.D. Department of Drugs and Medicines, School of Pharmaceutical Sciences, São Paulo State University (UNESP), Araraquara, Brazil. ORCID: 0000-0002-2905-8519. E-mail: marcela.forgerini@unesp.br

^2^ Ph.D. student. Department of Drugs and Medicines, School of Pharmaceutical Sciences, São Paulo State University (UNESP), Araraquara, Brazil. ORCID: 0000-0001-9502-6155. E-mail: geovana.schiavo@gmail.com

^3^ Pharmacy graduate student. Department of Drugs and Medicines, School of Pharmaceutical Sciences, São Paulo State University (UNESP), Araraquara, Brazil. ORCID: 0000-0002-2028-5133. E-mail: netogalo10@gmail.com

^4^ Pharmacy graduate student. Department of Drugs and Medicines, School of Pharmaceutical Sciences, São Paulo State University (UNESP), Araraquara, Brazil. ORCID: 0000-0002-2046-3745. E-mail: gabinascimento84@hotmail.com

^5^ Master student. Department of Drugs and Medicines, School of Pharmaceutical Sciences, São Paulo State University (UNESP), Araraquara, Brazil. ORCID: 0000-0002-0391-8656. E-mail: johnny.martins@unesp.br

^6^ Associate Professor. Ph.D. Department of Drugs and Medicines, School of Pharmaceutical Sciences, São Paulo State University (UNESP), Araraquara, Brazil. ORCID: 0000-0001-8467-7278. E-mail: patricia.mastroianni@unesp.br

***Corresponding author**

**Associate Professor**

**Ph.D. Patrícia de Carvalho Mastroianni**

E-mail: patricia.mastroianni@unesp.br

Telephone: (+55 16) 3301-6977

Department of Drugs and Medicines, School of Pharmaceutical Sciences, São Paulo State University (UNESP)

Address: Highway Araraquara - Jaú, Km 01, Campos Ville.

Zip-code: 14800-903

Araraquara, São Paulo, Brazil.

**Summary**

[**Supplementary Figure 1.** PRISMA flowchart of the study selection process included in the scoping review. 4](#_Toc194396488)

[**Supplementary Table 1.** Preferred Reporting Items for Systematic reviews and Meta-Analyzes extension for Scoping Reviews (PRISMA-ScR) checklist, 2018. 6](#_Toc194396489)

[**Supplementary Table 2.** Search strategies in databases Embase, PubMed, Lilacs, and Scopus. 13](#_Toc194396490)

[**Supplementary Table 3.** Excluded records during the eligibility process (n = 45). 15](#_Toc194396491)

[**Supplementary Table 4.** Characteristics of the included studies in this scoping review (n = 74). 20](#_Toc194396492)

[**Supplementary Table 5.** Characteristics of funding, pharmacy industry responsible for intervention, ethical and equity considerations, and conflict of interest of the studies included in this scoping review (n = 74). 38](#_Toc194396493)

[**Supplementary Table 6.** Use of herbal medicines products associated with improvements, worsening, or without changes in other outcomes (n = 53). 74](#_Toc194396494)

[**References** 95](#_Toc194396495)

# **Supplementary Figure 1.** PRISMA flowchart of the study selection process included in the scoping review.

**Identification of studies via databases and registers**

**Identification of studies via other methods**

Records removed before screening:

Duplicate records removed (n = 211)

Records identified from:

PubMed (n = 808)

Scopus (n = 683)

Embase (n = 463)

Lilacs (n = 54)

Records identified from:

Reference of articles (n = 42)

**Identification**

Records excluded

(n = 1,719)

Records screened

(n = 1,797)

Reports not retrieved

(n = 0)

Reports sought for retrieval

(n = 78)

Reports sought for retrieval

(n = 42)

Reports not retrieved

(n = 1)

**Screening**

Reports excluded (n = 25)

Concept (n = 17)

Study design (n = 4)

Language (n = 3)

Population (n = 1)

Reports assessed for eligibility

(n = 42)

Reports excluded

(n = 20)

Concept (n = 14)

Study design (n = 6)

Reports assessed for eligibility

(n = 77)

**Included**

Studies included in review

(n = 74)

Reports of included studies

(n = 74)

*From:* Page MJ, McKenzie JE, Bossuyt PM, Boutron I, Hoffmann TC, Mulrow CD, et al. The PRISMA 2020 statement: an updated guideline for reporting systematic reviews. BMJ 2021;372:n71. doi: 10.1136/bmj.n71. For more information, visit: http://www.prisma-statement.org/

# **Supplementary Table 1.** Preferred Reporting Items for Systematic reviews and Meta-Analyzes extension for Scoping Reviews (PRISMA-ScR) checklist, 2018.

| **SECTION** | **ITEM** | **PRISMA-ScR CHECKLIST ITEM** | **REPORTED ON PAGE #** |
| --- | --- | --- | --- |
| **TITLE** | | | |
| Title | 1 | Identify the report as a scoping review. | Page 1 |
| **ABSTRACT** | | | |
| Structured summary | 2 | Provide a structured summary that includes (as applicable) background, objectives, eligibility criteria, sources of evidence, charting methods, results, and conclusions that relate to the review questions and objectives. | Page 3 |
| **INTRODUCTION** | | | |
| Rationale | 3 | Describe the rationale for the review in the context of what is already known. Explain why the review questions/objectives lend themselves to a scoping review approach. | Pages 4 and 5 – 1st, 2nd, 3rd, 4th, and 5th paragraphs |
| Objectives | 4 | Provide an explicit statement of the questions and objectives being addressed with reference to their key elements (e.g., population or participants, concepts, and context) or other relevant key elements used to conceptualize the review questions and/or objectives. | Page 5 – 6th paragraph |
| **METHODS** | | | |
| Protocol and registration | 5 | Indicate whether a review protocol exists; state if and where it can be accessed (e.g., a Web address); and if available, provide registration information, including the registration number. | Not applied |
| Eligibility criteria | 6 | Specify characteristics of the sources of evidence used as eligibility criteria (e.g., years considered, language, and publication status), and provide a rationale. | Page 6 – Eligibility criteria |
| Information sources* | 7 | Describe all information sources in the search (e.g., databases with dates of coverage and contact with authors to identify additional sources), as well as the date the most recent search was executed. | Page 6 – Information sources and search |
| Search | 8 | Present the full electronic search strategy for at least 1 database, including any limits used, such that it could be repeated. | Page 6 – Information sources and search |
| Selection of sources of evidence† | 9 | State the process for selecting sources of evidence (i.e., screening and eligibility) included in the scoping review. | Pages 6 and 7 – Selection of sources of evidence |
| Data charting process‡ | 10 | Describe the methods of charting data from the included sources of evidence (e.g., calibrated forms or forms that have been tested by the team before their use, and whether data charting was done independently or in duplicate) and any processes for obtaining and confirming data from investigators. | Page 8 – Data charting process and items |
| Data items | 11 | List and define all variables for which data were sought and any assumptions and simplifications made. | Page 8 – Data charting process and items |
| Critical appraisal of individual sources of evidence§ | 12 | If done, provide a rationale for conducting a critical appraisal of included sources of evidence; describe the methods used and how this information was used in any data synthesis (if appropriate). | Pages 8 and 9 – Critical appraisal of individual sources of evidence |
| Synthesis of results | 13 | Describe the methods of handling and summarizing the data that were charted. | Page 9 – Synthesis of results |
| **RESULTS** | | | |
| Selection of sources of evidence | 14 | Give numbers of sources of evidence screened, assessed for eligibility, and included in the review, with reasons for exclusions at each stage, ideally using a flow diagram. | Page 9 – Study selection |
| Characteristics of sources of evidence | 15 | For each source of evidence, present characteristics for which data were charted and provide the citations. | Pages 9 and 10 – Characteristics of the studies and population |
| Critical appraisal within sources of evidence | 16 | If done, present data on critical appraisal of included sources of evidence (see item 12). | Not applied |
| Results of individual sources of evidence | 17 | For each included source of evidence, present the relevant data that were charted that relate to the review questions and objectives. | Pages 9 - 15 – Characteristics of interventions; Characteristics of interventions; Use of herbal medicine products and their effects on anthropometric parameters; Use of herbal medicines products and their effects on biochemical parameters; and Safety of herbal medicines in weight loss |
| Synthesis of results | 18 | Summarize and/or present the charting results as they relate to the review questions and objectives. | Pages 9 - 15 – Characteristics of interventions; Characteristics of interventions; Use of herbal medicine products and their effects on anthropometric parameters; Use of herbal medicines products and their effects on biochemical parameters; and Safety of herbal medicines in weight loss |
| **DISCUSSION** | | | |
| Summary of evidence | 19 | Summarize the main results (including an overview of concepts, themes, and types of evidence available), link to the review questions and objectives, and consider the relevance to key groups. | Page 15 – 1st paragraph |
| Limitations | 20 | Discuss the limitations of the scoping review process. | Pages 19 and 20 – Limitations and strengths of the review |
| Conclusions | 21 | Provide a general interpretation of the results with respect to the review questions and objectives, as well as potential implications and/or next steps. | Page 21– Conclusion |
| **FUNDING** | | | |
| Funding | 22 | Describe sources of funding for the included sources of evidence, as well as sources of funding for the scoping review. Describe the role of the funders of the scoping review. | Pages 21 - 22 – Statements and Declaration |

JBI = Joanna Briggs Institute; PRISMA-ScR = Preferred Reporting Items for Systematic reviews and Meta-Analyses extension for Scoping Reviews.

* Where *sources of evidence* (see second footnote) are compiled from, such as bibliographic databases, social media platforms, and Web sites.

† A more inclusive/heterogeneous term used to account for the different types of evidence or data sources (e.g., quantitative and/or qualitative research, expert opinion, and policy documents) that may be eligible in a scoping review as opposed to only studies. This is not to be confused with *information sources* (see first footnote).

‡ The frameworks by Arksey and O’Malley (6) and Levac and colleagues (7) and the JBI guidance (4, 5) refer to the process of data extraction in a scoping review as data charting*.*

§ The process of systematically examining research evidence to assess its validity, results, and relevance before using it to inform a decision. This term is used for items 12 and 19 instead of "risk of bias" (which is more applicable to systematic reviews of interventions) to include and acknowledge the various sources of evidence that may be used in a scoping review (e.g., quantitative and/or qualitative research, expert opinion, and policy document).

# **Supplementary Table 2.** Search strategies in databases Embase, PubMed, LILACS, and Scopus.

**Embase**

| #1 | ('natural herbal medicine':ti,ab:kw OR 'herb*':ti,ab,kw OR 'natural product':ti,ab,kw OR 'phytochemical':ti,ab,kw OR 'ethnobotanical':ti,ab,kw OR 'medicinal plant':ti,ab,kw) |
| --- | --- |
| #2 | ('Anti-Obesity Agents'/exp OR 'Anti-Obesity Agents':ti,ab,kw OR 'Weight-Loss Agents':ti,ab,kw OR 'Weight Loss'/exp OR 'Weight loss':ti,ab,kw OR 'Weight Reductions':ti,ab,kw) |
| #3 | ([embase]/lim NOT ([embase]/lim AND [medline]/lim)) AND 'human'/de |
| #4 | ('article'/it OR 'article in press'/it OR 'letter'/it OR 'note'/it OR 'review'/it OR 'short survey'/it) |
| #5 | (#1 AND #2 AND #3) AND #4 |

**Pubmed**

| #1 | ("natural herbal medicine"[TIAB] OR "herb*"[TIAB] OR "natural product"[TIAB] OR "phytochemical"[TIAB] OR "ethnobotanical"[TIAB] OR "medicinal plant"[TIAB]) |
| --- | --- |
| #2 | ("Anti-Obesity Agents"[Mesh] OR "Anti-Obesity Agents"[TIAB] OR "Weight-Loss Agents"[TIAB] OR "Weight Loss"[Mesh] OR "Weight loss"[TIAB] OR "Weight Reductions"[TIAB]) |
| #3 | (editorial[PT] OR comment[PT] OR "Case Reports"[PT] OR news[PT] OR "historical article"[PT] OR "systematic review"[PT] OR "Meta-Analysis"[PT] OR congress[PT] OR "Clinical Conference"[PT] OR animals[MH:noexp] NOT (animals[MH:noexp] AND humans[MH])) |
| #4 | (#1 AND #2) NOT #3 |

**Lilacs**

| #1 | ("Drugs, Chinese Herbal") OR ("Plant Extracts") OR ("natural herbal medicine") OR ("herb*") OR ("natural product") OR ("phytochemical") OR ("ethnobotanical") OR ("medicinal plant") |
| --- | --- |
| #2 | (("Obesity") OR ("Overweight") OR ("Anti-Obesity Agents") OR ("Weight Loss")) |
| #3 | (type_of_study:("review")) OR (type_of_study:("meta-analysis")) OR (type_of_study:("news")) OR (type_of_study:("editorial")) OR (type_of_study:("historical article")) |
| #4 | (#1 AND #2) AND NOT #3 |

**Scopus**

| #1 | TITLE-ABS-KEY ("natural herbal medicine" OR "herb" OR "natural product” OR "phytochemical" OR "ethnobotanical" OR "medicinal plant") |
| --- | --- |
| #2 | TITLE-ABS-KEY ("Anti-Obesity Agents" OR "Anti-Obesity Agents" OR "Weight-Loss Agents" OR "Weight Loss" OR "Weight loss" OR "Weight Reductions") |
| #3 | DOCTYPE(bk OR bz OR ch OR cp OR cr OR ed OR no OR re) |
| #4 | TITLE-ABS-KEY ("animal") |
| #5 | (INDEX(medline)) |
| #6 | #1 AND #2 AND NOT #3 AND NOT #4 AND NOT #5 |

# **Supplementary Table 3.** Excluded records during the eligibility process (n = 45).

| **Concept (n = 31)** |
| --- |
| 1. Paucara WGB, Durán MDG, Magariños CLC, Mayta DDM, Grados-Torrez RE, Dávalos ELG. Efecto de un Producto natural a base de Amaranto, Quinua y Tarwi sobre el Perfil Lipídico en Pacientes con Obesidad y Diabetes Mellitus tipo 2. Con-ciencia. 2021 Aug 16;9(1):27–44. 2. Kenig S, Kramberger K, Petelin A, Bandelj D, Arbeiter AB, Višnjevec AM, *et al*. Helichrysum italicum ssp. italicum Infusion Promotes Fat Oxidation in Hepatocytes and Stimulates Energy Expenditure and Fat Oxidation after Acute Ingestion in Humans: A Pilot Study. Plants. 2021 Jul 23;10(8):1516. 3. Venables MC, Hulston CJ, Cox HR, Jeukendrup AE. Green tea extract ingestion, fat oxidation, and glucose tolerance in healthy humans. The American Journal of Clinical Nutrition. 2008 Mar 1;87(3):778–84. 4. Hu M, Zeng W, Tomlinson B. Evaluation of a Crataegus-Based Multiherb Formula for Dyslipidemia: A Randomized, Double-Blind, Placebo-Controlled Clinical Trial. Evidence-Based Complementary and Alternative Medicine. 2014;2014:1–9. 5. Chedraui P, San Miguel G, Hidalgo L, Morocho N, Ross S. Effect of Trifolium pratense-derived isoflavones on the lipid profile of postmenopausal women with increased body mass index. Gynecological Endocrinology. 2008 Jan;24(11):620–4. 6. Kamohara S, Terasaki Y, Horikoshi I, Sunayama S. Safety of a Coleus forskohlii formulation in healthy volunteers. Personalized Medicine Universe. 2015 Jul;4:63–5. 7. Zhang H, Wei J, Xue R, Wu JD, Zhao W, Wang ZZ, et al. Berberine lowers blood glucose in type 2 diabetes mellitus patients through increasing insulin receptor expression. Metabolism. 2010 Feb;59(2):285–92. 8. Yin J, Xing H, Ye J. Efficacy of berberine in patients with type 2 diabetes mellitus. Metabolism. 2008 May;57(5):712–7. 9. Cicero A, Rovati L, Setnikar I. Eulipidemic Effects of Berberine Administered Alone or in Combination with Other Natural Cholesterol-lowering Agents. Arzneimittelforschung. 2011 Dec 21;57(01):26–30. 10. Kong WJ, Wei J, Zuo ZY, Wang YM, Song DQ, You XF, et al. Combination of simvastatin with berberine improves the lipid-lowering efficacy. Metabolism. 2008 Aug;57(8):1029–37. 11. Kalman D, Incledon T, Gaunaurd I, Schwartz H, Krieger D. An acute clinical trial evaluating the cardiovascular effects of an herbal ephedra–caffeine weight loss product in healthy overweight adults. International Journal of Obesity. 2002 Sep 27;26(10):1363–6. 12. Mollace V, Sacco I, Janda E, Malara C, Ventrice D, Colica C, et al. Hypolipemic and hypoglycaemic activity of bergamot polyphenols: From animal models to human studies. Fitoterapia. 2011 Apr;82(3):309–16. 13. Belza A, Toubro S, Astrup A. The effect of caffeine, green tea and tyrosine on thermogenesis and energy intake. European Journal of Clinical Nutrition. 2007 Sep 19;63(1):57–64. 14. Astrup A, Toubro S, Cannon S, Hein P, Madsen J. Thermogenic synergism between ephedrine and caffeine in healthy volunteers: A double-blind, placebo-controlled study. Metabolism. 1991 Mar;40(3):323–9. 15. Dulloo AG, Duret C, Rohrer D, Girardier L, Mensi N, Fathi M, et al. Efficacy of a green tea extract rich in catechin polyphenols and caffeine in increasing 24-h energy expenditure and fat oxidation in humans. The American Journal of Clinical Nutrition. 1999;70(6):1040–5. 16. Mollace V, Scicchitano M, Paone S, Casale F, Calandruccio C, Gliozzi M, et al. Hypoglycemic and Hypolipemic Effects of a New Lecithin Formulation of Bergamot Polyphenolic Fraction: A Double Blind, Randomized, Placebo- Controlled Study. Endocrine, Metabolic & Immune Disorders - Drug Targets. 2019 Feb 7;19(2):136–43. 17. Zeinalian R, Farhangi MA, Shariat A, Saghafi-Asl M. The effects of Spirulina Platensis on anthropometric indices, appetite, lipid profile and serum vascular endothelial growth factor (VEGF) in obese individuals: a randomized double blinded placebo-controlled trial. BMC Complementary and Alternative Medicine. 2017 Apr 21;17(1). 18. Fialho CGO, Moreira AP, Bressan J, Alfenas RCG, Mattes R, Costa NM. Effects of whole peanut within an energy‐restricted diet on inflammatory and oxidative processes in obese women: a randomized controlled trial. Journal of the Science of Food and Agriculture. 2021 Dec 8;102(8):3446–55. 19. Subbarao KV, Naidu ML, K. Gayathri. Effect of Trushnadi Loha on hyper lipidaemia: A clinical study. International journal of research in ayurveda and pharmacy. 2013 Oct 27;4(5):723–31. 20. Esmaillzadeh A, Zakizadeh E, Faghihimani E, Gohari M, Jazayeri S. The effect of purslane seeds on glycemic status and lipid profiles of persons with type 2 diabetes: A randomized controlled cross-over clinical trial. J Res Med Sci. 2015;20:47–53. 21. Chen D, Li C, Michalsen A, Kessler C, Huang Y, Meng J, et al. Modified Ling-Gui-Zhu-Gan decoction combined with short-term fasting improves therapeutic response in type 2 diabetic patients. Eur J Integr Med. 2012;4:e309–14. 22. Hormati A, Tooiserkany F, Mohammadbeigi A, Aliasl F, Moradi Dehnavi H. Effect of an Herbal Product on the Serum Level of Liver Enzymes in Patients with Non-Alcoholic Fatty Liver Disease: A Randomized, Double-Blinded, Placebo-Controlled Trial. Iran Red Crescent Med J. 2019;21. 23. Parsons HA, Baracos VE, Hong DS, Abbruzzese J, Bruera E, Kurzrock R. The effects of curcumin (diferuloylmethane) on body composition of patients with advanced pancreatic cancer. Oncotarget. 2016;7:20293–304. 24. Rostamizadeh P, Asl SMKH, Far ZG, Ahmadijoo P, Mahmudiono T, Bokov DO, et al. Effects of licorice root supplementation on liver enzymes, hepatic steatosis, metabolic and oxidative stress parameters in women with nonalcoholic fatty liver disease: A randomized double‐blind clinical trial. Phytotherapy Research. 2022;36:3949–56. 25. Salacinski AJ, Howell SM, Hill DL, Mauk SM. The Acute Effects of Nonstimulant Over-the-Counter Dietary Herbal Supplements on Resting Metabolic Rate. J Diet Suppl. 2015;13:368–77. 26. Shatylo V, Antoniuk-Shcheglova I, Naskalova S, Bondarenko O, Havalko A, Krasnienkov D, et al. Cardio-metabolic benefits of quercetin in elderly patients with metabolic syndrome. PharmaNutrition. 2021;15:100250. 27. Yusni Y, Meutia F. Action Mechanism of Rosella ( Hibiscus sabdariffa L. ) Used to Treat Metabolic Syndrome in Elderly Women. Evidence-Based Complementary and Alternative Medicine. 2020;2020:1–6. 28. Hayamizu K, Ishii Y, Shigematsu N, Okuhara Y, Tomi H, Furuse M, et al. Safety of Garcinia cambogia Extract in Healthy Men: High-Doses Administration Study I. J Oleo Sci. 2003;52:499–504. 29. Soni KB, Kuttan R. Effect of oral curcumin administration on serum peroxides and cholesterol levels in human volunteers. Indian J Physiol Pharmacol. 1992;36:273–5. 30. Said O, Saad B, Fulder S, Khalil K & Kassis E. Weight Loss in Animals and Humans Treated with “Weigh level”, a Combination of Four Medicinal Plants Used in Traditional Arabic and Islamic Medicine. Evidence-Based Complementary and Alternative Medicine, 2011, 1–6. 31. Ghai R, Saraswat S. Anti-hyperlipidaemic effect of thyme infused green coffee on human subjects. Human Nutrition & Metabolism, 33, 2023, 200199. |
| **Study design (n = 10)** |
| 1. Ko Y, Kim HJ, Kim H, Choi JB, Kwon YD, Jung WS, et al. Exploring the efficacy and safety of herbal medicine on Korean obese women with or without metabolic syndrome risk factors. Medicine. 2020 Jul 10;99(28):e21153. 2. Jegal KH, Ko MM, Kim B, Son MJ, Kim SH. A national survey on current clinical practice pattern of Korean Medicine doctors for treating obesity. PLOS ONE. 2022 Mar 24;17(3):e0266034–4. 3. Ko MM, Kim BY, Son MJ, Jegal KH, Chung WS, Kim S. Korean medicine registry of herbal medicine for weight loss. Medicine. 2022 Jun 10;101(23):e29407. 4. Song J, Shin S, Kim H. Efficacy and safety of HT048 and HT077 for body fat and weight loss in overweight adults. Medicine. 2019 Nov;98(45):e17922. 5. Ahmad W, Ahmad A, Mohammad Daud Ali, Amin G, Sheikh SA, Usmani A, et al. A questionnaire-based study for weight loss by using herbal drugs in Dammam (Eastern Region), Kingdom of Saudi Arabia. Journal of Pharmacy and Bioallied Sciences. 2019 Jan 1;11(3):248–8. 6. Morimoto C, Satoh Y, Hara M, Inoue S, Tsujita T, Okuda H. Anti-obese action of raspberry ketone. Life Sciences. 2005;77(2):194–204. 7. Seo JB, Choe SS, Jeong HW, Park SW, Shin HJ, Choi SM, et al. Anti-obesity effects of Lysimachia foenum-graecum characterized by decreased adipogenesis and regulated lipid metabolism. Experimental and Molecular Medicine. 2011;43(4):205. 8. Shekelle PG, Hardy M, Morton S, Maglione M, Suttorp MJ, Roth E, et al. Ephedra and ephedrine for weight loss and athletic performance enhancement: clinical efficacy and side effects. PubMed. 2003 Mar 1;(76):1–4. 9. Greenway FL. The safety and efficacy of pharmaceutical and herbal caffeine and ephedrine use as a weight loss agent. Obesity Reviews. 2001 Aug;2(3):199–211. 10. Klein G, Kim J, Himmeldirk K, Cao Y, Chen X. Antidiabetes and Anti-Obesity Activity of Lagerstroemia speciosa. Evidence-Based Complementary and Alternative Medicine. 2007;4(4):401–7. |
| **Language (n = 3)** |
| 1. Mohsen A, Zeinab H, Rouhollah H, Abbas P. Effect of five weeks circuit resistance training with garlic supplementation on serum levels of Adiponectin in overweight female. Journal of Medicinal Plants. 2017 Apr;4. 2. Avandi M, Madani P, Haghshenas R, Pakdel A. The combined effect of eight weeks high intensity resistance training with ginger supplementation on WHR, body composition and body mass in obese women. Koomesh. 2017 Jun;19. 3. Pazoki AR, Arshadi S. Effect of 8 Week Consumption Flax Seed Supplementation with Endurance Training on BDNF and IGF-1 in Obese Women. Journal of Medicinal Plants. 2019 May 1;2(70):188–96. |
| **Population (n = 1)** |
| 1. Mangal A, Sharma M. Evaluation of certain medicinal plants for antiobesity properties. Indian Journal of Traditional Knowledge. 2009 Oct 1;8(4):602–5. |

# **Supplementary Table 4.** Characteristics of the included studies in this scoping review (n = 74).

| **Study** | **Country** | **Study design** | **Inclusion criteria** | **Local** | **Sample size**  **(N of women)** |
| --- | --- | --- | --- | --- | --- |
| Frati-Munari *et al*., 1983 (1) | MX | NR | NR | Medical Center La Raza, Instituto Mexicano del Seguro Social. | 29 (18) |
| Astrup *et al*., 1992 (2) | DK | DBRCT | Women participants diagnosed with obesity. | NR | 16 (16) |
| Heymsfield *et al*., 1998 (3) | US | DBRCT | Participants (aged 18 to 65) diagnosed with excess weight (BMI between 27 and 38 kg/m^2^). | St Luke’s, Roosevelt Hospital Center. | 135 (116) |
| Ignjatovic *et al*., 2000 (4) | AUS | DBRCT | Participants who were not in use of drugs; or diagnosed with metabolic diseases that could influence with the tests. | Monash University. | 140 (114) |
| Andersen *et al*., 2001 (5) | DK and FR | DBRCT | Participants who had no previous pregnancies; diagnosis of gastrointestinal diseases; in use of drugs; and on a specific diet. | Primary health care practice in France and Medical Center Charlottenlund | 120 (42) |
| Armstrong *et al*., 2001 (6) | US | RCT | Participants (age 18 to 40) with > 20% fat for men or > 30% fat for women (determined using dual-energy x-ray absorptiometry) and sedentary to moderately active (aerobic activity < 3 days/week). | Eastern Michigan University | 26 (20) |
| Boozer *et al*., 2001 (7) | US | DBRCT | Participants (age 25 to 55) with a BMI between 29 and 35 kg/m^2^ with stable weight (± 2.5 kg) for at least 3 months before the study. | College of Physicians and Surgeons, Columbia University. | 67 (57) |
| Sindler *et al*., 2001 (8) | US | QECT | Participants who were seen at the researcher’s office or used BioLean or phentermine and Satiete for at least 4 continuous weeks. | NR | 128 (87) |
| Kovacs *et al*., 2001 (9) | NL | DBRCT | Participants without clinical health conditions (e.g., not diagnosed with diabetes *mellitus*, cardiovascular diseases, or undergoing medical treatment) and with a BMI between 24 and 32 kg/m^2^. | NR | 21 (14) |
| Boozer *et al*., 2002 (10) | US | DBRCT | Participants (age 18 to 80) with a BMI between 25 and ≤ 40 kg/m^2^. | New York Obesity Research Center, St Luke’s-Roosevelt Hospital; Columbia University; and Beth Israel-Deaconess Medical Center, Harvard Medical School. | 167 (137) |
| Badmaev *et al*., 2002 (11) | US | OCT | Participants with a BMI between 25 and 45 kg/m^2^. | Bariatric Clinic at Hilton Head. | 77 (62) |
| Coffey *et al*., 2004 (12) | US | DBRCT | Participants (age 18 to 65) diagnosed with overweight or obesity, BMI between 30.0 and 39.9 kg/m^2^, and appropriate health condition based on the results of medical history, physical examination, 12-lead electrocardiogram, and laboratory tests, according to the study physician. | University of Alabama, Birmingham. | 102 (88) |
| Gonzalez *et al*., 2004 (13) | US | DBCT | Participants (age 20 to 55) diagnosed with overweight or obesity (10 to 20% above average body weight) and BMI ≥ 30 kg/m^2^. | Cayey Campus, University of Puerto Rico. | 30 (NR) |
| Greenway *et al*., 2004 (14) | US | DBRCT | Participants (age 18 to 65) without clinical health conditions with a BMI between 25 and 35 kg/m². | Pennington Biomedical Research Center, Clinical Trials Department, Louisiana State University System, Baton Rouge. | 64 (33) |
| Hioki *et al*., 2004 (15) | JP | DBRCT | Women participants diagnosed with obesity; with impaired glucose tolerance (fasting plasma glucose level < 7.0 mmol/L (126 mg/dL)); and a 2-hour oral glucose tolerance test value ranging between 7.8 and 11.1 mmol/L (140 to 200 mg/dL, respectively). | Outpatient Obesity Clinic of Kyoto Prefectural, University of Medicine. | 85 (85) |
| Preuss et al., 2004 (16) | IN | DBRCT | Participants (age 21 to 50) with BMI between 30 to 50.8 kg/m²; negative pregnancy test; understood the risks and benefits of intervention; had availability to participate in a 30 min supervised walking-exercise program (5 days a week); accepted to be on a diet (vegetarian or non-vegetarian) with approximately 2,000 kcal/day (17% protein, 25% fat, and 58% carbohydrate) divided into 3 meals; completed a standard health questionnaire; and participated in 3 clinic visits at 0, 4, and 8 weeks. | Department of General Medicine, Alluri Sitarama Raju Academy of Medical Sciences, Elluru. | 30 (NR) |
| Hackman *et al*., 2005 (17) | US | DBRCT | Women participants (age 25 to 47) in pre-menopausal with BMI between 27 and 39 kg/m^2^. | Department of Pathology, University of California Davis Medical Center. | 61 (61) |
| Henderson *et al*., 2005 (18) | US | DBRCT | Women participants (age 25 to 47) in pre-menopausal with a BMI between 27 to 39 kg/m^2^. | University of Memphis | 19 (19) |
| Chan *et al*., 2006 (19) | CHN | DBRCT | Participants (25 to 40 years old) with a BMI approximately 28 kg/m^2^ and diagnosed with polycystic ovary syndrome defined by the presence of two of the following characteristics: oligo or anovulation, clinical and/or biochemical evidence of hyperandrogenism, and transvaginal ultrasound features showing more than 12 immature follicles less than 10 mm in each ovary. | Menstrual Disorder Clinic, Queen Mary Hospital | 34 (34) |
| Dellalibera *et al*., 2006 (20) | FR | RCT | Participants with an overweight problem (BMI > 25 kg/m^2^), homogeneous in weight and muscle mass/fat mass ratio, and who accepted to be on a bland low caloric diet. | NR | 50 (NR) |
| Diepvens *et al*., 2006 (21) | NL | DBRCT | Participants with good health, who were not in using of drugs, moderate caffeine users (200 – 400 mg caffeine/day), non-smokers, normotensive, and at most moderate alcohol users. | Department of Human Biology, Maastricht University | 46 (NR) |
| Greenway *et al*., 2006 (22) | US | RCT | Women participants (age 18 to 60) with a BMI between 25 and 35 kg/m^2^, non-pregnant, non-lactating, and who were not in use of chronic medication (except oral contraceptives or hormone replacement therapy) | Pennington Biomedical Research Center, Clinical Trials Department, Louisiana State University System. | 24 (24) |
| Greenway *et al*., 2006 (23) | US | DBRTC | Participants (age 18 to 60) without clinical health conditions with a BMI between 25 and 40 kg/m^2^. | Pennington Biomedical Research Center, Baton Rouge, Louisiana | 28 (27) |
| Nagao *et al*., 2007 (24) | JP | DBRCT | Participants (age 25 to 55) with a BMI between 24 and 30 kg/m^2^ and/or a waist circumference of 80 to 94 cm who were considered to be visceral fat-type obese but had not been treated at an outpatient department and had no serious liver or renal disease. | Seven medical institutions in the Kanto District. | 270 (114) |
| Nagao *et al*., 2008 (25) | JP | DBRCT | Participants diagnosed with type 2 diabetes *mellitus* who were not using insulin, and pharmacotherapy and diet would not undergo changes. | Isozaki Clinic (Kasai), Yuwakai Nagao Clinic (Amagasaki), and Mizuno Clinic (Daito). | 50 (31) |
| Auvichayapat *et al*., 2008 (26) | THAI | RCT | Men participants (age 40 to 60) or women participants (in postmenopausal for more than 1 year) with a BMI > 25 kg/m^2^. | Faculty of Medicine, Khon Kaen University. | 60 (42) |
| Hsu *et al*., 2008 (27) | TW | DBRCT | Women participants (age 16 to 60) without clinical health conditions with a BMI > 27 kg/m^2^. It is worth noting that the average age of the participants was over 18 years old, justifying the inclusion of this study in this review. | Taipei Hospital. | 100 (100) |
| Kim *et al*., 2008 (28) | KP | DBRCT | Women participants (age 21 and 50) in pre-menopausal with a BMI > 25 kg/m² and weight-stable within ± 3 kg during the previous 6 months; non-smoker; were moderately sedentary housewives or office-workers who did not exercise regularly and walked less than 30 minutes a day. | Bundang CHA Hospital. | 125 (125) |
| Belcaro *et al*., 2009 (29) | IT | SBRCT | Participants with BMI ≥ 25 kg/m^2^ and fasting blood sugar < 126 mg/dL (7 mmol/L). | Gabriele D'annunzio University | 50 (21) |
| Egert *et al*., 2009 (30) | GE | DBRCCT | Participants (age 25 to 65) diagnosed with metabolic syndrome (central obesity (waist circumference ≥ 94 cm for men and ≥ 80 cm for women), serum concentration of triglyceride ≥ 1500 mg/L (1,7 mmol/L), and/or serum concentration of C-reactive protein ≥ 2,0 mg/L); with a BMI between 25 and 35 kg/m^2^; who participated of a screening, which included physical assessments, clinical, medical anamneses, and dietary questionnaire. | Christian Albrechts University, Kiel. | 96 (54) |
| Tominaga *et al*., 2009 (31) | JP | DBRCT | Participants with a BMI between 24 and 30 kg/m^2^. | Kaiyuu Clinic, Tokyo. | 84 (28) |
| Stendell-Hollis *et al*., 2010 (32) | US | DBRCT | Participants (age 18 - 80) with a BMI between 25 and 40 kg/m^2^; were in use of chemotherapy (neo-adjuvant or adjuvant with any medically-prescribed agent/regime) for treatment of invasive breast cancer; non-smoker (at least 12 months ago); without chronic health conditions, such as diabetes *mellitus* or cardiovascular disease; were in use of drugs to control blood glucose and/or blood lipids; or cancer other than the previously treated breast cancer; and agreed to abstain from all diets and weight loss supplements for the 6-month study period. | University of Arizona, Tucson. | 74 (74) |
| Bell *et al*., 2011 (33) | US | DBRCT | Participants (aged 20 to 53) with overweight or obesity according to BMI and were active (2 to 4 days per week of moderate exercise). | University of Memphis. | 45 (NR) |
| Blom *et al*., 2011 (34) | US | DBRCT | Women participants (age 18 to 50) with good health conditions (medical history, physical examination, electrocardiogram, and laboratory tests) and had stable body weight for 2 months and a percentage body fat between 25 and 45% (altered from 25% to 40% during the recruitment phase) as measured by dual-energy X-ray absorptiometry. | Covance Clinical Research Unit (Madison) | 49 (49) |
| Kamiya *et al.,* 2011 (35) | JP | DBRCT | Participants with a BMI between 23 and 30 kg/m^2^ or a waist circumference larger than 85 cm for men or 90 cm for women; were admitted in a hospital for treatment; with the agreement of a doctor after a clinical inspection; and were not in use of drugs. | Kurume clinical pharmacology clinic (Kurume). | 80 (11) |
| Said *et al*., 2011 (36) | IL | CT | NR | NR | 80 (38%) |
| Hu *et al*., 2012 (37) | US | CT | Participants (age ≥ 18) with a BMI ≥ 30 kg/m^2^ and agreed to be on a diet, do exercises, and all current health habits (smoking and alcohol use) stable during participation in the study. | NR | 10 (5) |
| Kamali *et al*. 2012 (38) | IR | DBRCT | Participants (age 16 to 60) with a BMI between 30 to 50 kg/m^2^. For participants between 50 to 60 years older, having a normal electrocardiography was required. | Tehran University of Medical Sciences and Mostafa Khomeini hospital, Tehran. | 62 (46) |
| Lenon *et al*., 2012 (39) | AUS | DBRCT | Participants (age 18 to 60) with a BMI ≥ 30 kg/m^2^. | RMIT University, Bundoora. | 133 (123) |
| Sengupta *et al*., 2012 (40) | IN | DBRCT | Participants (age 21 to 50) with a BMI ≥ 30 kg/m^2^; and agreed to participate in the exercise-walking program, supervised by a trained exercise specialist, be on a diet with 2,000 kcal per day (meals were provided at free of coast by the study sponsor), complete standard health history questionnaire before recruitment into the study, and participate in five clinical visits (Screening, baseline, 2, 4, and 8 weeks). For women participants, a negative in pregnancy test and to follow a method of birth control for the duration of the study, such as condoms, foams, jellies, diaphragm, and intrauterine device were required. | Alluri Sitarama Raju Academy of Medical Sciences, Eluru, Andhra Pradesh. | 50 (29) |
| Stern *et al*., 2012 (41) | IN | DBRCT | Participants (age 21 to 50) with a BMI between 30 and 40 kg/m^2^. For women participants, a negative in pregnancy test and to follow a method of birth control were required. | Alluri Sitarama Raju Academy of Medical Sciences, Eluru, Andhra Pradesh. | 60 (45) |
| Cho *et al*., 2013 (42) | KP | DBRCT | Participants (age 18 to 70) with a BMI ≥ 23 kg/m^2^. | Chung-Ang University Hospital, Seoul. | 69 (45) |
| Kamohara *et al*., 2013 (43) | JP | OCT | Participants (aged > 20) without clinical health conditions. | NR | 15 (7) |
| Kazemipoor *et al*., 2013 (44) | IR | TBRCT | Women participants (age 20 to 55) diagnosed with overweight or obesity with a BMI between 25 and 39.9 kg/m^2^. | University of Malaya Medical Centre. | 70 (70) |
| Stern *et al*., 2013 (45) | IN | DBRCT | Participants (age 21 to 50) with a BMI between 30 and 40 kg/m^2^; and agreed to participate in the exercise-walking program, supervised by a trained exercise specialist, be on a diet with 2,000 kcal per day (meals were provided at free of coast by the study sponsor), complete standard health history questionnaire before recruitment into the study, and participate in five clinical visits (Screening, baseline, 2, 4, and 8 weeks). For women participants, a negative in pregnancy test and to follow a method of birth control for the duration of the study, such as condoms, foams, jellies, diaphragm, and intrauterine device were required. | Alluri Sitarama Raju Academy of Medical Sciences, Eluru, Andhra Pradesh. | 100 (72) |
| Tripathy *et al*., 2013 (46) | IN | DBRCT | Participants (age 18 to 60) with a BMI between > 25 and 35 kg/m^2^. | IPGA and R and S SP Hospital, Kolkata. | 110 (48) |
| Lopez *et al*., 2013 (47) | US | DBRCT | Participants with a BMI greater than 27 kg/m^2^ and body fat greater than 20% (for men) or greater than 25% (for women) and participated in ≥ 2 exercise sessions (aerobic or anaerobic activity) per week during the previous 30 days. | The Center for Applied Health Sciences. | 70 (26) |
| Park *et al*., 2013 (48) | KP | DBRCT | Participants diagnosed with obesity (BMI ≥ 25 kg/m^2^ and waist-hip ratio ≥ 0.90 for men or ≥ 0.85 for women) according to Asia-pacific guideline and had not been diagnosed with any other clinical condition. | Clinical Trial Center for Functional Foods of Chonbuk National University Hospital (Jeonju) | 80 (48) |
| Park *et al*., 2013 (49) | KP | DBRCT | Participants diagnosed with obesity (BMI ≥ 30 kg/m^2^ or BMI 27 to 30 kg/m^2^) with properly controlled hypertension; noninsulin-dependent diabetes *mellitus* of fasting blood glucose < 7.8 mmol/L (140 mg/dL); properly treated hyperlipidemia (≥ 236 mg/dL total cholesterol or ≥ 150 mg/dL triglycerides). | Four tertiary university hospitals. | 113 (95) |
| Woodgate *et al*., 2013 (50) | CAN | DBRCT | Participants (age 20 to 50) with a BMI ≥ 30 kg/m^2^. | University of Guelph. | 24 (17) |
| Chong *et al*, 2014 (51) | GE | DBRCT | Participants with a BMI between 25 and 32 kg/m^2;^ stable body weight for the past 3 months; agreed to be on a diet and eat 3 meals a day; were not in use of weight loss products; and for women participants agreed to use birth control. | Practice for General Medicine, Kurfürstendamm, Berlin | 91 (62) |
| Kim *et al*., 2014 (52) | KP | CT | Women participants (age 40 to 65) diagnosed with obesity (BMI ≥ 25 kg/m²) and weight stable within ± 10% during the recent six months; and were not in use of antibiotics, probiotics, or any drug that could impact their weight or gut microbiota for the last three months. | Dongguk University. | 9 (9) |
| Landor *et al*., 2014 (53) | US | SBRCT | Participants (age 18 to 64) with normal weight (BMI between 18.5 and 24.99 kg/m²), overweight (BMI between 25 and 29.99 kg/m²), obese (BMI between 30 and 34.99 kg/m²), and very obese (BMI ≥ 35 kg/m²). | Sponsor’s facility Desert Labs, Ltd. | 204 (85) |
| Lee *et al*., 2014 (54) | KP | DBRCT | Participants (age 19 to 65) with normal BMI >25 kg/m^2^ and waist circumference >85 cm. | Dongguk University Oriental medical hospital | 50 (NR) |
| Zhou *et al*., 2014 (55) | CHN | DBRCT | Participants (age 18 to 60) with a BMI between 28 and 40 kg/m², waist circumference ≥ 85 cm (men) or ≥ 80 cm (women), and Traditional Chinese Medicine diagnosed for the “qi and phlegm stasis” syndrome. | Five hospitals in Beijing. | 140 (74) |
| Khazaal *et al*., 2015 (56) | IQ | SBRCT | Women participants (aged 20 to 40) diagnosed with obesity with a BMI ≥ 30. | Obesity Research & Therapeutic Center in Al – Kindy Medical College | 60 (60) |
| Kim *et al*., 2015 (57) | KP | DBRCT | Participants diagnosed with obesity (BMI 35 and 25 kg/m² and waist-hip ratio ≥ 0.90 for men or ≥ 0.85 for women) according to Asia-pacific guideline and had not been diagnosed with any other clinical condition. | Clinical Trial Center for Functional Foods (CTCF2), Chonbuk National University Hospital (Jeonju). | 30 (26) |
| Mirtaheri *et al*., 2015 (58) | IR | DBRCT | Participants (age 30 to 60) with a BMI > 25 kg/m². | Tabriz University of Medical Sciences, Tabriz. | 64 (37) |
| Cho *et al*., 2016 (59) | KP | DBRCT | Participants (age ≥ 20) diagnosed with obesity (BMI between 25 and 30 kg/m²) and not diagnosed with diabetes *mellitus*. | Kyung Hee University Hospital, Gangdong, Seoul; | 44 (22) |
| Cho *et al*., 2017 (60) | KP | DBRCT | Participants (age 19 to 60) BMI between 25 and 29.9 kg/m². | Department of Family Medicine, Seoul Paik Hospital, College of Medicine, Inje University, Mareunnaero, Jung-gu. | 60 (42) |
| Cai *et al*., 2017 (61) | CHN | DBRCT | Participants (age ≥ 50) with fasting blood total triglyceride (TG) or total cholesterol (TC) concentrations higher than normal range (TG: 0.56 – 1.70 mmol/L; TC: 2.8 – 5.7 mmol/L; HDL-C: 0.78 – 1.55 mmol/L; LDL-C: 1.68 – 4.53 mmol/L; and glucose: 3.9 – 6.1 mmol/L). | Tangqiao Community Health Service Center, Pudong New District, Shanghai. | 98 (70) |
| Sukohar *et al*., 2017 (62) | ID | QECT | Women participants (age 25 to 50) employee of the University of Lampung with a BMI ≥ 25 kg/m^2^. | Laboratory of Food Analysis Results, Faculty of Agriculture and Laboratory Clinical Pharmacology, Faculty of Medicine, University of Lampung | 17 (17) |
| Dixit *et al*., 2018 (63) | IN | DBRCT | Participants (age 21 to 50) with BMI between 27 to 29.9 km/m²; not diagnosed with metabolic bone disease, gastrointestinal disease, diabetes *mellitus* (type I or type II), cardiovascular disease, renal disease, or abnormal liver function; were not in use of drugs; non-pregnant; and non-lactating. | Sudeep Diabetes Care Centre, Bengaluru and Krupa Centre for Diabetes and Obesity, Bengaluru. | 140 (82) |
| Kang *et al*., 2018 (64) | KP | CT | Participants with overweight (BMI 23 to 24.99 kg/m²), obesity I (BMI 25 to 29.99 kg/m²), or obesity II (BMI ≥ 30 kg/m²). | Te Public Health Center in Jejusi. | 157 (88) |
| Di Pierro *et al*., 2019 (65) | IT | RCT | NR | Clinic of Allergology and Clinical Immunology (Rome), the Centro Polispecialistico di Ricerca (Rome), and the Terme di Fontecchio, Citta’ di Castello (Perugia). | 100 (44) |
| Leverrier *et al*., 2019 (66) | ES | DBRCT | Participants (aged 18 to 65) with BMI between 30 and 40 kg/m² and waist circumference > 102 cm for men and > 88 cm for women. | Clinical Trials Unit managed by Tecnalia Research & Innovation, situated in Araba University Hospital-Site Txagorritxu, Vitoria (Alava). | 50 (32) |
| Rouhani *et al*., 2019 (67) | IR | RCT | Women participants (age 15 to 40) diagnosed with polycystic ovary syndrome with a BMI ≥ 25 kg/m²). It is worth noting that the average age of the participants was over 18 years old, justifying the inclusion of this study in this review. | Imam Reza Hospital, Mashhad University of Medical Sciences. | 80 (80) |
| Salunke *et al*., 2019 (68) | IN | DBRCT | Participants (age 18 to 60) with BMI ≥ 25 kg/m² and stable body weight (i.e., less than 5 kg change). Participants diagnosed with health conditions, such as dyslipidemia and/or hypertension and/or type 1 or type 2 diabetes *mellitus* in correct use of drug were considered eligible. | Vishwanand Kendra, a Center for Integrative Medicine in Pune. | 120 (73) |
| Cheon *et al*., 2020 (69) | KP | DBRCT | Women participants (age 18 to 65) with a BMI ≥ 30 kg/m² or between 27 and 29.9 kg/m²; having more than one of risk factors such as hypertension (systolic blood pressure ≤ 145 mmHg, and diastolic blood pressure ≤ 95 mmHg), diabetes *mellitus* (adjusted fasting blood glucose < 7.8 mmol/L (140 mg/dL)), and hyperlipidemia (total cholesterol of > 236 mg/dL or triglyceride levels > 150 mg/dL; and agreed to be on a calorie diet during. | Gachon University Gil Medical Center in Incheon, Seoul St. Mary’s Hospital and Sangji University Korean Medicine Hospital in Wonju. | 149 (149) |
| Gupte *et al*., 2020 (70) | IN | OCT | Participants diagnosed with overweight (BMI between 25.0 and 30.0 kg/m²) without or with controlled health conditions, such as dyslipidemia, hypertension, and type 2 diabetes *mellitus*. | Obesity-Diabetes Lab, Interactive Research School for Health Affairs, Bharati Vidyapeeth, Pune. | 18 (8) |
| Hancke *et al*., 2021 (71) | IN | DBRCT | Participants (age 18 to 60) with a BMI between 25 and 35 kg/m²; normal liver and renal function; and five metabolic risks had at least three of the following according to the American Heart Association/National Heart, Lung, and Blood Institute: (i) waist circumference: India: men: > 94 cm, women: > 80 cm; United States: men: >102 cm, women: > 89 cm; (ii) triglycerides >150 mg/dL; (iii) blood pressure: systolic: ≥ 130 mmHg and/or diastolic: ≥ 85 mmHg; fasting blood glucose level ≥ 100 mg/dL; and (iv) low high-density lipoprotein (HDL) level: men: < 40 mg/dL, women: < 50 mg/dL. | Clinical Development, Vedic Lifesciences Pvt. Ltd., Mumbai. | 88 (67) |
| Liu *et al*., 2021 (72) | CHN | DBRCT | Participants diagnosed with metabolic Syndrome confirmed by the diagnostic criteria of the Chinese Medical Association Diabetes Association. | Changhai Hospital. | 62 (34) |
| Bagchi *et al*., 2022 (73) | US | CT | NR | Bonita Springs. | 9 (6) |
| Cao *et al*., 2023 (74) | CHN | DBRCT | Participants (aged 18 to 60) with a BMI between 28 and 48 kg/m² and stable body weight within the past 3 months; agreed to do physical activity and follow healthy diet guidelines; were not diagnosed with cardiovascular, hepatic, or renal disease. | NR | 40 (14) |

**NR:** Not reported.

**BMI:** Body mass index.

**Country:** AUS: Australia; CAN: Canada; CHN: China; DK: Denmark; EG: Egypt; ES: Spain; GE: Germany; ID: Indonesia; FR: France; IL: Israel; IN: India; IQ: Iraq; IR: Iran; IT: Italy; JP: Japan; KP: South Korea; NL: Netherlands; MX: Mexico; THAI: Thailand; TW: Taiwan; UA: Ukraine; US: United States. **Study design:** CC: case control; CT: clinical trial; DBCT: double-blinded clinical trial; DBRCT: double-blinded randomized clinical trial; DBRCCT: double-blinded randomizes cross-over clinical trial; OCT: open clinical trial; QECT: quasi experimental clinical trial (non-randomized); RCT: randomized clinical trial; RCCT: randomized cross-over clinical trial; SBCT: single-blinded clinical trial; SBRCT: single-blinded randomized clinical trial; TBRCT: triple-blinded randomized clinical trial. **N:** number of participants.

In the studies by Hsu *et al*., 2008 (27) and Rouhani *et al*., 2019 (67), only participants over 18 years of age were included.

# **Supplementary Table 5.** Characteristics of funding, pharmacy industry responsible for intervention, ethical and equity considerations, and conflict of interest of the studies included in this scoping review (n = 74).

| **Study** | **Funding or acknowledgement** | **Pharmacy industry responsible for intervention** | **Ethical and equity considerations** | **Conflict of interest** |
| --- | --- | --- | --- | --- |
| Frati-Munari *et al*., 1983 (1) | Acknowledgements to María del Carmen Armida (Departamento de Dietología, Hospital de Especialidades, Centro Médico La Raza, Instituto Mexicano del Seguro Social) for her collaboration. | NA | NR | NR |
| Astrup *et al*., 1992 (2) | The study was partially supported by Danish Medical Research Council (Grant N° 12-9084) and Danish Veterinary and Agricultural Research Council (Grant N° 13-4268). | *Curcuma longa* was from Bombay Oil Industries Ltd. and was from capsules by Associated Capsules Ltd., Bombay. | NR | NR |
| Heymsfield *et al*., 1998 (3) | The study was supported by National Institutes of Health (grants RR00645 and P30DK26687) and it has been established a contract with Thompson Medical Company, West Palm Beach, Fla, manufacturer of products that include *Garcinia cambogia*. | NA | The study was approved by St Luke’s – Roosevelt Hospital Center, New York. The participants gave written consent prior to participation. | NR |
| Ignjatovic *et al*., 2000 (4) | NR | The herbal medicine product was from Slimax Laboratories. | NR | NR |
| Andersen *et* *al*., 2001 (5) | Acknowledgements to Dr Lasse Hessel (Natures Remedies Ltd, Amersham, UK) for expert guidance, and Dr Anthony Leeds (King's College London) for contributions in discussion and advice. | NA | NR | NR |
| Armstrong *et al*., 2001 (6) | NR | The herbal medicine product was from Cytodyne Technologies (Lakewood, United States) | The study was approved by Eastern Michigan University College of Education Human Subjects Review Committee. | NR |
| Boozer *et al*., 2001 (7) | The study was supported by Science Toxicology and Technology Consulting, San Francisco; Metabolife Inc., San Diego; and National Institutes of Health (grant P30DK 26687). | The herbal medicine product was from Metabolife®, Metabolife Inc. (San Diego, United States) | The study was approved by St Luke’s – Roosevelt Hospital Center, New York. The participants gave written consent prior to participation. |  |
| Sindler *et al*., 2001 (8) | NR | The herbal medicine product was from Wellness International Network. | NR | Bruce Sindler is an independent distributor for Wellness International Network, which markets BioLean and Satiete. Dr. Sindler may earn income from the use of products marketed by Wellness International Network. |
| Kovacs *et al*., 2001 (9) | The study was supported by Novartis Consumer Health, Nyon, Switzerland. | The herbal medicine product SuperCitrimax™ was from EuroChem Feinchemie (Munich, Germany) | The study was approved by the Ethics Committee  of Maastricht University. | NR |
| Boozer *et al*., 2002 (10) | The study was supported by Science Toxicology and Technology Consulting, San Francisco, United States and National Institutes of Health (grant P30DK 26687). | The herbal medicine product was from Science, Toxicology and Technology, San Francisco (United States) containing Ma Huang (NutraTech Inc, Gardena, United States) and Kola nut (Ashland Distribution Corp, Santa Anna, CA, United States). | The study was approved by the Institutional Review Boards of St Luke’s Roosevelt Hospital Center in New York and Beth Israel Deaconess Medical Center in Boston. | NR |
| Badmaev *et* *al*., 2002 (11) | NR | The herbal medicine product was from Sabinsa Corporation (New Jersey, United States) | NR | NR |
| Coffey *et al.,* 2004 (12) | The study was supported by Pinnacle Inc., the manufacturer of the product tested. | The herbal medicine product was from Pinnacle, Inc. | The study was approved by a legally constituted Institutional Review Boards located at RTL, Inc. in Great Neck, New York on May 2, 2001. Written informed consent was obtained from each participant. | NR |
| Greenway *et al.*, 2004 (14) | Acknowledgements to Mary Beth Burnett for manuscript preparation. The study was supported by grants from Science Technology and Toxicology (San Francisco). | The herbal medicine product was from FormuLean (Denver, United States) | NR | NR |
| Gonzalez *et al.*, 2004 (13) | The study was supported by a grant provided by herbal worldwide holding LLC, FL, United States | The herbal medicine product was from Herbal Worldwide Holding (United States) | NR | NR |
| Hioki *et al.,* 2004 (15) | This study was partially supported by a Grant-in-Aid (No.14571106; to TY) for Scientific Research from the Ministry of Education, Culture, Sports, Science and Technology of Japan. | NA | The study was carried out according to the Declaration of Helsinki. The study protocol was approved by the University Review Board of Kyoto Prefectural University of Medicine. Written informed consent was obtained from each participant. | NR |
| Preuss *et al.*, 2004 (16) | NR | The herbal medicine products were from InterHealth Nutraceuticals, Inc. (Benicia, United States) | An Institutional Review Board approval was obtained from Alluri Sitarama Raju Academy of Medical Sciences for this study.  Written informed consent was obtained from each participant. | NR |
| Hackman *et al.,* 2005 (17) | Acknowledges to Kimberley Hansen BS for her clinical skills, and Kimber Stanhope and James Graham for their technical assistance with the endocrine assays. This study was partially supported by an unrestricted gift from AdvoCare International, LP, Carrollton, TX. | The herbal medicine product was from AdvoCare International, LP (Carrollton, United States) | The study was approved by the University of California, Davis Institutional Review Board. Written informed consent was obtained from each participant. | NR |
| Henderson *et al*., 2005 (18) | This study was partially supported by a research grant from Sabinsa Corporation (Piscataway, NJ) in collaboration with ImagiNutrition (Laguna Niguel, CA). | The herbal medicine product Forslean™ was from Sabinsa Corporation (Piscataway, United States) | NR | The investigators independently collected, analyzed, and interpreted the data for this study and have no financial interest in the reported results. |
| Chan *et al*., 2006 (19) | NR | NA | The study was approved by the Joint Institutional Review Board of the University of Hong Kong and the Hospital Authority. | NR |
| Dellalibera *et al.*, 2006 (20) | NR | The herbal medicine product Svetol™ was from Berkem SA (Gardonne, France). | NR | NR |
| Diepvens *et al*., 2006 (21) | This study was supported by Unilever Food and Health Research Institute, Unilever R&D Vlaardingen, Vlaardingen, Netherlands. The SlimIFast products were donated by Unilever Bestfoods Nederland B.V. | The green tea extract was from Sunphenon 100S (Taiyo Kagaku Co. Ltd., Mie, Japan). | The study was approved by the Medical Ethical Committee of Maastricht University. Written informed consent was obtained from each participant. | NR |
| Greenway *et al.*, 2006 (22) | This study was supported by Westlake Partners, Woodland Hills, California and Deseret Laboratories International, St George, Ut, United States. | The herbal medicine product was from Deseret Laboratories International, St George, Ut, United States. | The study was approved by Pennington Biomedical Research Center Institutional Review Board. Written informed consent was obtained from each participant. | NR |
| Greenway *et al.*, 2006 (23) | This study was supported by grants from Science Technology and Toxicology (San Francisco). | The products were from General Nutrition Corporation and Labrada Nutrition. | The study was approved by Pennington Institutional Review Board. Written informed consent was obtained from each participant. | NR |
| Nagao *et al*., 2007 (24) | There was no funding or outside support for this study. | NA | The study was approved by the institutional review board at the Center of Health Examination of Isogo Central & Neurosurgical Hospital. The study was carried out according to the Helsinki Declaration under the supervision of clinical investigators. Written informed consent was obtained from each participant. | NR |
| Nagao *et al*., 2008 (25) | This study was supported by Kao Corporation. | NA | The study was approved by Isozaki Clinic Ethical Committee and conduct with the management of a contract research organization, Aiei Support Corporation (Itami, Japan) | No conflict of interest. |
| Auvichayapat *et al*., 2008 (26) | This study was supported by the Invitation Research Fund (i 50219) of the Faculty of Medicine of Khon Kaen University, Thailand. | The herbal medicine product Herbal One™ was from Herbal One Co., Ltd. (Nakornprathom, Thailand) | The study was approved by the Ethical Committee for Human Experimentation of Khon Kaen University. | NR |
| Hsu *et al*., 2008 (27) | This study was supported by the National Science Council, Taiwan (grant Nº. 95-2320-B-192-001. | NA | The study was approved by the Human Ethics Committee of our hospital. Clinical trial registration Nº NCT00383058. | No conflict of interest. |
| Kim *et al*., 2008 (28) | This study was supported by the Korea Research Foundation Grant funded by the Korean Government  (MOEHRD, Nº R04-2004-000-10023-0) | The herbal medicine product was from Department of Oriental Pharmacology at Bundang CHA Hospital (Bundang, Korea). | The study was approved by the Institutional Review Board of Bundang CHA Hospital. | NR |
| Belcaro *et al*., 2009 (29) | This study was supported by a research grant from Horphag Research UK Ltd. | The herbal medicine product Glucaffect™ was from Reliv Inc. (Chesterfield, United States) | NR | NR |
| Egert *et al*., 2009 (30) | This study was supported by the German Federal Ministry of Education and Research (BMBF 0313856A) within the project ‘*Functional Foods for Vascular* *Health – from Nutraceuticals to Personalised Diets’*. | The herbal product was from Voigt Global Distribution Inc. (Lawrence, United States) | The study was carried out according to the Declaration of Helsinki. The study was approved by Medical Faculty of the Christian-Albrechts, University of Kiel, Germany. Written informed consent was obtained from each participant. | No conflict of interest. |
| Tominaga *et al.*, 2009 (31) | Acknowledge to Dr. Tetsuro Yamamoto and Mr. Muneaki Iizuka of TTC Co., Ltd. for managing the clinical studies and providing valuable input; and to Mr. Tetsutaro Hamano for the statistical analysis. | NA | The study was carried out according to the Declaration of Helsinki. The study was approved by the Ethics Committee of Kaiyuu Clinic (Tokyo, Japan). Written informed consent was obtained from each participant. | No conflict of interest. |
| Stendell-Hollis *et al.*, 2010 (32) | The tea for this research was provided by Unilever, Inc. | The herbal medicine product was from Unilever, Lipton (Unilever Bestfoods Company North America, Englewood, United States) | This study was approved by the University of Arizona Human Subjects Committee. | No conflict of interest. |
| Bell *et al*., 2011 (33) | This study was supported by Kaneka Nutrients and The University of Memphis. | The herbal medicine product Glavonoid™ was from Ultimate Nutrition | The study was approved by the University Institutional Review Board for Human Subjects Research. | RJB has received research funding or acted as consultant to nutraceutical and dietary supplement companies. Other authors declare no competing interests. |
| Blom *et al*., 2011 (34) | This study was supported by Unilever. Phytopharm plc was the co-sponsor of this study. | NA | The study was conducted in accordance with the applicable US Code of Federal Regulations and Good Clinical Practice, which are consistent with the Declaration of Helsinki. | No conflict of interest. |
| Kamiya *et al.,* 2011 (35) | NR | The herbal medicine product was from Ohta’s Isan Co. Ltd. (Ushiku, Japan) | The study was carried out according to the Declaration of Helsinki. The study was approved by the Institutional Review Board of Kurume clinical pharmacology clinic. Written informed consent was obtained from each participant. | NR |
| Said *et al*., 2011 (36) | NR | The herbal medicine product was from Antaki Ltd Laboratories (Kfar Kana, Israel) | NR | NR |
| Hu *et al*., 2012 (37) | Acknowledge to Dr. Bonny Specker at South Dakota State University for her advice on calcitriol discussion part in this manuscript. | The herbal medicine product was from Shaanxi Jintai Biological Engineering Co., Ltd. (Xi’an, China) | This study was conducted in accordance with “good clinical practice” and all applicable regulatory requirements including the Declaration of Helsinki. The study was approved by Avera Institutional Review Board and prior. Written informed consent was obtained from each participant. | NR |
| Kamali *et al*. 2012 (38) | This study was supported by a research grant provided by Shahed University. | NA | The study was carried out according to the Declaration of Helsinki. The study was approved by Shahed University (Nº 112251/4). Trial registration ID in Iranian Registry of Clinical Trials: IRCT201104206237N1). Written informed consent was obtained from each participant. | No conflict of interest. |
| Lenon *et al.*, 2012 (39) | This study was co-funded by the Windermere Foundation Limited, an RMIT Emerging Researcher Grant, and an Australian Acupuncture and Chinese Medicine Association (AACMA) seed grant. | The herbal medicine product was from Sun Ten Pharmaceuticals Co Ltd (Taiwan). | The study was approved by the RMIT University Human Research Ethics Committee, and a Clinical Trial Notification (CTN) application was filed with the Therapeutic Goods Administration (TGA-2007/313), Department for Health and Ageing, Australian Federal Government, Canberra, Australia. The trial has been registered with Australian and New Zealand. | No conflict of interest. |
| Sengupta *et al.*, 2012 (40) | This study is supported by Laila Impex R&D Center, India. | NA | The study was approved by the Alluri Sitarama Raju Academy of Medical Sciences Institutional Review Board. | KS, TG and KVA are employees of Laila Impex R&D Centre, Vijayawada, India. AM is an employee of Alluri Sitarama Raju Academy of Medical Sciences, Eluru, India. KM is an Ayurvedic Physician at Suraksha health village, Vijayawada, India. KVSS is a Professor in Department of Statistics, SV University, Tirupati, India. The authors declared no conflict of interest. |
| Stern *et al*., 2012 (41) | This study is supported by an unrestricted grant from InterHealth Nutraceuticals Inc. Benicia, CA to Judith S. Stern, University of California, Davis. | The herbal product was from InterHealth Nutraceuticals (Benicia, United States) and Laila Nutraceuticals, (Vijayawada, India) | The study was approved by the Alluri Sitarama Raju Academy of Medical Sciences Institutional Review Board. | NR |
| Cho *et al*., 2013 (42) | This study is supported a Grant of Biogreen 21 Project (Nº 20100301061065001030) from Rural Development Administration of Korea and Sookmyung Women’s University 2010 (20100133). | The herbal medicine product was from Namil Farm & Ginseng Co. (Geumsam, Korea) | The study was approved by the Institutional Review Board (IRB) of the Chung-Ang University Hospital (Nº C2010114 (410)). Written informed consent was obtained from each participant. | No conflict of interest. |
| Kamohara *et al*., 2013 (43) | This study is supported by a research grant from the DHC Corporation. | The herbal medicine product was from Forskohlii, DHC Corporation (Tokyo, Japan) | NR | Seika Kamohara is a research advisor for the DHC Corporation laboratory. Somboon Noparatanawong is an employee of the DHC Corporation. |
| Kazemipoor *et al.*, 2013 (44) | This study was partially supported by Research (grant Nº RG108/11SUS), Department of Science & Technology Studies, Faculty of Science, University of Malaya, Kuala Lumpur, Malaysia. | The herbal medicine product was from Baharan Company (Yazd, Iran) | The study was approved by Medical Ethics Committee of the University of Malaya Medical Centre (UMMC) (Nº 925/15).  The study was registered with the clinical-trial.gov protocol registration system (Nº NCT01833377). Written informed consent was obtained from each participant. | NR |
| Stern *et al*., 2013 (45) | This study was supported by an unrestricted grant from InterHealth Nutraceuticals Inc., Benicia, CA, to J.S.S. | The herbal product was from InterHealth Nutraceuticals, Inc. (Benicia, United States) under a license agreement with Laila Nutraceuticals, (Vijayawada, India). | The study was approved by the Alluri Sitarama Raju Academy of Medical Sciences Institutional Review Board. | No conflict of interest. |
| Tripathy *et al.*, 2013 (46) | None. | NA | The study was approved by ‘Institutional Ethics Committee’. | No conflict of interest. |
| Lopez *et al.*, 2013 (47) | The sponsor of this study was Ultimate Wellness Systems, Inc. (Lutz, FL). | The herbal medicine product Metabolism™ was from Eurofins Scientific Inc. (Petaluma, United States) | The study was carried out according to the Declaration of Helsinki. The study was approved by Institutional Review Board (IntegReview, Austin, TX; protocol PRO-002, approved 09/16/2011). | HLL and TNZ have received research funding and/or acted as consultants to raw material suppliers, nutraceutical, and dietary supplement companies, including Ultimate Wellness Systems Inc, and Integrity Nutraceuticals Inc. |
| Park *et al*., 2013 (48) | This study was supported by a grant (PF06212-00) from the Plant Diversity Research Center of the 21st Century Frontier Research Program funded by the Ministry of Education, Science and Technology, and by a grant (A111345) from the Korean Health Technology R&D Project from the Ministry of Health and Welfare, Republic of Korea. | NA | The study was approved by the Functional Foods Institutional Review Board of Chonbuk National University Hospital. | No conflict of interest. |
| Park *et al*., 2013 (49) | This study was supported by a grant of the Korean Health Technology R&D Project, Ministry of Health & Welfare, Republic of Korea (B080037). This study was also partially supported by a grant from the Traditional Korean medicine R&D Project, Ministry of Health and Welfare, Republic of Korea (B110068). | The herbal product was from HANPOONG Pharm & Foods Co. Ltd (Jeonju-si, South Korea) produced by Good Manufacturing Practice (GMP) facilities. | The study was approved by the institutional review board at the Catholic University of Korea Seoul St. Mary’s Hospital, Dongguk University Ilsan Oriental Hospital, Semyung University Oriental Medicine Hospital, and Kyungwon Gil Oriental Medical Hospital. | NR |
| Woodgate *et al.*, 2013 (50) | Acknowledgements to NxCare Inc. (Guelph, Ontario, Canada) for donating Calorie-Care™ and for their input in the study design. | The herbal medicine product was from NxCare Inc. (Guelph, Canada) | The study was approved by Human Ethics Committee of the University of Guelph (Guelph, Ontario, Canada). Written informed consent was obtained from each participant. | Derek E. Woodgate, MSc, is president and owner of NxCare Inc., which produces the dietary supplement containing glucomannan, chitosan, fenugreek, *Gymnema sylvestre*, and vitamin C (trade name Calorie-Care™). |
| Chong *et al*, 2014 (51) | The sponsor of this study was InQpharm Europe Ltd. | The herbal medicine product was from InQpharm Europe Ltd. | The study was carried out according to the Declaration of Helsinki, European Union recommendations for Good Clinical Practice, ICH E6 (R1), ICH E3, and German GCP-V (2004, revised 2006). The study was approved by the ethics committee of the Charité Universitatsmedizin prior to initiation. The study was registered on clinicaltrials.gov (Nº NCT01423617). | Pee-Win Chong and Zhi-Ming Beah are employees of InQpharm Europe Ltd, the sponsor of this study, and were involved in the study design, oversight, and the drafting of the manuscript. Barbara Grube, MD was the principal investigator of the study. Linda Riede is an employee of analyzing and realize GmbH and was the project manager for this trial. |
| Kim *et al*., 2014 (52) | This study was supported by the National Research Foundation of Korea (NRF) funded by the Ministry of Science, ICT & Future Planning (N° 2006-2005173). | The herbal medicine product was from Korea Medicine Biofermentation Co., Ltd. (Andong, Korea) | This study was approved by the Institutional Review Board of Dongguk University Ilsan Hospital (Nº 2012-SR-25). | NR |
| Landor *et al*., 2014 (53) | This study was supported by Desert Labs, Ltd., Kibbutz Yotvata, Israel. | The herbal medicine product was provided by the sponsor in Israel. | NR | Michael Landor was a paid medical consultant. Ari Benami is an employee of Desert Labs, Inc., United States, and Beth Loberant is an employee of Desert Labs, Ltd. These authors have no conflicts of interest. Nitzan Segev is both an employee of and stockholder (as a member of Kibbutz Yotvata) in Desert Labs, Ltd., Israel. |
| Lee *et al*., 2014 (54) | This study was supported by Cell Biotech. | The herbal medicine product was from Cell Biotech. | The study was registered at the Clinical Research Information Service, approved by the Korea National Institute of Health (KCT0000386). | No conflict of interest. |
| Zhou *et al*., 2014 (55) | This study was supported by the National Science Program for Traditional Chinese Medicine Research — Intervention and promotion of applied research in the diabetes community-based medicine (N° 201007004). | The herbal medicine product was from Tasly Pharmaceutical Co., Ltd. | The study was carried out according to the Declaration of Helsinki and Guang’anmen Hospital on Good Clinical Practice. The study was approved by the Ethics Committee of Guang’anmen Hospital. This trial was registered at ClinicalTrials.gov (Nº NCT01142076). | NR |
| Khazaal *et al*., 2015 (56) | None. | The raspberry was provided from Vitatrix LLC (United States) and L-carnitine was from Ultimate Nutrition (United States) | The study was approved by the Research Ethical Committee in Al Nahrain University, College of medicine. Written informed consent was obtained from each participant. | No conflict of interest. |
| Kim *et al*., 2015 (57) | This study was supported by a grant from the MICE project of Jeju Island, Ministry of Knowledge Economy, Republic of Korea. Bluegreenlink Co., Ltd. (www.bglink.co.kr) for kindly providing Yerba Mate and their financial support for the completion of this study. | The herbal medicine product was from Jeju Technopark (Jeju, Korea) | The study was approved by the Institutional Review Board of Chonbuk National University Hospital. The protocol was registered at www.clinicaltrials.gov (NCT01778257). | No conflict of interest. |
| Mirtaheri *et al*., 2015 (58) | Acknowledge to the participants for their cooperation. This study was supported by Nutrition Research Center, Tabriz University of Medical Sciences (Grant N° 9271), Tabriz, Iran. | The herbal medicine product was from Darook pharmacological company (Esfahan, Iran). | The study was approved by the Ethics Committee of Tabriz University of Medical Sciences. The trial was registered on the Iranian Registry of Clinical Trials (www.irct.ir/,IRCT2013062811288N3). Written informed consent was obtained from each participant. | No conflict of interest. |
| Cho *et al*., 2016 (59) | This study was supported by Newtree Co, Ltd, of the Republic of Korea and was partially supported by a grant from Kyung Hee University in 2007 (KHU-20071482). This work was also supported by the Technology Innovation Program of the Industrial Strategic Technology Development Program, 10045275, Development of Functional Food Products for Improving the Metabolic Syndrome using Natural Resources and Extending Global Markets funded by the Ministry of Trade, Industry & Energy, Republic of Korea. | NA | The study was approved by the Institutional Review Board. Written informed consent was obtained from each participant. | Newtree Co, Ltd, did not contribute to the reporting of the results or the preparation of the manuscript. |
| Cho *et al*., 2017 (60) | This study was supported by YuYu Pharma, Inc. | The herbal medicine product was from Central Research Institute, YuYu Pharma, Inc. (Suwon, Korea) | The study was approved by Seoul Paik Hospital, Inje University (IRB no. SIT-2013-335). Written informed consent was obtained from each participant. | The authors declare that JS Kwon, SP Yu, and TG Baik are in paid employment by YuYu Pharma, Inc. (place where the product were manufactured); SP Yu holds stocks in YuYu Pharma, Inc.; they have no other competing interests. |
| Cai *et al*., 2017 (61) | This study was partially supported by DRM Resources (Costa Mesa, California, United States), National Natural Science Foundation of China (7163187), the Social Cognitive and Behavioral Sciences Program of Shanghai Jiao Tong University (14JCRY03), Shanghai Key Discipline Construction Project in Public Health (15GWZK1002). | NA | The study was carried out according to the Declaration of Helsinki. The study was approved by Shanghai Jiao Tong University Institutional Review Board. Written informed consent was obtained from each participant. | No conflict of interest. |
| Sukohar *et al*., 2017 (62) | None. | NA | The study was carried out according to the International Conference of Harmonization -Good Clinical Practices Guideline (ICH-GCP) or as per Declaration of Helsinki guideline. | No conflict of interest. |
| Dixit *et al*., 2018 (63) | This study was supported Laila Nutraceuticals, India and PLT (grant# C007185); KD and DVK were the grant recipients. | The herbal medicine products were Slimvance™/Slendacor™ from PLT Health Solutions (Morristown, United States) | The study was carried out according to the Declaration of Helsinki and Good Clinical Practice. The study protocol was approved by an ethics committee (Bangalore Ethics, Bengaluru, India); the approved protocol was registered (Clinical Trial Registry India/2015/06/005835). | KD and DVK are employees of Krupa Centre for Diabetes and Obesity and Sudeep Diabetes Care Centre, Bengaluru, India. KVA is an employee of Laila Nutraceuticals, India. BAD is an employee of PLT Health Solutions Inc, NJ, United States. |
| Kang *et al*., 2018 (64) | This study was supported by the Bio-Synergy Research Project (NRF-2017M3A9C4065964) of the Ministry of Science, ICT and Future Planning through the National Research Foundation and by Jeju Institute of Korean Medicine (JIKM), Republic of Korea. | NA | The study was carried out according to the Declaration of Helsinki. The study was approved by the Institutional Review Board of Dankook University (DKU 2017-11-001). | No conflict of interest. |
| Di Pierro *et al*., 2019 (65) | NR | The herbal medicine product Monoselect Camellia™ was from GreenSelect Phytosome (Indena; Milan, Italy) developed by Velleja Research (Pontenure, Piacenza, Italy) and manufactured by SIIT (Trezzano S/N, Milan, Italy) | The study was approved by the Territory Ethics Committee. | NR |
| Leverrier *et al*., 2019 (66) | This study was funded by Vidya Herbs (P) Ltd, India. | The herbal medicine product was from Vidya Herbs Pvt. Ltd. (Bangalore, India) | The study was carried out according to the Declaration of Helsinki and International Conference on Harmonization (ICH) Guidelines. The study was approved by Araba University Hospital, Vitoria-Gasteiz. This clinical trial has been authorized by the Basque Country sanitary authorities, Osakidetza and registered as Vidya Europe SAS (Expte.2016-98). | AL, DD, PD, and SPK. are employees of Vidya Herbs Group who funded this pilot study which was carried out by a third independent contract research organization (Tecnalia Research and Innovation). WC received payment from Vidya Herbs Pvt Ltd to conduct the statistical analysis and read the manuscript. |
| Rouhani *et al.*, 2019 (67) | Acknowledge to the professors and scholars who were involved in this project; and Vice-Chancellor for Research of the Mashhad University of Medical Sciences to accompany and grant assignment (grant number: 931324). | The herbal medicine product was from Mashhad medicinal herbs market. | The study was approved by the Ethics Committee of the Mashhad University of Medical Sciences (Nº 931324) and was registered in the Iranian Registry of Clinical Trials (Nº IRCT2015040921671N1). | No conflict of interest. |
| Salunke *et al*., 2019 (68) | Acknowledge to Management and Medical Director of Vishwanand Kendra for their support during the study conduct, M/s Pharmanza Herbals Pvt. Ltd. for supplying the study drug. | The herbal medicine products were from Pharmanza Herbals Pvt. Ltd, Gujrat | The study was carried out according to the Declaration of Helsinki. The study was approved by Ethics Committee of Vishwanand Kendra (Ref No: VKECP/002/2016). The study was registered in Clinical Trial Registry of India (CTRI/2017/11/010409). Written informed consent was obtained from each participant. | No conflict of interest. |
| Cheon *et al*., 2020 (69) | This study was supported by a grant of the Korea Health Technology R&D Project through the Korea Health Industry Development Institute (KHIDI), funded by the Ministry of Health & Welfare, Republic of Korea (grant number: HI11C2134 and HI13C0530). The funder had no role in study design, management, and preparation of the manuscript. | The herbal medicine product was from y Hanpoong Pharm & Foods Co., Ltd (Jeonju, Korea) | The study was approved by Institutional Review Boards (IRBs) of Gil Korean Medical Hospital, Gachon University (11–105), Catholic University of Korea Seoul St. Mary's Hospital (KIRB-00393-002), and the Korean Medical Hospital of Sangji University (SJ IRB 120607). The authors declared no conflict of interest. | No conflict of interest. |
| Gupte *et al*., 2020 (70) | This study was supported by Siddhayu Ayurvedic Research Foundation Pvt Ltd, Nagpur and for providing the study drug. | The herbal product was from Siddhayu Ayurvedic Research Foundation Pvt Ltd, Nagpur. | The study was approved by the Ethics Committee of Bharati Vidyapeeth Deemed, University, College of Ayurved (BVDUCOA/EC/1553/15e16). It was registered in the Clinical Trial Registry of India (CTRI/ 2016/07/007067). Written informed consent was obtained from each participant. | Dr Veena Deo and Dr Bharat Bhushan Shrikhande, from Siddhayu initiated the study concept and are also co-authors. Dr Supriya Bhalerao is a part of JAIM's editorial board and is the corresponding author for this article. Dr Bhalerao was not involved in any review or editorial processes of the manuscript. |
| Hancke *et al*., 2021 (71) | This study was supported by HP Ingredients. | The herbal product was from SUN-PAC Manufacturing Inc. (Florida, United States) | The study was carried out according to the Declaration of Helsinki. The study was approved by Ethical Committee, Aditya (Ahmedabad, Gujarat, India). The study was registered at ClinicalTrials.gov (Nº NCT03973086). | Juan Hancke is scientific advisor of HP Ingredients, United States. The other authors declare that they have no conflict of interest. |
| Liu *et al*., 2021 (72) | This study was supported by the Outstanding Leaders Training Program of the Pudong Health Bureau of Shanghai (Grant N°. PWRI2018-02), and Key Specialty Construction Project of Pudong Health and Family Planning Commission of Shanghai (Grant N° PWZzk2017-29). | The herbal medicine product was from Nanchang Jishun Pharmaceutical Co., Ltd. | This study was approved by the Ethics Committee of Shanghai Changhai Hospital, approval number (Nº CHEC2014-114). Written informed consent was obtained from each participant. | No conflict of interest. |
| Bagchi *et al*., 2022 (73) | This study was supported by Bioscience Division of Victory Nutrition International, Inc., Bonita Springs, FL, United States. | The herbal medicine product was from Victory Nutrition International (VNI), Inc. (Bonita Springs, United States) | Written informed consent was obtained from each participant. There was no report about the Ethics Committee. | Commercial Division of Victory Nutrition International (VNI), Inc., Bonita Springs, United States, is involved in marketing this product. Bagchi D. is an employee of Research and Development Division of VNI Inc., Bonita Springs, United States. |
| Cao *et al*., 2023 (74) | This study was supported by Healthy Freedom LLC and Henan University of Chinese Medicine. | The herbal medicine product was from GMP facility (Tian-jiang Pharmaceutical, Jiangsu, China). | The study was approved by the Medical Ethics Committee of Weifang Wei-En Hospital. Written informed consent was obtained from each participant. | This study shared the US Patent Nº US20160296573A1 Weight loss formulations, methods, and compositions based on Traditional Chinese Medicine by X-ML, DC, and NY. Author DC was employed by Healthy Freedom LLC. Authors NY and K.S are members of General Nutraceutical Technology LLC. The remaining authors declare that the research was conducted in the absence of any commercial or financial relationships that could be construed as a potential conflict of interest. |

**NA:** not applicable. **NR:** not reported.

# **Supplementary Table 6.** Use of herbal medicines products associated with improvements, worsening, or without changes in other outcomes (n = 53).

| **Study** | **Intervention** | **Improvement in other outcomes** | **Worsening in other outcomes** | **Without change in other outcomes** |
| --- | --- | --- | --- | --- |
| **Herbal medicine** | | | | |
| Astrup *et al*., 1992 (2) | 1 pill of 200 mg of caffeine and 20 mg of ephedrine, 1 hour before breakfast, lunch, and dinner. | Decrease in carbohydrate oxidation, increase in lipid and protein oxidation. | NA | NA. |
| Andersen *et al*., 2001 (5) | 3 capsules of 112 mg of Yerbe Maté extract (*Ilex paraguayensis*), 95 mg of Guarana seeds (*Paullinia cupana*), and 36 mg of Damiana leaves (*Turnera diffusa* var. aphrodisiaca) with 20 mL apple juice. After 15 minutes, 400 mL of apple juice was also ingested. | Prolonged gastric emptying time. | NA | NA |
| Boozer *et al.*, 2001 (7) | 2 pills of 40 mg of guarana and 12 mg of Ma Huang, 30 minutes before breakfast, lunch, and dinner. Moderate physical activity and limiting fat intake (10% of calories) were suggested. | Decrease in triglycerides levels. | Increase in blood glucose. | NA. |
| Boozer *et al*., 2002 (10) | 2 pills of Ma Huang (90 mg of ephedrine alkaloids) and Kola nut (120 mg of caffeine) 30 minutes before breakfast, lunch, and dinner. Moderate physical activity and limiting fat intake (30% of calories) were suggested. | Decrease in low-density lipoprotein. Increase in high-density lipoprotein. | NA. | Cholesterol levels and triglycerides levels. |
| Coffey *et al*., 2004 (12) | 2 capsules of 125 mg of Ma Huang (10 mg of 8% ephedra), 250 mg of Kola nut and 100 mg of White willow bark, 3 times a day. | Decrease in cholesterol and triglycerides levels. | NA. | NA. |
| Chan *et al*., 2006 (19) | 6 capsules of 90 mg of Chinese green tea 3 times a day. Recommendations on diet and abstaining from drinking or eating foods containing caffeine were suggested. | NA. | Increase in triglycerides levels. | Insulin resistance, cholesterol levels, low-density lipoprotein levels, and high-density lipoprotein levels. |
| Nagao *et al*., 2007 (24) | Intervention 1: 340 mL of extract *Camellia sinensis* with 583 mg catechins.  Intervention 2: 340 mL of extract *Camellia sinensis* with 96 mg of catechins. | NR. | NR. | Cholesterol levels, triglycerides levels, high-density lipoprotein, free fatty acids, and blood glucose. |
| Auvichayapat *et al*., 2008 (26) | 1 capsule of 250 mg of *Camellia sinensis* after breakfast, lunch, and dinner; and a containing of carbohydrates (65%), protein (15%), and fat (20%). | NA. | Decrease in leptin levels. | NA. |
| Hsu *et al*., 2008 (27) | 1 capsule of 400 mg of green tea extract 3 times a day. | Decrease in low-density lipoprotein and triglycerides levels. Increase in high-density lipoprotein. | NA. | NA. |
| Kim *et al*., 2008 (28) | Intervention 1: 4 capsules of 250 mg of *Ephedra sinica* 30 minutes after breakfast, lunch, and dinner.  Intervention 2: 4 capsules of 250 mg of *Evodia rutaecarpa* 30 minutes after breakfast, lunch, and dinner.  A low-calorie diet of 1,200 kcal/d; and moderate physical activity. | Decrease in cholesterol and triglycerides levels. | NA. | NA. |
| Tominaga *et al*., 2009 (31) | Intervention 1: 1 capsule of 300 mg of *Glycyrrhiza glabra* and 2 placebo capsules per day.  Intervention 2: 2 capsules of 300 mg of *Glycyrrhiza glabra* and 1 placebo capsules per day.  Intervention 3: 3 capsules of 300 mg of *Glycyrrhiza glabra* per day. | Decrease in low-density lipoprotein and cholesterol levels. | NA. | NA. |
| Kamiya *et al*., 2011 (35) | Intervention 1: 1 pill of 100 mg of *Puerariae thomsonii* extract 1 time a day and a diet.  Intervention 2: 1 pill of 200 mg of *Puerariae thomsonii* extract 1 time a day and a diet.  Intervention 3: 1 pill of 300 mg of *Puerariae thomsonii* extract 1 time a day and a diet.  Diet of 2,300 kcal/d for women or 2,650 kcal/d for men. | NA | NA | High-density lipoprotein, low-density lipoprotein, and cholesterol total. |
| Hu *et al*., 2012 (37) | 1 capsule of *Coptis chinensis* (500 mg) 3 times a day. | Decrease in cholesterol levels, triglycerides levels, and total cholesterol. | NA | NA. |
| Kamali *et al*., 2012 (38) | 5 g of *Phyllanthus emblica* L. *Terminalia chebula* Retz.*,* and *Terminalia belerica* Retz. (single unreported doses) powder before breakfast and after dinner. | Decrease in fasting blood glucose. | Decrease in plasma insulin levels | NA. |
| Lenon *et al*., 2012 (39) | 4 capsules of 500 mg of *Camellia sinensis* (40%) *Cassia obtusifolia* (40%)*,* and *Sophora japonica* (20%) 3 times a day. | NA | Increase total cholesterol, triglycerides, low-density lipoprotein, fasting blood glucose, plasma insulin levels and insulin resistance (not statistically significant difference between the two groups). | High-density lipoprotein. |
| Sengupta *et al*., 2012 (40) | 1 capsule of 500 mg of *Moringa olefera* (60%), *Murrya koenigi* (30%), and *Curcuma longa* (10%) 30 minutes before breakfast, lunch, and dinner. | Decrease in fasting blood glucose, triglycerides levels, and low-density lipoprotein- high-density lipoprotein-ratio. Increase in adipocytokines. | NA | NA |
| Stern *et al*., 2012 (41) | 2 capsules of 400 mg of *Sphaeranthus indicus* and *Garcinia mangostana* 30 minutes before breakfast and dinner; and a diet (2,000 kcal/d) containing carbohydrates (61%), protein (14%), and fat (25%). | Decrease in cholesterol and triglycerides levels. Increase in adipocytokines. | NA. | NA |
| Cho *et al*., 2013 (42) | 50 mL of 2,28 g of *Scutellariae Radix* (50%) and *Platycodi Radix* (50%) extract with water 3 times a day before meals. | NA. | NA. | Cholesterol levels, triglycerides levels, high-density lipoprotein, and low-density lipoprotein. |
| Kazemipoor *et al*., 2013 (44) | 30 mL of *Carum carvi* L. (1 kg of seeds in 10 liters of water) 20 minutes before lunch. | NA. | NA. | High-density lipoprotein, low-density lipoprotein, cholesterol levels, and triglycerides levels. |
| Stern *et al*., 2013 (45) | 2 capsules of *Sphaeranthus indicus* and *Garcinia mangostana* (400 mg in 3:1 ratio, respectively) 30 minutes before breakfast and dinner; and a diet (2,000 kcal/d) containing carbohydrates (61%), protein (14%), and fat (25%). | Decrease in cholesterol levels, triglycerides levels, and fasting blood glucose. | NA. | NA. |
| Park *et al*., 2013 (48) | 2 capsules of 225 mg of *Gynostemma pentaphyllum* a day. | NA | NA | Cholesterol levels, triglycerides levels, low-density lipoprotein, and apolipoproteins. |
| Park *et al*., 2013 (49) | 1 pill of 7 g of 3.75 g of *Coicis Semen*, 3.75 g of *Semen Castaneae*, 2.5 g of *Raphanus sativus* L., 1.25 g of *Schisandra chinensis* Baill, 1.25 g of Liriopis tuber, 1.25 g of Ephedra, 1.25 g of *Platycodon grandiflorus*, and 1.25 g of *Acori Tatarinowii* *Rhizoma* extract 3 times a day and a diet (1,200 kcal/d for women and 1,500 kcal/d for men). | NA. | NA. | Cholesterol levels, triglycerides levels, high-density lipoprotein, and low-density lipoprotein. |
| Kim *et al*., 2014 (52) | 4 g *of Ephedra sinica* extract 2 times a day and caloric intake limit of 20 to 25 kcal/kg. | NA. | NA. | Fasting plasma glucose, triglycerides levels, cholesterol levels, and high-density lipoprotein. |
| Zhou *et al*., 2014 (55) | 170 mL of *Citrus aurantium,* rhubarb, coptis, and *Semen cassiae* (unreported doses) decoction 2 times a day. | Decrease in insulin resistance and plasma insulin levels. | NA. | Triglycerides levels, cholesterol levels, low-density lipoprotein, high-density lipoprotein, and fasting blood glucose. |
| Kim *et al*., 2015 (57) | 3 capsules of 35 mg of *Ilex paraguariensis* before breakfast, lunch, and dinner. | Decrease in free fatty acids. | Decrease in high-density lipoprotein. | NA. |
| Mirtaheri *et al*., 2015 (58) | 500 mg of *Glycyrrhiza glabra* extract 30 minutes before breakfast, lunch, and dinner; and a diet with a 500-kcal deficit. | Decrease in cholesterol levels, low-density lipoprotein, and monounsaturated free fatty acid. | NA. | High-density lipoprotein and triglycerides levels. |
| Cho et al., 2016 (59) | 1 pill of 700 mg of *Aster spathulifolius* Maxim 30 minutes after breakfast. | NA. | NA. | Fasting plasma glucose, cholesterol levels, triglycerides levels, high-density lipoprotein, and low-density lipoprotein. |
| Cho *et al*., 2017 (60) | 3 pills of 400 mg of *Imperata cylindrica* Beauvoi (50%), *Citrus unshiu* Markovich (20%), and *Evodia officinalis* (30%) after breakfast and dinner; and a diet with a 500-kcal deficit. | NA. | NA. | Fasting plasma glucose, cholesterol levels, triglycerides levels, high-density lipoprotein, and low-density lipoprotein. |
| Dixit *et al*., 2018 (63) | 1 capsule of 450 mg of *Moringa oleifera* (60%), *Murraya koenigii* L. (30%), and *Curcuma longa* L (10%) before breakfast and dinner; a diet (approximately 1,800 kcal/d); and walk for 30 minutes 5 days/week). | Increase in high-density lipoprotein and adipocytokines. Decrease in low-density lipoprotein, cholesterol levels, triglycerides levels, and serum ghrelin levels. | NA | NA |
| Di Pierro *et al*., 2019 (65) | 1 pill of 150 mg of *Camellia sinensis* L. 2 times a day and a diet (1,350 kcal/d for women or 1,850 kcal/d for men). | Decrease in cholesterol, fasting plasma glucose, and triglycerides levels. | NA. | Low-density lipoprotein, high-density lipoprotein, insulin-like growth fator-1, and plasma insulin levels. |
| Leverrier *et al*., 2019 (66) | 1 capsule of 250 mg of *Helianthus annuus* before breakfast and lunch; and a diet with a 500-kcal deficit. | Decrease in cholesterol levels and low-density lipoprotein. | NA | NA |
| Cheon *et al*., 2020 (69) | 3 g extract of 1.33 g *Ephedra sinica* *Stapf*, 1.33 g of *Angelica gigantis Radix*, 1.33 g *Atractylodis rhizoma Alba*, 3.33 g of *Coicis Semen*, 1 g of *Cinnamomi cortex*, 1 g of *Paeonia lactiflora*, and 0.67 g of *Glycyrrhiza uralensis* after breakfast, lunch, and dinner. | NA. | NA. | Cholesterol levels, low-density lipoprotein, high-density lipoprotein, and triglycerides levels. |
| Gupte *et al*., 2020 (70) | 1 pill of 150 mg of Triphala™ (*Cyperus rotundus, Embelia ribes,* and *Plumbago zeylanica*) and 1 pill of Trimad™ (*Terminalia* *chebula*. *Termenalia* *bellerica,* and 60 mg of *Phyllanthus* *emblica*, 50 mg of *Commiphora mukul*, and 250 mg of *Garcinia cambogia*) 2 times a day before meals. | NA. | NA | Fasting plasma glucose, plasma insulin levels, cholesterol levels, triglycerides levels, low-density lipoprotein, high-density lipoprotein, and adipocytokines. |
| Hancke *et al*., 2021 (71) | Intervention 1: 1 capsule of 400 mg of *Citrus bergamia* Risso (83.33%) and *Eurycoma* *longifolia* (16.67%) before breakfast, lunch, and dinner; a diet (20 kcal/kg), and physical activity.  Intervention 2: 1 capsule of 400 mg of *Citrus bergamia* Risso (83.33%) and *Eurycoma* *longifolia* (16.67%), and 200 mg of methyl crystalline cellulose before breakfast, lunch, and dinner; a diet (20 kcal/kg), and physical activity. | NA. | NA. | Fasting blood glucose, adipocytokines, cholesterol levels, triglycerides levels, high-density lipoprotein, and low-density lipoprotein. |
| Cao *et al*., 2023 (74) | 3 to 5 capsules of *Ganoderma lucidum, Coptis chinensis, Astragalus Membranaceus, Nelumbo nucifera Gaertn*, and *Fructus aurantii* (unreported doses) according to body weight before breakfast, lunch, and dinner. Recommendation of diet and intake of at least 1.5 L of water. | Decrease in fasting plasma glucose, total cholesterol, triglycerides, and low-density lipoprotein. | NA. | NA. |
| **Herbal medicine with food supplement** | | | |  |
| Armstrong *et al*., 2001 (6) | Days 1 and 2 days: 1 pill of 940 mg of guarana extract, 335 mg of Ma Huang, 225 mg of fisetin and magnesium phosphate, 105 mg of white willow bark extract, 85 mg of bitter orange, 50 mg of ginger root, and 40 mg of vitamin A before breakfast and afternoon meal.  Days 3 to 44: 2 pills of the same intervention before breakfast, and afternoon meal. | NA. | NA. | Blood glucose, cholesterol levels, triglycerides levels, high-density lipoprotein, and low-density lipoprotein. |
| Greenway *et al.,* 2004 (14) | Phase 1: 2 pills of 150 mg of Ma Huang, 150 mg of kola nut seed, 100 mg of chromium, 50 mg of green tea (with 5 mg caffeine), 50 mg of adrenal gland, 50 mg of L-phenylalanine, 50 mg of ginger root, 50 mg of fo-ti root, 50 mg of lycii berry fruit, 50 mg of *Siberian ginseng* root, 50 mg of cinnamon bark, 5 mg of zinc, 50 mg of vanadium (aspartate), 30 mg of *Astragalus*, 30 mg of caffeine, 20 mg of magnesium, 20 mg of *Ginkgo* *biloba*, and 10 mg of pyridoxal α-ketoglutarate after resting metabolic rate was measured for 30 minutes of each hour for 2 hours.  Phase 2: 2 pills of 70 mg of caffeine and 24 mg of ephedrine with 3 meals associated with a diet (1,200 kcal/d for women or 1,500 kcal/d for men); and walk for 40 minutes per day.  Phase 3: 2 intervention pills with 3 meals. | NA. | NA. | Cholesterol levels, triglycerides levels, low-density lipoprotein, and high-density lipoprotein. |
| Gonzalez *et al*., 2004 (13) | Intervention 1: 3 capsules (intervention 1, 2, and 3) after breakfast.  Intervention 2: 3 capsules (intervention 1, 2, and 3) 15 minutes before lunch.  Intervention 3: 3 capsules (intervention 1, 2, and 3) in the morning and 1 hour at night or 30 minutes before a light workout.  Intervention 1: vitamin C, vitamin B1, vitamin B2, vitamin B3, vitamin B6, vitamin B9, vitamin B12, panthotenic acid, choline, inositol, 4-aminobenzoic acid, calcium, phosphorus, chromium, vanadium, *Garcinia cambogia*, *Gymnema sylvestre*, white kidney bean extract, and green tea (unreported doses)  Intervention 2: psyllium (51%), chitosan (40%), glucomannan (4%), and pectin (4%).  Intervention 3: complex B vitamins, conjugated linoleic acid, taurine, lecithin, green tea extract, L-carnitine, inositol phosphate, vitamin C, ginger extract, capsaicin (unreported doses) | Decrease in cholesterol and triglycerides levels. | NA. | NA. |
| Hioki *et al*., 2004 (15) | 1 pill of 66.7 g of talcum, 44.4 g of *Scutellariae Radix*, 44.4 g of *Glycyrrhizae Radix*, 44.4 g of *Platycodi Radix*, 44.4 g of *Gypsum Fibrosum*, 44.4 g of *Atractylodis Rhizoma*, 26.7 g of *Schizonepetae Spica*, 26.7 g of *Gardeniae Fructus*, 26.7 g of *Paeoniae Radix*, 26.7 g of *Cnidium Rhizoma*, 26.7 g of *Angelicae Radix*, 26.7 g of *Menthae Herba,* 26.7 g of *Ledebouriellae Radix*, 26.7 g of *Ephedrae Herba*, 26.7 g of *Forsythiae Fructus*, 33.3 g of *Rhei Rhizoma*, 15.6 g of *Natrium Sulphuricum* and 6.7 g of *Zingiberis Rhizom* before breakfast, lunch, and dinner; a diet (1,200 kcal/d); and physical activity (5,000 steps/day). | Decrease in triglycerides levels, cholesterol levels, low-density lipoprotein, plasma insulin levels, and insulin resistance. Increase in high-density lipoprotein. | NA. | Fasting plasma glucose and glycated hemoglobin. |
| Preuss *et al*., 2004 (16) | Intervention 1: 1 pill of 4,667 mg of *Garcinia cambogia* 1 hour before breakfast, lunch, and dinner.  Intervention 2: 1 pill of 4,667 mg of *Garcinia cambogia*, niacin-bound chromium (4mg corresponding to 400g elemental chromium), and 400 mg of *Gymnema sylvestre* 1 hour before breakfast, lunch, and dinner. | Decrease in adipocytokines, cholesterol levels, low-density lipoprotein, and triglycerides levels.  Increase in serotonin levels and high-density lipoprotein. | NA. | NA. |
| Hackman *et al*., 2005 (17) | 1 pill of 2,000 mg of *Garcinia camboja* extract, 550 mg of guarana extract, 500 mg of ephedra extract, 200 mg of oolong tea extract, 50 mg of garlic extract, 50 mg of tulsi extract, 50 mg of eleutherococcus senticosis extract, 20 mg of green tea extract*,* 10 mg of *Ginkgo biloba* extract, 10 mg of *Gymnema sylvestre* extract, 5 mg of silymarin extract, and 5 mg of red wine polyphenols  30 to 45 minutes before breakfast and lunch; 1 pill of vitamins (A, C, D, E, K, B1, B2, B3, B6, B9, and B12), biotin, pantothenic acid, calcium, iron, phosphorus, iodine, magnesium, zinc, selenium, copper, manganese, chromium, molybdenum, chloride, potassium, boron, nickel, silicon, tin, vanadium, lutein, choline, coenzyme Q-10, L-glutahione, L-methionine, bioflavonoids, L-carnitine, taurine, beta-sitosterol and 1 pill of eicosapentaenoic acid and docosahexaenoic acid with lunch or dinner. | Decrease in cholesterol levels, low-density lipoprotein, and triglycerides. Increase in high-density lipoprotein and plasma insulin levels. | Increase in insulin resistance, and heart rate. | Blood glucose, fasting plasma glucose, leptin levels, and adipocytokines. |
| Belcaro *et al*., 2009 (29) | Glucaffect™ powder (15 g of french maritime pine bark extract, 8.78 g of low-fat soy flour, 500 mg of Syzygium cumini, 400 mg of inulin, 250 mg of Pterocarpus marsupium, 150 mg of soy lecithin, 120 mg of Salacia oblonga, 120 mg of guar and xanthan gum each one, 90 mg of acesulfame potassium, 44 mg of ground cinnamon, 4 mg of Lagerstroemia speciosa, 750 mg of Omega-3 fish oils, 100 mg of alpha lipoic acid, 20 mg of coenzyme Q-10, and 12.5 mg of L-Glutathione) into 200 mL of water 4 times a day. | Decrease in fasting plasma glucose and glycated hemoglobin. | NA. | NA. |
| Lopez *et al*., 2013 (47) | 2 pills of Prograde Metabolism™ (1.5 mg of thiamin, 1.7 mg of riboflavin, 20 mg of niacin, 10 mg of vitamin B6 and 200 mcg of B12, 1,000 mcg biotin, 10 mg of pantothenic acid, 200 mcg of chromium and a blend of 1,000 mg with raspberry ketone, caffeine anhydrous, bitter orange, ginger root extract, garlic root extract, cayenne extract, L-theanine and *Piper nigrum*) with breakfast and lunch; a diet with a 500-kcal deficit; and physical activity program. | Decrease in appetite levels. | NA. | Cholesterol levels, high-density lipoprotein, low-density lipoprotein, triglycerides levels, adipocytokines, and leptin levels. |
| Lee *et al*., 2014 (54) | 1 capsule of *Bofutsushosan* and probiotics, 2 times a day, a diet with a caloric intake limited to 20 – 25 kcal/kg, and physical activity program. | NA. | NA. | Blood sugar test, cholesterol levels, high-density lipoprotein, high-density lipoprotein, and triglyceride levels. |
| Cai *et al*., 2017 (61) | 2 capsules of 500 mg of *Citrus bergamia* Risso extract, 820 mg of plant sterol esters and orange oil, 50 mg of vitamin C, 20 mg of vitamin B6, 2000 mcg of vitamin B12 and 800 mcg of folic acid, with meals, 2 times a day. | NA. | NA. | Cholesterol levels, triglycerides levels and low-density lipoprotein. |
| **Herbal medicine and pharmacotherapy** | | | |  |
| Frati-Munari *et al.,* 1983 (1) | Decoction of 100 g of Nopal (*Opuntia* sp.) before breakfast, lunch, and dinner. The participants maintained the use of tolbutamide (antidiabetic drug). | Decrease in cholesterol, low-density lipoprotein, fasting plasma glucose, and triglycerides levels. | NA. | High-density lipoprotein levels. |
| Badmaev *et al*., 2002 (11) | 1 pill of 500 mg of hydroxycitric acid (*Garcinia cambogia*) and 100 mcg of chromium picolinate, 30 minutes before breakfast, lunch, and dinner. | Decrease in triglycerides levels, very low-density lipoprotein, and low-density lipoprotein. Increase in high-density lipoprotein. | NA. | Cholesterol levels. |
| Nagao *et al*., 2008 (25) | Intervention 1: 340 mL of *Camellia sinensis* (583 mg of catechins) and caffeine (72.3 mg) extract 1 hour before dinner.  Intervention 2: 340 mL of *Camellia sinensis* (96 mg of catechins) and caffeine (75 mg) extract 1 hour before dinner.  Participants maintained the use of antidiabetic drugs. | Decrease in cholesterol total levels, and free fatty acids. Increase in plasma insulin levels. | NA. | Blood glucose, and glycated hemoglobin. |
| Egert *et al*., 2009 (30) | 2 capsules of 25 mg of quercetin with breakfast, lunch, and dinner. Participants maintained the use of oral contraceptives, antihypertensive and lipid-lowering drugs. | Decrease in low-density lipoprotein and tumor necrosis factor alpha. | Decrease in high-density lipoprotein. | Cholesterol levels, triglycerides levels, and blood glucose. |
| Stendell-Hollis *et al*., 2010 (32) | 240 mL of *Camellia sinensis* tea (550 to 700 mg of extract and 58.91 mg of catechin per bag) 4 times a day. All received chemotherapy and 56.4% received it in combination with radiotherapy. | Decrease in plasma insulin levels and low-density lipoprotein. Increase in high-density lipoprotein. | NA. | NA. |
| Kang *et al*., 2018 (64) | 1 pill of 6 mg of *Citrus unshiu* 3 times a day. 38 participants were in use of antihypertensive, lipid-lowering, and antidiabetic drugs. | Decrease in cholesterol levels, and low-density lipoprotein. | NA. | NA. |
| Rouhani *et al.,* 2019 (67) | Intervention 1: granulated of *Foeniculum vulgare, Urtica dioica, Daucus carota, Trifolium pratense* and *Curcuma longa* (5 g/d) in a sachet and 2 metformin pills (500 mg) a day.  Intervention 2: 2 pills of 500 mg of metformin a day and 20 electroacupuncture sessions.  Intervention 3: granulated of *Foeniculum vulgare, Urtica dioica, Daucus carota, Trifolium pratense* and *Curcuma longa* (5 g/d) in a sachet, 2 pills of 500 mg of metformin a day, and 20 electroacupuncture sessions. | Decrease in cholesterol total, low-density lipoprotein, insulin resistance, plasma insulin levels, and triglycerides levels. | NA. | NA |
| Liu *et al*., 2021 (72) | 1 pill of *Folium Nelumbinis*, *Radix Salviae Miltiorrhizae*, *Fructus* *Crataegi*, *Folium* *Sennae*, and *Fructus* *Psoraleae* (single unreported doses), 3 times a day. Participants maintained the use of metformin and statins. | Decrease in triglycerides levels. Increase in adipocytokines. | NA; | Insulin resistance, liver function, and renal function. |

**g:** gram; **mcg:** microgram; **mg:** milligram; **NR:** not reported; **NA:** not applied.

# **References**

1. Frati-Munari AC, Fernández-Harp JA, Riva H, Ariza-Andraca R, Carmen Torres M. Effects of nopal (Opuntia sp.) on serum lipids, glycemia and body weight. Arch Invest Med (Mex). 1983;14(2):117–25.

2. Astrup A, Buemann B, Christensen NJ, Toubro S, Thorbek G, Victor OJ, Quaade F. The effect of ephedrine/caffeine mixture on energy expenditure and body composition in obese women. Metabolism. 1992 Jul;41(7):686–8.

3. Heymsfield SB, Allison DB, Vasselli JR, Pietrobelli A, Greenfield D, Nunez C. Garcinia cambogia (Hydroxycitric Acid) as a Potential Antiobesity Agent. JAMA. 1998 Nov 11;280(18):1596.

4. Ignjatovic V, Ogru E, Heffernan M, Libinaki R, Lim Y, Ng F. Studies On The Use Of “Slimax”, A Chinese Herbal Mixture, In The Treatment Of Human Obesity. Pharm Biol. 2000 Jan 10;38(1):30–5.

5. Andersen T, Fogh J. Weight loss and delayed gastric emptying following a South American herbal preparation in overweight patients. Journal of Human Nutrition and Dietetics. 2001 Jun;14(3):243–50.

6. Armstrong WJ, Johnson P, Duhme S. The effect of commercial thermogenic weight loss suplement on body composition and energy expenditure in obese adults. Journal of Exercise Physiology. 2001 May 2;4:28–35.

7. Boozer C, Nasser J, Heymsfield S, Wang V, Chen G, Solomon J. An herbal supplement containing Ma Huang-Guarana for weight loss: a randomized, double-blind trial. Int J Obes. 2001 Mar 18;25(3):316–24.

8. Sindler BH, Sindler BH. Herbal Therapy for Management of Obesity: Observations from a Clinical Endocrinology Practice. Endocrine Practice. 2001 Nov;7(6):443–7.

9. Kovacs EMR, Westerterp-Plantenga MS, Vries M, Brouns F, Saris WHM. Effects of 2-week ingestion of (−)-hydroxycitrate and (−)-hydroxycitrate combined with medium-chain triglycerides on satiety and food intake. Physiol Behav. 2001 Nov;74(4–5):543–9.

10. Boozer C, Daly P, Homel P, Solomon J, Blanchard D, Nasser J, Strauss R, Meredith T. Herbal ephedra/caffeine for weight loss: a 6-month randomized safety and efficacy trial. Int J Obes. 2002 May 17;26(5):593–604.

11. Badmaev V, Majeed M, Conte AA. Open field, physician controlled clinical evaluation of a botanical weight loss formula based on Garcinia cambogia derived (-)hydroxycitric acid. Nutracos. 2002;1(1):10–4.

12. Coffey CS, Steiner D, Baker BA, Allison DB. A randomized double-blind placebo-controlled clinical trial of a product containing ephedrine, caffeine, and other ingredients from herbal sources for treatment of overweight and obesity in the absence of lifestyle treatment. Int J Obes. 2004 Nov 31;28(11):1411–9.

13. González MJ, Miranda-Massari JR, Ricart CM. Effect of a dietary supplement combination on weight management, adipose tissue, cholesterol and triglycerides in obese subjects. P R Health Sci J. 2004 Jun;23(2):121–4.

14. Greenway FL, de Jonge L, Blanchard D, Frisard M, Smith SR. Effect of a Dietary Herbal Supplement Containing Caffeine and Ephedra on Weight, Metabolic Rate, and Body Composition*. Obes Res. 2004 Jul;12(7):1152–7.

15. Hioki C, Yoshimoto K, Yoshida T. Efficacy of Bofu-tsusho-san, an oriental herbal medicine, in obese Japanese women with impaired glucose tolerance. Clin Exp Pharmacol Physiol. 2004 Sep;31(9):614–9.

16. Preuss HG, Bagchi D, Bagchi M, Rao CVS, Satyanarayana S, Dey DK. Efficacy of a novel, natural extract of (–)-hydroxycitric acid (HCA-SX) and a combination of HCA-SX, niacin-bound chromium and Gymnema sylvestre extract in weight management in human volunteers: a pilot study. Nutrition Research. 2004 Jan;24(1):45–58.

17. Hackman RM, Havel PJ, Schwartz HJ, Rutledge JC, Watnik MR, Noceti EM, Stohs SJ, Stern JS, Keen CL. Multinutrient supplement containing ephedra and caffeine causes weight loss and improves metabolic risk factors in obese women: a randomized controlled trial. Int J Obes. 2006 Oct 21;30(10):1545–56.

18. Henderson S, Magu B, Rasmussen C, Lancaster S, Kerksick C, Smith P, Melton C, Cowan P, Greenwood M, Earnest C, Almada A, Milnor P, Bowden TMR, Ounpraseuth S, Thomas A, Kreider RB. Effects of Coleus Forskohlii Supplementation on Body Composition and Hematological Profiles in Mildly Overweight Women. J Int Soc Sports Nutr. 2005 Dec 1;2(2).

19. Chan CCW, Koo MWL, Ng EHY, Tang OS, Yeung WSB, Ho PC. Effects of Chinese Green Tea on Weight, and Hormonal and Biochemical Profiles in Obese Patients With Polycystic Ovary Syndrome—A Randomized Placebo-Controlled Trial. J Soc Gynecol Investig. 2006 Jan 28;13(1):63–8.

20. Dellalibera O, Lemaire B, Lafay S. Le Svetol ®, un extrait de café vert décaféiné, induit une perte de poids et augmente le ratio masse maigre sur masse grasse chez des volontaires en surcharge pondérale. Phytothérapie. 2006 Nov;4(4):194–7.

21. Diepvens K, Kovacs E, Vogels N, Westerterpplantenga M. Metabolic effects of green tea and of phases of weight loss. Physiol Behav. 2006 Jan 30;87(1):185–91.

22.Greenway FL, Liu Z, Martin CK, Kai-yuan W, Nofziger J, Rood JC, Yu Y, Amen RJ. Safety and efficacy of NT, an herbal supplement, in treating human obesity. Int J Obes. 2006 Dec 25;30(12):1737–41.

23. Greenway F, de Jonge-Levitan L, Martin C, Roberts A, Grundy I, Parker C. Dietary Herbal Supplements with Phenylephrine for Weight Loss. J Med Food. 2006 Dec;9(4):572–8.

24. Nagao T, Hase T, Tokimitsu I. A Green Tea Extract High in Catechins Reduces Body Fat and Cardiovascular Risks in Humans. Obesity. 2007 Jun;15(6):1473–83.

25. Nagao T, Meguro S, Hase T, Otsuka K, Komikado M, Tokimitsu I, Yamamoto T, Yamamoto K. A Catechin‐rich Beverage Improves Obesity and Blood Glucose Control in Patients With Type 2 Diabetes. Obesity. 2009 Feb 6;17(2):310–7.

26. Auvichayapat P, Prapochanung M, Tunkamnerdthai O, Sripanidkulchai B orn, Auvichayapat N, Thinkhamrop B, Kunhasura S, Wongpratoom S, Sinawat S, Hongprapas P. Effectiveness of green tea on weight reduction in obese Thais: A randomized, controlled trial. Physiol Behav. 2008 Feb;93(3):486–91.

27. Hsu CH, Tsai TH, Kao YH, Hwang KC, Tseng TY, Chou P. Effect of green tea extract on obese women: A randomized, double-blind, placebo-controlled clinical trial. Clinical Nutrition. 2008 Jun;27(3):363–70.

28. Kim HJ, Park JM, Kim JA, Ko BP. Effect of Herbal Ephedra sinica and Evodia rutaecarpa on Body Composition and Resting Metabolic Rate: A Randomized, Double-blind Clinical Trial in Korean Premenopausal Women. J Acupunct Meridian Stud. 2008 Dec;1(2):128–38.

29. Belcaro G, Cesarone M, Silvia E, Ledda A, Stuard S, G V, Dougall M, Cornelli U, Hastings C, Schönlau F. Daily consumption of Reliv GlucaffectTM for 8 weeks significantly lowered blood glucose and body weight in 50 subjects. Phytotherapy Research. 2009 Dec 25;23(12):1673–7.

30. Egert S, Bosy-Westphal A, Seiberl J, Kürbitz C, Settler U, Plachta-Danielzik S, Wagner AE, Frank J, Schrezenmeir J, Rimbach G, Wolffram S, Müller MJ. Quercetin reduces systolic blood pressure and plasma oxidised low-density lipoprotein concentrations in overweight subjects with a high-cardiovascular disease risk phenotype: a double-blinded, placebo-controlled cross-over study. British Journal of Nutrition. 2009 Oct 14;102(7):1065–74.

31. Tominaga Y, Nakagawa K, Mae T, Kitano M, Yokota S, Arai T, Ikematsu H, Inoue S. Licorice flavonoid oil reduces total body fat and visceral fat in overweight subjects: A randomized, double-blind, placebo-controlled study. Obes Res Clin Pract. 2009 Aug;3(3):169–78.

32. Stendell-Hollis NR, Thomson CA, Thompson PA, Bea JW, Cussler EC, Hakim IA. Green tea improves metabolic biomarkers, not weight or body composition: a pilot study in overweight breast cancer survivors. Journal of Human Nutrition and Dietetics. 2010 Dec;23(6):590–600.

33. Bell ZW, Canale RE, Bloomer RJ. A dual investigation of the effect of dietary supplementation with licorice flavonoid oil on anthropometric and biochemical markers of health and adiposity. Lipids Health Dis. 2011 Dec 10;10(1):29.

34. Blom WA, Abrahamse SL, Bradford R, Duchateau GS, Theis W, Orsi A, Ward CL, Mela DJ. Effects of 15-d repeated consumption of Hoodia gordonii purified extract on safety, ad libitum energy intake, and body weight in healthy, overweight women: a randomized controlled trial. Am J Clin Nutr. 2011 Oct;94(5):1171–81.

35. Kamiya T, Matsuzuka Y, Kusaba N, Ikeguchi M, Takagaki K, Kondo K. Preliminary Research for the Anti-obesity Effect of Puerariae Flos Extract in Humans. Journal of Health Science. 2011;57(6):521–31.

36. Said O, Saad B, Fulder S, Khalil K, Kassis E. Weight Loss in Animals and Humans Treated with “Weighlevel”, a Combination of Four Medicinal Plants Used in Traditional Arabic and Islamic Medicine. Evidence-Based Complementary and Alternative Medicine. 2011;2011:1–6.

37. Hu Y, Ehli EA, Kittelsrud J, Ronan PJ, Munger K, Downey T, Bohlen K, Callahan L, Munson V, Jahnkec M, Marshall LL, Nelson K, Huizenga P, Hansen R, Soundy TJ, Davies GE. Lipid-lowering effect of berberine in human subjects and rats. Phytomedicine. 2012 Jul;19(10):861–7.

38. Kamali SH, Khalaj AR, Hasani-Ranjbar S, Esfehani MM, Kamalinejad M, Soheil O, Kamali SA. Efficacy of ‘Itrifal Saghir’, a combination of three medicinal plants in the treatment of obesity; A randomized controlled trial. DARU Journal of Pharmaceutical Sciences. 2012 Dec 10;20(1):33.

39. Lenon GB, Li KX, Chang YH, Yang AW, Da Costa C, Li CG, Cohen M, Mann N, Xue CCL. Efficacy and Safety of a Chinese Herbal Medicine Formula (RCM-104) in the Management of Simple Obesity: A Randomized, Placebo-Controlled Clinical Trial. Evidence-Based Complementary and Alternative Medicine. 2012;2012:1–11.

40. Sengupta K, Mishra AT, Rao MK, Sarma KV, Krishnaraju A V, Trimurtulu G. Efficacy and tolerability of a novel herbal formulation for weight management in obese subjects: a randomized double blind placebo controlled clinical study. Lipids Health Dis. 2012 Dec 20;11(1):122.

41. Stern JS, Peerson J, Mishra AT, Sadasiva Rao MV, Rajeswari KP. Efficacy and tolerability of a novel herbal formulation for weight management. Obesity. 2012 May;21(5):921–7.

42. Cho SH, Yoon Y, Yang Y. The Evaluation of the Body Weight Lowering Effects of Herbal Extract THI on Exercising Healthy Overweight Humans: A Randomized Double-Blind, Placebo-Controlled Trial. Evidence-Based Complementary and Alternative Medicine. 2013;2013:1–8.

43. Kamohara S, Noparatanawong S. A Coleus forskohlii extract improves body composition in healthy volunteers: An open-label trial. Personalized Medicine Universe. 2013 Jul;2:25–7.

44. Kazemipoor M, Radzi CWJB wan M, Hajifaraji M, Haerian BS, Mosaddegh MH, Cordell GA. Antiobesity Effect of Caraway Extract on Overweight and Obese Women: A Randomized, Triple-Blind, Placebo-Controlled Clinical Trial. Evidence-Based Complementary and Alternative Medicine. 2013;2013:1–8.

45. Stern JS, Peerson J, Mishra AT, Mathukumalli VSR, Konda PR. Efficacy and Tolerability of an Herbal Formulation for Weight Management. J Med Food. 2013 Jun;16(6):529–37.

46.Tripathy PC, Karmahapatra PB, Palaniyamma D. Randomized, double-blind, placebo controlled clinical study to evaluate the effects of Garcinia caplets in obese subjects. International Research Journal of Pharmacy. 2013 Aug 10;4(7):182–6.

47. Lopez HL, Ziegenfuss TN, Hofheins JE, Habowski SM, Arent SM, Weir JP, Ferrando AA. Eight weeks of supplementation with a multi-ingredient weight loss product enhances body composition, reduces hip and waist girth, and increases energy levels in overweight men and women. J Int Soc Sports Nutr. 2013 Jan 3;10(1).

48. Park S, Huh T, Kim S, Oh M, Tirupathi Pichiah PB, Chae S, Cha YS. Antiobesity effect of Gynostemma pentaphyllum extract (actiponin): A randomized, double‐blind, placebo‐controlled trial. Obesity. 2014 Jan 5;22(1):63–71.

49. Park S, Nahmkoong W, Cheon C, Park JS, Jang BH, Shin Y, Kim K, Go H, Song Y, Ko S. Efficacy and Safety of Taeeumjowi-tang in Obese Korean Adults: A Double-Blind, Randomized, and Placebo-Controlled Pilot Trial. Evidence-Based Complementary and Alternative Medicine. 2013;2013:1–10.

50. Woodgate DE, Conquer JA. Effects of a Stimulant-Free Dietary Supplement on Body Weight and Fat Loss in Obese Adults: A Six-Week Exploratory Study. Current Therapeutic Research. 2003 Apr;64(4):248–62.

51. Chong P, Beah Z, Grube B, Riede L. IQP‐GC‐101 Reduces Body Weight and Body Fat Mass: A Randomized, Double‐Blind, Placebo‐Controlled Study. Phytotherapy Research. 2014 Oct 2;28(10):1520–6.

52. Kim BS, Song M young, Kim H. The anti-obesity effect of Ephedra sinica through modulation of gut microbiota in obese Korean women. J Ethnopharmacol. 2014 Mar;152(3):532–9.

53. Landor M, Benami A, Segev N, Loberant B. Efficacy and Acceptance of a Commercial Hoodia parviflora Product for Support of Appetite and Weight Control in a Consumer Trial. J Med Food. 2015 Feb;18(2):250–8.

54. Lee SJ, Bose S, Seo JG, Chung WS, Lim CY, Kim H. The effects of co-administration of probiotics with herbal medicine on obesity, metabolic endotoxemia and dysbiosis: A randomized double-blind controlled clinical trial. Clinical Nutrition. 2014 Dec;33(6):973–81.

55. Zhou Q, Chang B, Chen XY, Zhou SP, Zhen Z, Zhang LL, Sun X, Zhou Y, Xie W, Liu HF, Xu Y, Kong Y, Zhou L, Lian F, Tong X. Chinese Herbal Medicine for Obesity: A Randomized, Double-Blinded, Multicenter, Prospective Trial. Am J Chin Med (Gard City N Y). 2014 Jan 29;42(06):1345–56.

56. Khazaal FAK, Mosah HA, Sahib HB, Hamdi AS. Effect of raspberry ketones and L-carnitine on oxidative stress and body weight in Iraqi obese patients. Int J Pharm Sci Rev Res. 2015;31(2):69–75.

57. Kim SY, Oh MR, Kim MG, Chae HJ, Chae SW. Anti-obesity effects of Yerba Mate (Ilex Paraguariensis): a randomized, double-blind, placebo-controlled clinical trial. BMC Complement Altern Med. 2015 Dec 25;15(1):338.

58. Mirtaheri E, Namazi N, Alizadeh M, Sargheini N, Karimi S. Effects of dried licorice extract with low-calorie diet on lipid profile and atherogenic indices in overweight and obese subjects: A randomized controlled clinical trial. Eur J Integr Med. 2015 May;7(3):287–93.

59. Cho IJ, Choung SY, Hwang YC, Ahn KJ, Chung HY, Jeong IK. Aster spathulifolius Maxim extract reduces body weight and fat mass in obese humans. Nutrition Research. 2016 Jul;36(7):671–8.

60. Cho YG, Jung JH, Kang JH, Kwon JS, Yu SP, Baik TG. Effect of a herbal extract powder (YY-312) from Imperata cylindrica Beauvois, Citrus unshiu Markovich, and Evodia officinalis Dode on body fat mass in overweight adults: a 12-week, randomized, double-blind, placebo-controlled, parallel-group clinical trial. BMC Complement Altern Med. 2017 Dec 28;17(1):375.

61. Cai Y, Xing G, Shen T, Zhang S, Rao J, Shi R. Effects of 12-week supplementation of Citrus bergamia extracts-based formulation CitriCholess on cholesterol and body weight in older adults with dyslipidemia: a randomized, double-blind, placebo-controlled trial. Lipids Health Dis. 2017 Dec 22;16(1):251.

62. Sukohar A, Busman H, Kurniawaty E, Pangestu Catur MMS. Effect of consumption Kemunings leaf (Murraya paniculata (l.) Jack) infuse to reduce body mass index, waist circumference and pelvis circumference on obese patients. Int J Res Ayurveda Pharm. 2017 Apr 25;8(2):75–8.

63. Dixit K, Kamath D V., Alluri K V., Davis BA. Efficacy of a novel herbal formulation for weight loss demonstrated in a 16‐week randomized, double‐blind, placebo‐controlled clinical trial with healthy overweight adults. Diabetes Obes Metab. 2018 Nov 18;20(11):2633–41.

64. Kang S, Song S, Lee J, Chang H, Lee S. Clinical Investigations of the Effect of Citrus unshiu Peel Pellet on Obesity and Lipid Profile. Evidence-Based Complementary and Alternative Medicine. 2018 Sep 19;2018:1–6.

65. Di Pierro F, Menghi AB, Barreca A, Lucarelli M, Calandrelli A. Greenselect Phytosome as an adjunct to a low-calorie diet for treatment of obesity: a clinical trial. Altern Med Rev. 2009 Jun;14(2):154–60.

66. Leverrier A, Daguet D, Calame W, Dhoye P, Kodimule SP. Helianthus annuus Seed Extract Affects Weight and Body Composition of Healthy Obese Adults during 12 Weeks of Consumption: A Randomized, Double-Blind, Placebo-Controlled Pilot Study. Nutrients. 2019 May 15;11(5):1080.

67. Rouhani M, Motavasselian M, Taghipoor A, Layegh P, Asili J, Hamedi SS, Avval SB. Efficacy of a Persian Herbal Remedy and Electroacupuncture on Metabolic Profiles and Anthropometric Parameters in Women with Polycystic Ovary Syndrome: A Randomized Controlled Trial. Galen Medical Journal. 2019 Oct 9;8.

68. Salunke M, Banjare J, Bhalerao S. Effect of selected herbal formulations on anthropometry and body composition in overweight and obese individuals: A randomized, double blind, placebo-controlled study. J Herb Med. 2019 Sep;17–18:100298.

69. Cheon C, Song YK, Ko SG. Efficacy and safety of Euiiyin-tang in Korean women with obesity: A randomized, double-blind, placebo-controlled, multicenter trial. Complement Ther Med. 2020 Jun;51:102423.

70. Gupte P, Harke S, Deo V, Bhushan Shrikhande B, Mahajan M, Bhalerao S. A clinical study to evaluate the efficacy of Herbal Formulation for Obesity (HFO-02) in overweight individuals. J Ayurveda Integr Med. 2020 Apr;11(2):159–62.

71. Hancke J, Srivastava S, Caceres DD, Burgos RA, Alarcon P. An exploratory double‐blind, randomized, placebo‐controlled study to assess the efficacy of CitruSlim on body composition and lipid parameters in obese individuals. Phytotherapy Research. 2021 Dec 18;35(12):7039–49.

72. Liu LY, Zhou L, Liu XZ, Zou DJ. Effect of Hedan Tablets on Body Weight and Insulin Resistance in Patients with Metabolic Syndrome. Obes Facts. 2022;15(2):180–5.

73. Bagchi D, Downs B, Banik S, Bagchi M, Kushner S, Chakraborty S, Morrison BS, Hesson S. Effective body recomposition vs. misconceptions of the traditional weight loss strategies: TRCAP21 - a novel technological breakthrough in body recomposition. Functional Foods in Health and Disease. 2022 Apr 1;12(4):134–50.

74. Cao MZ, Wei CH, Wen MC, Song Y, Srivastava K, Yang N, Shi Y, Miao M, Chung D, Li X. Clinical efficacy of weight loss herbal intervention therapy and lifestyle modifications on obesity and its association with distinct gut microbiome: A randomized double-blind phase 2 study. Front Endocrinol (Lausanne). 2023 Mar 22;14.
